# Supplementary material for: Recovery from recurrent depression with mindfulness-based cognitive therapy and antidepressants: a qualitative study with illustrative case studies
Source: BMJ Open. 2020 Feb 18;10(2):e033892. doi: 10.1136/bmjopen-2019-033892 (PMC7044862; doi:10.1136/bmjopen-2019-033892)
Supplement: Supplementary data [file bmjopen-2019-033892supp001.pdf]

Statement Concerning Reflexivity

Interviews were conducted by members of the PREVENT trial research team (see Kuyken et al., 2015), following training and using a standard protocol and semi-structured interview schedule. Both male and female interviewers gathered data. Interviewers had knowledge of the participants' treatment journeys prior to conducting their 24-month interviews (from reviewing their files and in some cases as a result of their involvement in earlier waves of data collection). The protocol included interviewers familiarizing themselves with any information about treatment experiences and trajectories of participants, which were held in study records, as part of the interview preparation process. Researchers did not know participants prior to their entry to the trial, and had no association with them outside the context of the trial and associated research assessments.

All/Some interviewers had undertaken mindfulness training, acting as participant observers in MBCT courses, or in other contexts. This personal knowledge enabled them to understand the nuances in participants' descriptions of their experiences, for example participants' references to particular mindfulness practices or exercises, and to respond with confidence. Some interviewers had positive personal experiences of mindfulness whereas others held more neutral attitudes. None were aware of the main trial outcomes at the time the interviews were conducted and all were encouraged to adopt an open minded and curious attitude, with no preconceptions about whether MBCT-TS had, or had not, supported participants in their treatment journeys. Despite this, it should be acknowledged that some interviewers may have held implicit biases or expectations regarding treatment effects. Likewise, participants understood the association of interviewers with the primary trial. Thus whilst participants were encouraged to speak freely and honestly about their experiences, it is possible that their responses were influenced by the perceived allegiance of the researchers to

the intervention being explored, and that they may have felt a sense of obligation to make positive comments about MBCT-TS.

The researchers conducting data analysis, AT (BA Hons, Postgraduate Research Assistant) and CC (DPhil, Senior Research Fellow), although not involved in the PREVENT trial, had a knowledge of the programme under investigation. AT has undertaken mindfulness training in other contexts, and is familiar with the MBCT curriculum. CC has significant prior experience as a participant-observer in MBCT classes for people with recurrent depression. JR (DPhil, Lecturer) is an experienced qualitative researcher who has theoretical knowledge of mindfulness-based approaches. AT, CC and JR have all worked previously on studies exploring mindfulness-based programmes in different contexts and AT and CC were employed on a research grant exploring the effectiveness of mindfulness-based programmes at the time this work was conducted. AT, CC, and JR were aware of the main outcomes of the PREVENT trial (no superiority of MBCT-TS over maintenance antidepressants) at the time the qualitative analysis commenced, and approached the data with an assumption of overall equipoise between the two approaches, that was nevertheless likely to concealed marked individual differences in response. WK (PhD, DClinPsy) was the Principal Investigator on the PREVENT trial and is a mindfulness trainer and practitioner. He was not involved in directly teaching mindfulness to any of the participants in the PREVENT trial, but did supervise the mindfulness teachers who taught the MBCT-TS trial classes. He had no personal knowledge of the individual participants and their treatment journeys.
