# Supplementary material for: Recovery from recurrent depression with mindfulness-based cognitive therapy and antidepressants: a qualitative study with illustrative case studies
Source: BMJ Open. 2020 Feb 18;10(2):e033892. doi: 10.1136/bmjopen-2019-033892 (PMC7044862; doi:10.1136/bmjopen-2019-033892)
Supplement: Supplementary data [file bmjopen-2019-033892supp002.pdf]

Consolidated criteria for reporting qualitative studies (COREQ): 32-item checklist

No	Item	Guide questions/description	Responses and reference in manuscript if appropriate
**Domain 1: Research team and reflexivity**			
Personal Characteristics			
1.	Interviewer/facilitator	Which author/s conducted the interview or focus group?	Contributor statement
2.	Credentials	What were the researcher's credentials? *E.g. PhD, MD*	AT, BSc; RB, MD; CC, PhD; FG, PhD; RH PhD; JC DClinPsy; AW DClinPsy; NM, PhD; WK, PhD.
3.	Occupation	What was their occupation at the time of the study?	All researchers. Title page, affiliation.

No	Item	Guide questions/description	Responses and reference in manuscript if appropriate
4.	Gender	Was the researcher male or female?	Female, AT, CC, RH, JC, AW, NM; Male RB, FG, WK.
5.	Experience and training	What experience or training did the researcher have?	Lead authors all had extensive research training, interviewers had interview training and RB, FG, NM and WK all had qualitative methods experience and training. See Methods and Statement Concerning Reflexivity

No	Item	Guide questions/description	Responses and reference in manuscript if appropriate
Relationship with participants			
6.	Relationship established	Was a relationship established prior to study commencement?	Yes. Manuscript Pages 12-13 and Statement Concerning Reflexivity
7.	Participant knowledge of the interviewer	What did the participants know about the researcher? e.*g. personal goals, reasons for doing the research*	Their role in the larger parent trial. See Interview Schedule and Statement Concerning Reflexivity.
8.	Interviewer characteristics	What characteristics were reported about the interviewer/facilitator? e.g. *Bias, assumptions, reasons and interests in the research topic*	See Interview Schedule and Statement

No	Item	Guide questions/description	Responses and reference in manuscript if appropriate
			Concerning Reflexivity.
**Domain 2: study design**			
Theoretical framework			
9.	Methodological orientation and Theory	What methodological orientation was stated to underpin the study? *e.g. grounded theory, discourse analysis, ethnography, phenomenology, content analysis*	Thematic analysis and case studies. Manuscript, Data Analysis) Pages 13-14)
Participant selection			
10.	Sampling	How were participants selected? *e.g. purposive, convenience, consecutive, snowball*	Purposive, see Manuscript, Methods, Participants, Pages 9-11.

No	Item	Guide questions/description	Responses and reference in manuscript if appropriate
11.	Method of approach	How were participants approached? e*.g. face-to-face, telephone, mail, email*	See Manuscript, Study Context and Participants, Pages 9-11.
12.	Sample size	How many participants were in the study?	See Manuscript, Study Context and Participants, Pages 9-11.
13.	Non-participation	How many people refused to participate or dropped out? Reasons?	See Manuscript, Study Context and Participants, Pages 9-11.
Setting			
14.	Setting of data collection	Where was the data collected? e*.g. home, clinic, workplace*	See Manuscript, Study Methods, Pages 9-13 as well as published Study

No	Item	Guide questions/description	Responses and reference in manuscript if appropriate
			Protocol and parent Trial.
15.	Presence of non-participants	Was anyone else present besides the participants and researchers?	No
16.	Description of sample	What are the important characteristics of the sample? *e.g. demographic data, date*	See Manuscript, Study Context and Participants, Pages 9-11.
Data collection			
17.	Interview guide	Were questions, prompts, guides provided by the authors? Was it pilot tested?	See Manuscript, Study Methods, Qualitative Data Collection pages 12-13, as well as the Supplementary Materials which includes both the

**No**	**Item**	**Guide questions/description**	**Responses and reference in manuscript if appropriate**
			Feedback Booklets and interview Schedules.
18.	Repeat interviews	Were repeat interviews carried out? If yes, how many?	No
19.	Audio/visual recording	Did the research use audio or visual recording to collect the data?	See Manuscript, Study Methods page 13
20.	Field notes	Were field notes made during and/or after the interview or focus group?	No
21.	Duration	What was the duration of the interviews or focus group?	See Manuscript, Study Methods, Interviews, page 13
22.	Data saturation	Was data saturation discussed?	Yes, see Study Protocol

No	Item	Guide questions/description	Responses and reference in manuscript if appropriate
23.	Transcripts returned	Were transcripts returned to participants for comment and/or correction?	No
**Domain 3: analysis and findings**			
Data analysis			
24.	Number of data coders	How many data coders coded the data?	See Manuscript, Data Analysis, pages 13-14
25.	Description of the coding tree	Did authors provide a description of the coding tree?	See Manuscript, Data Analysis, pages 13-14
26.	Derivation of themes	Were themes identified in advance or derived from the data?	See Manuscript, Data Analysis, pages 13-14

No	Item	Guide questions/description	Responses and reference in manuscript if appropriate
27.	Software	What software, if applicable, was used to manage the data?	See Manuscript, Data Analysis, pages 13-14
28.	Participant checking	Did participants provide feedback on the findings?	No
Reporting			
29.	Quotations presented	Were participant quotations presented to illustrate the themes / findings? Was each quotation identified? e*.g. participant number*	See Manuscript, Results Pages 15-26 and Case Studies, Box 1.
30.	Data and findings consistent	Was there consistency between the data presented and the findings?	See Manuscript, Results Pages 15-26 .
31.	Clarity of major themes	Were major themes clearly presented in the findings?	See Manuscript, Results Pages 15-26.

No	Item	Guide questions/description	Responses and reference in manuscript if appropriate
32.	Clarity of minor themes	Is there a description of diverse cases or discussion of minor themes?	See Manuscript, Results Pages 15-26.
