# Supplementary material for: Recovery from recurrent depression with mindfulness-based cognitive therapy and antidepressants: a qualitative study with illustrative case studies
Source: BMJ Open. 2020 Feb 18;10(2):e033892. doi: 10.1136/bmjopen-2019-033892 (PMC7044862; doi:10.1136/bmjopen-2019-033892)
Supplement: Supplementary data [file bmjopen-2019-033892supp003.pdf]

PREVENT

1-month Feedback Booklet

telephone: 01392 726102 email: prevent@exeter.ac.uk web: www.prevent-southwest.org.uk
ISRCTN26666654 EudraCT number: 2009-012428-10
In collaboration with The University of Bristol, King's College London and the Medical Research Council Cognition and Brain Sciences Unit. Funded by National Institute for Health Research HTA

PREVENT Feedback Booklet

As researchers, we are very aware that we are working with people. In this case, a specific group of people who have all suffered from depression at times in their lives. We are careful to never lose sight of the fact that each person is also an individual with their own personality, feelings, and experiences.

One of our aims is to find out about the thoughts, feelings and experiences of the people taking part in this research project at two time points: 4-6 weeks after your final session of MBCT, and again at the end of your involvement in the project. We want to hear your individual views because we think that we can learn a lot from them.

Please take time to think about the questions in this booklet and answer them in your own words. It might be useful to read through the questions before writing your answers, in order to become familiar with them. Tell us exactly what you think! We won't be offended if you didn't like some parts, or even all, of the project. If you found some or all of it useful, helpful or enjoyable, then tell us that too, and please tell us why.

There are 11 questions in the booklet. Write as much, or as little as you like. If you feel that you have more to say than there is space for then please call us on 01392 726102 and we'll provide you with a second booklet.

As we have said in the past, we guarantee your privacy and confidentiality. Nothing that you say here will be published or printed in any way that could identify you, and no unauthorised person will ever read this booklet.

Once you have completed the booklet, please return it to us in the envelope provided. The envelope has a freepost sticker on it, so you do not need to pay for postage.

Finally, many thanks for the effort you have put in to this project so far. Hopefully our research will enable us to develop new and better ways to help people stay well after depression.

The PREVENT Team

PREVENT Trial ID: __________

PREVENT Feedback Booklet 1.1 v2. 02.10.10

Tickell A, *et al. BMJ Open* 2020; 10:e033892. doi: 10.1136/bmjopen-2019-033892

Reducing your antidepressants

The first set of questions aims to explore your **thoughts** towards, and **experiences** of, taking and reducing your antidepressant medication.

1. Please describe your experiences of using antidepressants before taking part in the MBCT course. For example, did you feel that antidepressants were having a positive impact upon your day-to-day life and functioning? Did you experience any difficulties taking antidepressants in the past? Please explain why.

....
....
....
....
....
....

2. During the MBCT course you were asked to try to reduce your use of antidepressants. Please can you describe what your **thoughts** were towards reducing your antidepressants directly before the MBCT course started and again during the MBCT course when you were asked to try reducing them?

Thoughts about reducing antidepressants *before* the MBCT course:

....
....
....
....
....
....

Thoughts about reducing antidepressants *during* the MBCT course:

....
....
....
....
....
....

Tickell A, *et al. BMJ Open* 2020; 10:e033892. doi: 10.1136/bmjopen-2019-033892

2. Can you please describe your **experiences** of reducing your use of antidepressants during the MBCT course up until this point now?
For example, have you started to reduce your antidepressants? (If YES, please continue below. If NO, please go to Question 3)

When did you start reducing your antidepressants? (Please be as specific as possible about the date)

....

What positive impact, if any, has reducing your antidepressants had on your day-to-day life and functioning?

....
....
....
....

What difficulties, if any, did you experience when reducing your antidepressants?

....
....
....
....
....
....

Did you do anything in particular to overcome these difficulties?

....
....
....
....
....
....

Tickell A, *et al. BMJ Open* 2020; 10:e033892. doi: 10.1136/bmjopen-2019-033892

If you have tried reducing your antidepressants in the past, please describe if your experiences of reducing your antidepressants recently has been different, and if so, in what ways. If there has been no difference, please let us know.

....
....
....
....
....
....

3. It would be useful for us to know more about why people who take part in this research study do not reduce their antidepressants. Please could you describe why you have not reduced your antidepressant medication at this stage in the research study?

....
....
....
....
....
....
....
....

Tickell A, *et al. BMJ Open* 2020; 10:e033892. doi: 10.1136/bmjopen-2019-033892

Taking part in MBCT

The questions in this section aim to find out more about your experiences of taking part in MBCT, and continuing the MBCT exercises after the course.

4. Out of the 8 week course, how many MBCT sessions did you attend?

.... out of 8 sessions.

5. Did you attend the first MBCT follow-up session? YES / NO

6. What, if any, MBCT exercises are you still practicing? Please tick the ones you are still practicing and in the space provided write how often you practice (e.g. per week or per month).

- None
- Body scan
  ....
- Sitting meditation
  ....
- 10-min sit
  ....
- 20-min sit
  ....
- Silence with bells
  ....
- Breathing space (regular, 3 times a day)
  ....
- Breathing space (‘coping’ space)
  ....
- Mindful walking
  ....
- Stretch and breath
  ....
- Mindful activities (please specify what, and how often per week or month)
  ....
- Other (please specify)
  ....

....

Tickell A, *et al. BMJ Open* 2020; 10:e033892. doi: 10.1136/bmjopen-2019-033892

7. Please describe anything that you may have found **helpful** about taking part in the MBCT course and practicing the mindfulness meditation exercises, **and why**.

Specific aspects of the MBCT course you found **helpful**, and why:

....
....
....
....
....
....
....
....
....

Specific aspects of the meditation practices and teachings that you found **helpful**, and why:

....
....
....
....
....
....
....
....
....

Tickell A, *et al. BMJ Open* 2020; 10:e033892. doi: 10.1136/bmjopen-2019-033892

8. Please describe anything that you may have found **unhelpful** about taking part in the MBCT course and practicing the mindfulness meditation exercises, **and why.**

Specific aspects of the MBCT course that you found **unhelpful**, and why:

....
....
....
....
....
....
....
....
....

Specific aspects of the MBCT practices and teachings that you found **unhelpful**, and why:

....
....
....
....
....
....
....
....
....

Tickell A, *et al. BMJ Open* 2020; 10:e033892. doi: 10.1136/bmjopen-2019-033892

The Impact of MBCT

The final two questions relate to the **impact**, if any, that MBCT may have had on you.

9. Have you benefitted from the MBCT course and practicing the MBCT exercises? If so, in what ways? (We are interested to hear about benefits not only in how you feel, but in any other areas of your life). If there have been no benefits or that some things are worse now, please tell us about these.

....

....

....

....

....

....

....

....

....

....

....

....

....

10. Do you feel that MBCT **works for you**? If so, why do you think it works? If it doesn’t work for you, please tell us why you think it doesn't work.

....

....

....

....

....

....

....

....

....

....

....

Tickell A, *et al. BMJ Open* 2020; 10:e033892. doi: 10.1136/bmjopen-2019-033892

Any other comments:

11. Do you have any other comments that you would like to share about your experiences of taking part in this project?

....
....
....
....
....
....
....
....
....
....
....
....
....
....

Tickell A, *et al. BMJ Open* 2020; 10:e033892. doi: 10.1136/bmjopen-2019-033892
