# Supplementary material for: Recovery from recurrent depression with mindfulness-based cognitive therapy and antidepressants: a qualitative study with illustrative case studies
Source: BMJ Open. 2020 Feb 18;10(2):e033892. doi: 10.1136/bmjopen-2019-033892 (PMC7044862; doi:10.1136/bmjopen-2019-033892)
Supplement: Supplementary data [file bmjopen-2019-033892supp004.pdf]

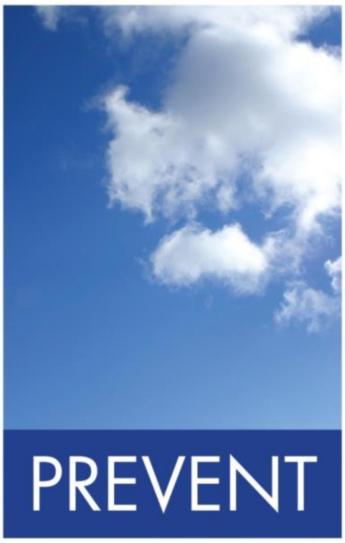

24-month Feedback Booklet

telephone: 01392 726102 email: prevent@exeter.ac.uk web: www.prevent-southwest.org.uk
ISRCTN26666654 EudraCT number: 2009-012428-10
In collaboration with The University of Bristol, King's College London and the Medical Research Council Cognition and Brain Sciences Unit. Funded by National Institute for Health Research HTA

PREVENT 24-month Feedback Booklet

As researchers, we are very aware that we are working with people. In this case, a specific group of people who have all suffered from depression at times in their lives. We are careful to never lose sight of the fact that each person is also an individual with their own personality, feelings, and experiences.

One of our aims is to find out about the thoughts, feelings and experiences of the people taking part in this research project. We asked you to complete a feedback booklet in the weeks after attending MBCT, and we would be really grateful if you could now complete a second feedback booklet to explore your experiences of taking part in the project over the past two years. We want to hear your individual views because we think that we can learn a lot from them.

Please take time to think about the questions in this booklet and answer them in your own words. It might be useful to read through the questions before writing your answers, in order to become familiar with them. Tell us exactly what you think! We won't be offended if you didn't like some parts, or even all, of the project. If you found some or all of it useful, helpful or enjoyable, then tell us that too, and please tell us why.

There are 4 parts to this booklet and 27 questions in total, although not all of them will apply to you. Please read the instructions throughout the booklet to check which questions are relevant to you.

Please write as much, or as little, as you like. If you feel that you have more to say than there is space for then please use the back sheet to write any additional feedback.

As we have said in the past, we guarantee your privacy and confidentiality. Nothing that you say here will be published or printed in any way that could identify you, and no unauthorised person will ever read this booklet.

Once you have completed the booklet please give it to your PREVENT researcher at the 24-month assessment.

Finally, many thanks for the effort you have put in to this project. Hopefully our research will enable us to develop new and better ways to help people stay well after depression.

The PREVENT Team

PREVENT Trial ID: _________

Tickell A, *et al. BMJ Open* 2020; 10:e033892. doi: 10.1136/bmjopen-2019-033892

Part I: Experiences of Reunions

Following your MBCT course you were invited to attend four MBCT reunion sessions.

1. How many did you attend? (Please circle)

None 1 2 3 4

2. Were there any specific reasons for attending this number of reunion sessions?

....

....

....

....

....

....

....

If you didn't attend any reunions, please go to Part II on page 4.

If you attended some or all reunion sessions, please continue below.

3. Please describe what you found helpful and / or unhelpful about the reunion sessions?

....

....

....

....

....

....

....

4. Have you used any other support to maintain your mindfulness practice (for example, keeping in contact with anyone from your MBCT course, or joining local meditation groups)? Please describe how this has been helpful and / or unhelpful and why.

....

....

....

....

....

....

....

Part II: Doing Mindfulness Practices

Which of the following statements best describes your use of mindfulness techniques (both formal practices and other mindful activities) over the past two years? Please tick one box and then follow the instructions for which question to complete next:

☐ I've used mindfulness techniques regularly over the last 2 years (please continue to Question 5)

☐ I used mindfulness techniques for a while after the MBCT course but my practice has tailed off and I no longer practice (please continue to Question 5)

☐ I've used mindfulness techniques off and on during the last 2 years (please continue to Question 5)

☐ I have not used mindfulness techniques at all since the MBCT course (please go to Question 7)

5. Please complete the two tables below to tell us which techniques you have practiced over the past two years and what you are currently practicing.

The following table provides a list of mindfulness practices from the MBCT course.

	Currently	Over the past two years
**Example: Body Scan**	2 times per week	Regularly for about 3 months after the course but then stopped. Restarted 2 months ago.
**Body Scan**	times per	
**Sitting Meditation**	times per	
**10-minute sit**	times per	
**20-minute sit**	times per	
**Silence with Bells**	times per	
**Breathing Space (Regular, e.g. 3 times a day)**	times per	
**Breathing Space ('Coping' space)**	times per	
**Mindful Walking**	times per	
**Mindful Movement / Yoga**	times per	

The following table gives you space to record any mindful activities you've practiced.

Mindful Activities	Currently	Over the past two years
E.g., being mindful when showering	*Every morning*	*Every morning, although I stopped for roughly 3 months about a year ago.*

6. Have you adapted any of the mindfulness exercises you learned in the MBCT course to suit you? If so, how? Why did you make these changes?

....

....

....

....

....

....

....

7. If you do not currently practice any mindfulness techniques, please describe why.

....

....

....

....

....

....

Part III: The impact of MBCT and mindfulness practice

8. Do you feel that MBCT "works" for you? If so, how do you think it works? If you would like to, please use examples from your life.

....
....
....
....
....
....
....

9. What is the single most important thing that you've learned through attending the MBCT course or practicing mindfulness techniques? Please explain your answer.

....
....
....
....
....
....
....

10. MBCT may not be the right treatment for everyone. If you feel that MBCT was not right for you, please tell us why.

....
....
....
....
....
....
....

11. What changes, if any, would you make to the MBCT course and / or mindfulness exercises to make them more suitable for you?

....

....

....

....

....

....

....

....

....

Part IV: Taking antidepressants

We are interested in learning more about your experiences of taking and reducing antidepressants over the past two years.

12. Have your thoughts about reducing your antidepressants changed over the past two years? If so, in what ways?

....

....

....

....

....

....

13. Does taking antidepressant medication “work” for you? If so, please tell us why. If taking antidepressants does not “work” for you, please also tell us why. If you would like to, please use examples from your life.

....

....

....

....

....

....

14. What role has your GP had in your use of antidepressant medication over the past two years? How have you found this?

....

....

....

....

....

....

15. Has anyone else been involved in your use of antidepressant medication over the past two years? (E.g., psychiatrist, family member, another practitioner). If so, in what ways?

....

....

....

....

....

....

Which of the following best describes your use of antidepressants over the last two years? Please tick one box and follow the instructions for which questions to complete next.

☐ I have continued using antidepressants and have not reduced my use at any point over the past two years that I've been involved in this study. (Tick this box even if you have changed to a different antidepressant, or increased the dosage of your antidepressant.)

Please answer the questions in the ◆ box on the following page.

☐ I am in the process of reducing my antidepressants, or have reduced/stopped my use of antidepressants at some point over the past two years that I've been involved in this study. (Tick this box even if you restarted or increased your medication at a later date.)

Please answer the questions in the ● box on page 12

16. In your day-to-day life and functioning over the past two years, have you experienced any positive effects of continuing to use antidepressants? If so, please describe these.

17. In your day-to-day life and functioning over the past two years, have you experienced any negative effects or difficulties in continuing to use antidepressants? If so, please describe these.

18. Have you done anything specific to overcome these negative effects or difficulties? If so, what have you done?

19. Please describe why you haven't reduced your antidepressant medication over the past two years.

Please now go to Question 27 on page 11.

20. Has reducing your use of antidepressants had any positive effects on your day-to-day life and functioning over the past two years? If so, please describe how.

21. Have you experienced any negative effects or difficulties associated with reducing your antidepressants over the past two years? If so, please describe them.

22. Have you done anything specific to overcome these negative effects or difficulties? If so, what have you done?

23. Has practicing mindfulness techniques helped with these negative effects or difficulties? If so, in what ways?

Continued...

Continued...

24. Have you stopped using antidepressant medication completely, now or at any point in the last two years? Please describe your experiences of this.

25. Have you tried reducing your antidepressants at any point before this study? If so, please tell us of any ways that reducing your antidepressants over the past two years been different? If there has been no difference, why do you think this is the case?

26. Have you reduced or stopped your antidepressants over the past two years and then restarted or increased them again? If so, please tell us about this, and how you felt about this?

Please go to Question 27 on the following page.

Any other comments?

27. **Thank you for taking the time to complete this booklet - your participation is greatly appreciated!** If you have any other comments that you would like to share about your experiences of taking part in this project which did not fit into any of the answer spaces above, please use the space below.
