# Supplementary material for: Recovery from recurrent depression with mindfulness-based cognitive therapy and antidepressants: a qualitative study with illustrative case studies
Source: BMJ Open. 2020 Feb 18;10(2):e033892. doi: 10.1136/bmjopen-2019-033892 (PMC7044862; doi:10.1136/bmjopen-2019-033892)
Supplement: Supplementary data [file bmjopen-2019-033892supp005.pdf]

PREVENT: End of Trial Interviews
Guidance for Interviewers

Aims

Researchers will conduct interviews with 42 purposively sampled participants in the MBCT arm of the trial at the end of the trial. The research topic we are targeting here is:
Participants' experiences during the follow-up period of the PREVENT trial of use of ADMs and MBCT-based techniques in relation to each other, during periods of wellness, depressive relapse, and transitions between these two.

Interview Preparation

Before conducting any of the interviews researchers read the key qualitative papers (Malpass et al., 2012; Allen et al., 2009). Before each interview researcher to read the participant's file to (re-) familiarise themselves with participant characteristics (e.g., relationship status) and obtain timeline populated with info on borderline or actual relapses and ADM usage. They should also review, and have to hand in the interview, the end of treatment and 24-month feedback booklets to get a sense of the profile of each participant during the follow-up period in terms of the following variables: full or partial attendance of MBCT course; use of mindfulness techniques; use of ADMs; and any significant life events. This will shape the interview in terms of: i) which sub-sections of the interview are relevant; ii) issues for particular participants that may need to be probed in more detail.
Make sure you are as clear as possible about which sections of the interview and questions are relevant to your respondent BEFORE YOU START. Where possible, **choose specific/representative/salient episodes or junctures** in timeline for targeted probing using the schedule (possibly match them onto wellness, wobbles, and depressive sections). **Print out two copies of timeline**, one for yourself and one for the interviewee to draw upon.

Conducting the Interview

The interview is semi-structured. In each section, questions and follow-up questions are suggested. However, researchers should use their judgment in drawing out participants in relation to the research topic. Interviewers ask open-ended questions and follow participants' "leads" while keeping in mind the research question. The aim is to enable participants to give their "story" around the main topics of investigation.
Researchers should be strategic in use of time in the interview to ensure the topic guide/respondent covers material that answers the research questions; especially in Section 1, it is important to keep the interview focused. If the interviewer feels respondents are being unclear or opaque help the respondent unpack what they mean or agree with the participant that the issue is unclear. Interviewers should ensure *that all the interview questions that are relevant to the participants' particular profile are covered.* Consider a temporal order to do the interview allocating/planning pre-set time windows for each consecutive episode/junction so as not to exceed a **maximum of 60 minutes** for the overall interview.

Participants may feel the need to please researchers or give the "right" answers. This is particularly likely around ADM tapering where the trial has consistently communicated that we want participants to taper and discontinue their ADM. It is important that researchers communicate explicitly and non-

Tickell A, *et al. BMJ Open* 2020; 10:e033892. doi: 10.1136/bmjopen-2019-033892

verbally that there are no right and wrong answers to any of the questions and we want to know people's experiences in relation to MBCT and ADM.
Use of particular terms (e.g., "wobble," "relapse") can be adapted in the light of language used by participants. Follow-up questions should be asked for clarification and elaboration, *and this should be driven by the research aim for each section of the interview*. As far as possible interviewers should cover all the suggested topics and questions and behave with the minimum of variation between interviews. Instructions to interviewers and suggested wordings for introductions to each section are given in italics below. The key issues for each question are in bold.

Interview Opening / Introduction

Open the interview with something like:

"Thank you for agreeing to do this interview with me. The interview is about hearing from you how things have been in the trial and checking that we have your story right. It will be collaborative and semi-structured which means I have some topics I'd like to cover, but the questions will be quite open so as to give you, as an expert of your own lived experience, a chance to express your own views and tell me about your experiences and what seems important to you. Everyone will have had different experiences and we are interested in hearing about these from your point of view – both the good and the not-so-good experiences – so that we can learn about and develop our treatments further. Please tell us exactly how you feel!
Just to let you know that I am un-blinded and know that you have participated in the MBCT group, so it is ok to talk freely about this. I would like to record this interview, and the recording will be transcribed word for word and analysed as part of the research for the PREVENT trial. All identifying information will be removed at this point. Your name will never appear on the transcription or any other documents or files that result from this interview (such as the audio file). Do you have any questions before we begin?"

Section 1: Overview of the follow-up period using the timeline

The aim of this section is to obtain a brief overview of how the participant has experienced the follow-up period in terms of three areas: 1. Periods of wellness and depression; 2. Antidepressant medication; 3. Life events. Subsequent interview sections follow up on each of these in more detail and the profile of experiences here can be used to tailor subsequent interview questions. Information on the first two areas should be available in advance and summarised on the prepared timeline. Information on life events might be found in the research files and database but will need to be obtained here. Preparation by thoroughly reading the file, and possibly even some of the audio recordings, in advance will help a lot here.
Use the timeline in whatever way feels most comfortable and guided by the respondent's preference. The participant can add life events him/herself; or the researcher can do this; or it can simply be used as a guide. Show the timeline and say something like:

"I'd like to start with a brief overview of how life has been for you in the trial. In preparing for our meeting today, we have put together this timeline. It summarises the information you have given us as part of the research study about any periods of depression or wobbles, and your use of antidepressant medication during the trial. It also includes the date of your most recent episode of depression before taking part in the trial, based upon what you told us during your first assessment. I've also

Tickell A, *et al. BMJ Open* 2020; 10:e033892. doi: 10.1136/bmjopen-2019-033892

looked through your comments in the Feedback Booklets you completed one month after the end of the mindfulness course and again recently. These were very helpful and I'll use some of what you wrote there to guide what we talk about today."

Interview Question	Probes/Examples/Directions
1.1. Do you think the information we have here on the timeline looks about right? Is there anything you would like to change?	If we know from previous assessments that the person has had a significant life event ensure this is acknowledged here. Mark – or get the respondent to mark – any life events on the time-line / graph.
1.2. Have there been any important events in your life during your time in the trial that have affected you either positively or negatively that we can add to the timeline?	Keep the discussion focused and brief to allow as much time for later sections as possible.
1.3. Can we check that I have your use of antidepressant medication right these last two years?	Work through timeline for any tapering/discontinuation, dosage, resumption etc.
1.4. How have things been between the last time we spoke and today?	Informally extend timeline with relevant depressive episodes/ADM use/life events up until current interview date; Where necessary, establish current symptomatic status (informally) and make sure they are ok to continue.

"The remaining questions in this interview will focus upon your experiences with the aid of this timeline."

Keep the time-line / graph in view to refer back to / use further in later parts of the interview. Use the timeline to keep the interview contained with regard to the different episodes/junctures identified prior to interview. Allow and encourage the participant to use a pen to put down details in their copy of the timeline.

Section 2: Questions on wellness (for all)

Comment on Section 2-4: Particularly in the wobbles section it is important to go for depth rather than breadth with regards to potential issues around these time points (e.g. what happened in days before, relationships, sleep etc.). As a general principle, interviewers should encourage interviewees to focus on prototypical or most memorable junctures in timeline. In order to keep interview contained, agree on a timeframe for each episode/juncture and keep questions focused on this.

"I'd like to ask about any times when you were well during the trial – when you weren't feeling low or experiencing an episode of depression"

Tickell A, *et al. BMJ Open* 2020; 10:e033892. doi: 10.1136/bmjopen-2019-033892

Interview Question	Probes/Examples/Directions
2.1. Has anything from the mindfulness course played a part in staying well during the trial? If so, can you describe how?	Examples: any techniques, ideas; response plan<br>Prompts: How / why / why not?; Therapist/Group/Researcher Role
2.2 Did your use of antidepressants play a part in staying well during the trial? If so, can you describe how?	Prompts: How / why / why not? ; GP Role
2.3 What about the combination of xxx from the mindfulness course and use of antidepressants in periods of wellness?	[ask this additional question if respondent says that both MBCT and ADM have had some value in questions 2.2 and 2.3., otherwise skip]

Section 3: Question on wobbles / early signs of depression (for all)

"I'd like to ask about any times when you were well, but you felt yourself starting to 'wobble' or feel low. So any times when you might have felt that your mood was dipping or you were starting to have more of the negative thoughts that were around when you were depressed"

Interview Question	Probes/Examples/Directions
3.1. How did these experiences of 'wobbles' or starting to feel low compare to previous experiences of wobbles?	Use timeline to go right into the situation shortly before, during and directly after episode.<br>Probe: What happened, how was it different? What did you do? How did you get out of it?
3.2. Was anything from the mindfulness course useful at the time of wobbling or when starting to feel low during the trial? If so, can you describe how?	Probe: before/during/after wobble<br>Examples: any techniques, ideas; response plan, self-compassion etc.<br>Prompts: How / why / why not?
3.3 Did your use of antidepressants during the trial play a part in wobbling or starting to feel low during the trial? If so, can you describe how?	Prompts: How / why / why not?
3.4 What about the combination of xxx from the mindfulness course and use of antidepressants in periods of wobbling or when starting to feel low?	[ask this additional question if respondent says that both MBCT and ADM have had some value, otherwise skip as appropriate]

Tickell A, *et al. BMJ Open* 2020; 10:e033892. doi: 10.1136/bmjopen-2019-033892

Section 4: Experiences of depressive relapse (for those who have relapsed)

Use the timeline to guide question choices in relation to experiences of relapse.

Say: "I'd like to ask you about your experiences this / these episode of depression..."

Interview Question	Probes/Examples/Directions
4.1. How did this episode of depression compare to previous episodes of depression?	Use timeline to take them right into the situation shortly before, during and directly after episode. Probe: What happened, how was it different? What did you do? How did you get out of it?
4.2. Was anything from the mindfulness course useful at the time of depression? If so, can you describe how?	Examples: any techniques, ideas; response plan<br>Prompts: How / why / why not?
4.3. Did your use of antidepressants during the trial play a part in this episode of depression? If so, can you describe how?	Prompts: How / why / why not?
4.4 What about the combination of xxx from the mindfulness course and use of antidepressants in periods of depression?	[ask this additional question if respondent says that both MBCT and ADM have had some value, otherwise skip as appropriate]

Section 5: MBCT and ADMs combined

"I'd like to end with some open questions about your experiences of taking part in this study."

For sakes of time keeping and for keeping this section contained it is critical how the questions are set up. Encourage people to step back from and reflect critically on their own experience rather than letting them share their raw experience. Use formulations like the following: *'I am sure that this was an intensive time for you. If you took yourself away from the experience today and were to reflect on this, what may I ask are the key elements/thoughts that you would have with regards to staying well etc.?'*

Interview Question	Probes/Examples/Directions
5.1. Has taking part in the trial changed the way you think about depression? If so, could you tell me how?	Probe: How/why /why not?<br>Probe: Has it changed the way you think about the causes/consequences of depression? Role of GP.
5.2. Has taking part in the trial changed how you think about yourself? If so, could you tell me how?	Probe: how identity was before the trial if person describes a sense of change.<br>Examples: Role of group, immediate and wider social

Tickell A, *et al. BMJ Open* 2020; 10:e033892. doi: 10.1136/bmjopen-2019-033892

	environment
5.3. Now that you have had experiences of both mindfulness and antidepressants, what do you think of each of them as treatments for depression?	Probes: do you favour one over the other? In combination?<br>At different points e.g. in wellness, wobbles and depressive episodes?<br>For prevention? For recovery?
5.4. Apart from depression, has taking part in the trial had any impact on other psychological or physical health problems you may have?	Examples:<br>Anxieties/phobias, Chronic health conditions like pain, diabetes etc.

Section 6: Ending

"I've covered all the questions we have, but before we end, is there anything you would like to add?"

Interview Question	Probes/Examples/Directions
6.1. …about any of the topics we have discussed?	
6.2. …about any of your experiences of depression, mindfulness or antidepressant medication that we have not talked about?	
6.3. …about anything else you think is relevant to this project on MBCT, antidepressants, and depression?	

End by thanking the respondent for their time and for sharing their views and experiences. Reiterate how valuable this is for research trying to develop treatments for depression. Inform them about further dissemination of PREVENT findings.

Tickell A, *et al. BMJ Open* 2020; 10:e033892. doi: 10.1136/bmjopen-2019-033892
