# Supplementary material for: Recovery from recurrent depression with mindfulness-based cognitive therapy and antidepressants: a qualitative study with illustrative case studies
Source: BMJ Open. 2020 Feb 18;10(2):e033892. doi: 10.1136/bmjopen-2019-033892 (PMC7044862; doi:10.1136/bmjopen-2019-033892)
Supplement: Author's manuscript [file bmjopen-2019-033892.draft_revisions.pdf]

BMJ Open

BMJ Open is committed to open peer review. As part of this commitment we make the peer review history of every article we publish publicly available.

When an article is published we post the peer reviewers' comments and the authors' responses online. We also post the versions of the paper that were used during peer review. These are the versions that the peer review comments apply to.

The versions of the paper that follow are the versions that were submitted during the peer review process. They are not the versions of record or the final published versions. They should not be cited or distributed as the published version of this manuscript.

BMJ Open is an open access journal and the full, final, typeset and author-corrected version of record of the manuscript is available on our site with no access controls, subscription charges or pay-per-view fees (http://bmjopen.bmj.com).

If you have any questions on BMJ Open's open peer review process please email info.bmjopen@bmj.com

BMJ Open

Discontinuing antidepressants with the support of psychological therapy: a qualitative study in the UK.

Journal:	*BMJ Open*
Manuscript ID	bmjopen-2019-033892
Article Type:	Research
Date Submitted by the Author:	27-Aug-2019
Complete List of Authors:	Tickell, Alice; University of Oxford, Psychiatry<br>Byng, Richard; University of Plymouth, Community and Primary Care Research Group<br>Crane, Catherine; University of Oxford, Psychiatry<br>Gradinger, Felix; Plymouth University, Community and Primary Care Research Group<br>Hayes, Rachel; University of Exeter, College of Medicine and Health<br>Robson, James; University of Oxford, Education<br>Cardy, Jessica; Oxford University, Oxford Institute of Clinical Psychology Training<br>Weaver, Alice; Devon Partnership NHS Trust<br>Morant, Nicola; University College London, Psychiatry<br>Kuyken, Willem; University of Oxford, Psychiatry
Keywords:	Depression & mood disorders < PSYCHIATRY, psychological therapy, antidepressants

SCHOLARONE™
Manuscripts

I, the Submitting Author has the right to grant and does grant on behalf of all authors of the Work (as defined in the below author licence), an exclusive licence and/or a non-exclusive licence for contributions from authors who are: i) UK Crown employees; ii) where BMJ has agreed a CC-BY licence shall apply, and/or iii) in accordance with the terms applicable for US Federal Government officers or employees acting as part of their official duties; on a worldwide, perpetual, irrevocable, royalty-free basis to BMJ Publishing Group Ltd ("BMJ") its licensees and where the relevant Journal is co-owned by BMJ to the co-owners of the Journal, to publish the Work in this journal and any other BMJ products and to exploit all rights, as set out in our licence.

The Submitting Author accepts and understands that any supply made under these terms is made by BMJ to the Submitting Author unless you are acting as an employee on behalf of your employer or a postgraduate student of an affiliated institution which is paying any applicable article publishing charge ("APC") for Open Access articles. Where the Submitting Author wishes to make the Work available on an Open Access basis (and intends to pay the relevant APC), the terms of reuse of such Open Access shall be governed by a Creative Commons licence – details of these licences and which Creative Commons licence will apply to this Work are set out in our licence referred to above.

Other than as permitted in any relevant BMJ Author's Self Archiving Policies, I confirm this Work has not been accepted for publication elsewhere, is not being considered for publication elsewhere and does not duplicate material already published. I confirm all authors consent to publication of this Work and authorise the granting of this licence.

For peer review only

DISCONTINUING ANTIDEPRESSANTS WITH THE SUPPORT OF PSYCHOLOGICAL THERAPY: A QUALITATIVE STUDY IN THE UK

Alice Tickell, Department of Psychiatry, University of Oxford, alice.tickell@psych.ox.ac.uk

Richard Byng, Community and Primary Care Research Group, University of Plymouth, richard.byng@plymouth.ac.uk

Catherine Crane, Department of Psychiatry, University of Oxford, catherine.crane@psych.ox.ac.uk

Felix Gradinger, Community and Primary Care Research Group, University of Plymouth, felix.gradinger@plymouth.ac.uk

Rachel Hayes, College of Medicine and Health, University of Exeter, r.a.hayes@exeter.ac.uk

James Robson, Department of Education, University of Oxford, james.robson@education.ox.ac.uk

Jessica Cardy, Oxford Institute of Clinical Psychology Training, University of Oxford, jessica.cardy@hmc.ox.ac.uk

Alice Weaver, Devon Partnership NHS Trust, a.weaver@exeter.ac.uk

Nicola Morant, Division of Psychiatry, University College London, n.morant@ucl.ac.uk

Willem Kuyken, Department of Psychiatry, University of Oxford, willem.kuyken@psych.ox.ac.uk

Correspondence concerning the article should be addressed to Professor Willem Kuyken, Department of Psychiatry, Warneford Hospital, Oxford, OX3 7JX.

Email: willem.kuyken@psych.ox.ac.uk

Word Count

9881 (excluding title page, abstract, references, figures, and tables)

For peer review only

Contributor Statement

WK, RB & NM were responsible for the PREVENT trial protocol and secured the study funding. NM designed the over-arching qualitative process study to elicit service users' experiences of treatment, with input from RB, FG, RH, JC, and WK. Interviews were conducted by FG and AW, supervised by NM. CC,WK, JR, and AT developed the analytical strategy and protocol for the study reported here, and AT conducted the bulk of the analysis, with input from other members of the analytical team. AT drafted the manuscript with input from CC. All other authors read the manuscript, revised it for significant intellectual content, and approved the final manuscript. As Chief Investigator, WK had overall responsibility for the parent trial within which this study was embedded. The University of Exeter held responsibility for the parent trial and this work. WK is guarantor and corresponding author for the study.

Competing Interest Statement

All authors have completed the ICMJE uniform disclosure form at www.icmje.org/coi_disclosure.pdf and declare the following: AT reports that she is employed as a researcher at the University of Oxford Mindfulness Centre, conducting research on mindfulness-based interventions. However, she receives no additional remuneration for teaching or publications related to mindfulness-based approaches. RB has written about his concerns about the increases in prescribing of antidepressants. CC reports that she is employed as a researcher at the University of Oxford Mindfulness Centre, conducting research on mindfulness-based interventions. However, she receives no additional remuneration for teaching or publications related to mindfulness-based approaches. WK is the Director of the Oxford Mindfulness Centre. He receives no payments for training workshops and presentations related to MBCT; such payments are made directly to the Oxford Mindfulness Foundation, a charitable trust that supports the work of the Oxford Mindfulness Centre. He receives royalties for his book Mindfulness: Ancient wisdom meets modern psychology published by Guilford Press. WK was until 2015 an unpaid Director of the Mindfulness Network Community Interest Company and gave evidence to the UK Mindfulness All Party Parliamentary Group. FG, RH, JR, JC, AW, and NM have nothing to disclose.

For peer review only - http://bmjopen.bmj.com/site/about/guidelines.xhtml

Transparency Statement

WK confirms that the manuscript is an honest, accurate, and transparent account of the study being reported; that no important aspects of the study have been omitted; and that any discrepancies from the study as originally planned have been explained. He attests that all listed authors meet authorship criteria and that no others meeting the criteria have been omitted.

Funding Statement

The PREVENT trial was funded by the National Institute for Health Research Health Technology Assessment (NIHR HTA) Programme [08/56/01] and was published in full in Health Technology Assessment. The views and opinions expressed therein are those of the authors and do not necessarily reflect those of the HTA programme, NIHR, NHS or the Department of Health and Social Care. A Trial Steering Committee (TSC), Data and Ethics Monitoring Committee (DMEC), the UK Mental Health Research Network (MHRN), the Primary Care Research Network (PCRN), and the Comprehensive Local Research Network (CLRN) all provided support to the project. WK, CC and AT are supported by the Wellcome Trust [107496/Z/15/Z]. RB received funding from NIHR Collaboration for Leadership in Applied Health Research and Care South West Peninsula.

The funders had no role in the design of the study, in the collection, analysis and interpretation of the data, in the writing of the report, or in the decision to submit the article for publication. All authors are independent of the funders, had full access to all of the data in the study, and can take responsibility for the integrity of the data and the accuracy of the data analysis.

License

The Corresponding Author has the right to grant on behalf of all authors and does grant on behalf of all authors, a worldwide licence to the Publishers and its licensees in perpetuity, in all forms, formats and media (whether known now or created in the future), to i) publish, reproduce, distribute, display and store the Contribution, ii) translate the Contribution into other languages, create adaptations, reprints, include within collections and create summaries, extracts and/or, abstracts of the Contribution and convert or allow conversion into any format including without limitation audio, iii)

For peer review only - http://bmjopen.bmj.com/site/about/guidelines.xhtml

create any other derivative work(s) based in whole or part on the on the Contribution, iv) to exploit all subsidiary rights to exploit all subsidiary rights that currently exist or as may exist in the future in the Contribution, v) the inclusion of electronic links from the Contribution to third party material where-ever it may be located; and, vi) licence any third party to do any or all of the above. All research articles will be made available on an open access basis. The terms of such open access shall be governed by a Creative Commons licence—details as to which Creative Commons licence will apply to the research article are set out in our worldwide licence referred to above.

Acknowledgements

We would like to thank Trish Bartley for her input to MBCT therapist training and MBCT fidelity checks. We are grateful to members of our Trial Steering Committee (Chris Leach, Richard Moore and Glenys Parry) and Data Monitoring Committee (Paul Ewings, Andy Field and Joanne MacKenzie) for their valuable advice and support during the project. We acknowledge the additional support provided by the Mental Health and Primary Care Research Networks. We also acknowledge the support provided by the Department of Health and local Primary Care Trusts, in meeting the excess treatment and service support costs associated with the trial. Thanks go to the PREVENT research team, who facilitated wider qualitative work in the trial, including Aaron Causley, Anna Hunt, Pooja Shah, Holly Sugg, Harry Sutton, and Matthew Williams. Above all, we are grateful to the participants for their time in taking part in this trial.

Data Sharing Statement

We will not be making the data publicly available due to its highly confidential and identifiable nature.

Dissemination Declaration

The trial results were disseminated in workshops and via a flyer to all participants who requested this feedback. The findings of this study will be disseminated to relevant audiences through University of Oxford communications.

For peer review only

ABSTRACT

Objectives: The aim of this study was to describe and interpret people's experiences in the two years after taking part in a psychological programme to support discontinuation of maintenance antidepressant medication (ADM) and recovery from recurrent depression.

Design: A qualitative study embedded within a multicentre, single blind, randomised controlled trial (the PREVENT trial).

Setting: The trial recruited participants from general practices (GP) in urban and rural settings in four UK centres. Qualitative data was gathered from interviews conducted in people's homes.

Participants: 42 people who participated in the active arm of the trial and were purposively sampled to represent a range of treatment responses and ADM discontinuation profiles.

Intervention: Mindfulness-based cognitive therapy with tapering support to facilitate discontinuation of maintenance ADMs (MBCT-TS). Eight weekly group sessions, with four refresher sessions offered in the year following the end of the programme compared to continuation of maintenance ADMs.

Main outcomes: In-depth interviews and written feedback collected in the two years after MBCT-TS.

Results: Many participants reported beneficial effects of learning MBCT-TS to support relapse prevention, which facilitated tapering or full discontinuation of ADMs. There were also aspects of people's experiences that contributed to their decisions to remain on ADMs. The analysis yielded seven over-arching themes: (1) beliefs about the causes of depression; (2) control; (3) acceptance; (4) quality of life; (5) tapering process; (6) interactions with GP, and (7) timing.

Conclusions: Psychological therapy can support people with a history of recurrent depression to discontinue ADMs, by teaching skills to manage depressive symptoms and the tapering process. However, this is an effortful process, requiring time and motivation to learn and apply psychological techniques, relatively stable life circumstances, and adequate support from medical professionals. Nevertheless, psychological therapy may increase quality of life whether or not the person successfully discontinues their ADMs or experiences further depressive symptoms.

For peer review only - http://bmjopen.bmj.com/site/about/guidelines.xhtml

STRENGTHS AND LIMITATIONS OF THE STUDY

- This qualitative study explored experiences of participants with recurrent depression attempting to taper and discontinue maintenance antidepressant medication with the support of psychological therapy.
- Participants were followed up for two years following participation in psychological therapy, and the study represents one of the first to provide a longer-term perspective on psychological therapy-supported antidepressant discontinuation.
- The study raises questions about the contributions of both psychological therapy and antidepressant medication to recovery from recurrent major depression, and how such recovery is measured.
- Participants were drawn from a large, pragmatic, randomised controlled trial which recruited people open to trying psychological therapy as an alternative to maintenance antidepressants. Thus participants are unlikely to be fully representative of people with recurrent depression seen in primary care.
- We interviewed participants who had attended at least 50% of psychological therapy sessions offered. Thus findings do not reflect the experiences of those people who were randomised to, but did not choose to engage with psychological therapy.

For peer review only

Depression is a major public health problem: globally more than 300 million people suffer from depression, and lifetime prevalence rates are estimated to be between 6-20% across both high and low-middle income countries.[1,2] Furthermore, depression is a relapsing and recurring condition, and on average people have seven or eight depressive episodes over their lifetime.[3] Current guidance from the National Institute for Health and Clinical Excellence (NICE) recommends that people with recurrent depression should be prescribed maintenance antidepressants (ADMs) for at least two years after remission to manage their ongoing risk of relapse and recurrence.[4] Indeed, there has been a substantial increase in ADM prescribing observed in the UK over the past two decades, largely due to increases in the number of patients on long-term medication.[5] However, many people report difficulties when trying to taper and discontinue ADMs,[6] and healthcare professionals are not always confident in supporting their patients with the process of ADM discontinuation.[7] Research suggests that psychological therapies, such as mindfulness-based cognitive therapy (MBCT) and cognitive-behavioural therapy (CBT), may support people to discontinue ADMs without increasing risk of depressive relapse or recurrence.[8-10] Therefore, it may be useful for healthcare professionals to recommend psychological therapy to their patients wishing to taper and discontinue ADMs. However, more work is needed to understand people's experience of undertaking psychological therapy alongside ADM tapering and discontinuation. This paper explores how people with a history of recurrent depression describe and make decisions about discontinuing ADMs with the support of MBCT.

People describe a number of reasons for wanting to discontinue ADMs, including feeling better and wanting to test whether depression has gone away, ambivalence and uncertainty about the role of ADMs in recovery, side effects outweighing benefits, lack of noticeable effects, questioning whether the self on ADMs is the 'real self', and wanting to assert control over their wellbeing.[11] However, qualitative research has identified a number of practical and psychological barriers to ADM discontinuation, including concerns about withdrawal effects, fear of relapse, perceived lack of alternative coping strategies, and inadequate information about either alternative treatment options or discontinuation.[6] Furthermore, research with healthcare professionals has identified a number of factors that hinder them from supporting patients to discontinue ADMs, including concerns about

For peer review only - http://bmjopen.bmj.com/site/about/guidelines.xhtml

destabilising a currently well patient, and lack of confidence in their professional skills and knowledge regarding discontinuation.[7] Thus, it is clear that more knowledge is needed to understand how best to support people with ADM discontinuation, without risking destabilisation and relapse/recurrence. Clinical guidelines recommend psychological therapies such as CBT and MBCT to reduce ongoing risk of relapse and recurrence.[4] Moreover, several studies have suggested that the skills learned in CBT and MBCT could support ADM discontinuation without increasing the risk of depressive relapse and recurrence.[8-10] Despite this, understanding of the role of psychological therapy in supporting ADM discontinuation and depression relapse prevention is still emerging. In a recent thematic synthesis of barriers and facilitators to discontinuing ADMs,[6] only two studies discussed participants undertaking psychological therapy alongside ADM use and discontinuation,[12,13] with the latter study focusing on the role of treatment in restructuring of participants' sense of self and illness experience, rather than the question of how and why some people, and not others, successfully discontinued their ADMs.

This study considered the experiences of people contemplating, commencing, and completing psychological therapy-supported ADM discontinuation. It was embedded within a randomised controlled trial,[14] which compared two strategies to help people with recurrent depression to stay well over two years: MBCT with tapering support to facilitate discontinuation of maintenance ADMs (hereafter MBCT-TS), and maintenance ADMs. The study examined qualitative accounts from participants who took part in MBCT-TS, whose experiences differed with respect to treatment outcomes (e.g. those who relapsed to depression and those who did not), and ADM discontinuation profiles (those who remained on ADMs and those who tapered, discontinued, or resumed). We investigated how participants described the impact of MBCT-TS on their patterns of ADM usage over a 24-month follow-up period, to try to understand why some people, but not others, discontinued ADMs with the support of psychological therapy. These findings could inform decision-making between clinicians and patients about the journey of management and recovery from recurrent depression.

For peer review only - http://bmjopen.bmj.com/site/about/guidelines.xhtml

METHODS

Study context

This qualitative process evaluation was embedded within the PREVENT trial, a multicentre, single blind, parallel randomised controlled trial, which investigated whether MBCT-TS (n = 212) was superior to maintenance ADMs (n = 212) for the prevention of depressive relapse or recurrence over 24 months (trial design is described in Kuyken et al.).[14-17] The trial found that MBCT-TS was not superior to maintenance ADM in preventing depressive relapse over two years; both treatments had similar outcomes in terms of depressive relapse, as well as residual depressive symptoms and quality of life.[14] The South West Research Ethics Committee approved the trial [09/H0206/43], which was registered with the International Standard Randomised Controlled Trial Register [ISRCTN26666654] and the Medicines and Healthcare products Regulatory Agency [2009-012428-10]. We present a Statement Concerning Reflexivity in the online supplementary materials, which outlines the experience and background of the authors, to acknowledge our theoretical positions and values in relation to the present study.[18]

Participants

Participants in the PREVENT trial were recruited from 95 general practices in urban and rural settings in four UK centres, in addition to self-referral.[14] Inclusion criteria were a diagnosis of recurrent major depressive disorder in full or partial remission according to the Diagnostic and Statistical Manual of Mental Disorders-IV;[19] three or more previous major depressive episodes; age 18 years or older; and on a therapeutic dose of maintenance antidepressant drugs in line with the British National Formulary (BNF) and NICE guidance.[4] Exclusion criteria were a current major depressive episode, comorbid diagnoses of current substance misuse; organic brain damage; current or past psychosis, including bipolar disorder; persistent antisocial behaviour; persistent self-injury needing clinical management or therapy; and formal concurrent psychotherapy. All participants gave informed consent before participating in the trial. The full process of recruitment for the PREVENT trial is described in Kuyken et al.[17]

For peer review only - http://bmjopen.bmj.com/site/about/guidelines.xhtml

The present study examined a sub-group of participants from the PREVENT trial (n = 42) allocated to the MBCT-TS arm of the trial. Of the 212 participants allocated to receive MBCT-TS, 176 received an adequate dose of treatment (attended four or more group sessions of therapy).[14] The researchers purposively sampled a sub-group of these participants (n = 46) to represent a spread of characteristics and experiences with respect to: whether they reported their childhood as having higher or lower levels of abuse, treatment response (relapse/no relapse to a major depressive episode), and ADM discontinuation profile across the 24 month follow-up period (discontinued ADMs, discontinued ADMs but subsequently resumed them, tapered ADMs but never fully discontinued, never tapered or discontinued ADMs).[14] Of the 46 people invited to interview, 42 agreed, which comprised the final sample. Of the four who declined, two had moved away from the area, one was not interested in participating and one participant had changed their contact details and could not be reached. Interviewees did not differ in either baseline characteristics or trial outcomes from broader sample of PREVENT participants randomised to MBCT-TS (see Table 1).

For peer review only

Table 1. Characteristics of the sample.

	Interviewed (N = 42)	All MBCT-TS Participants (N = 212)
Demographic Characteristics		
Female (%)	31 (74)	151 (71)
White (%)	42 (100)	210 (99)
Age (in years)		
M (*SD*)	51.88 (10.51)	50 (12)
Range	25 – 72	22 – 78
Psychiatric Characteristics		
Previous episodes		
< 6 episodes	26 (62)	120 (57)
≥ 6 episodes	16 (38)	92 (43)
Co-morbid mental health diagnoses		
1 or more (%)	15 (36)	75 (35)
Treatment preference at Baseline		
MBCT-TS preference (%)	34 (81)	150 (71)
ADM preference (%)	1 (2)	12 (6)
No preference (%)	7 (17)	50 (24)
Treatment outcome		
Relapse		
n (%) that relapsed during the follow-up phase	23 (55)	94 (44)
Antidepressant usage during the follow-up phase		
Stopped and stayed stopped (%)	13 (31)	67 (32)
Stopped and resumed (%)	9 (21)	57 (27)
Reduced but never stopped (%)	9 (21)	29 (14)
Never stopped or reduced (%)	11 (26)	23 (11)
Residual depression symptoms		
BDI score at baseline, *M* (*SD*)	15.90 (11.35)	13.8 (12.4)
BDI score at 24 month follow-up, *M* (*SD*)	12.39 (12.25)	11.6 (10.9)

Note. BDI = Beck Depression Inventory

For peer review only - http://bmjopen.bmj.com/site/about/guidelines.xhtml

MBCT-TS intervention

MBCT-TS comprised MBCT delivered in line with the published treatment manual,[20] but adapted to include a greater focus on developing a relapse/recurrence signature and response plan that explicitly included participants' reduction/discontinuation of ADM (see Kuyken et al.[17] for more detail). The programme involved eight 2¼-hour group sessions, normally over consecutive weeks, with up to four refresher sessions offered in the year following the end of the eight-week programme. Researchers encouraged participants in the MBCT-TS arm to taper and discontinue their maintenance ADMs at several points from the middle of the MBCT-TS course onwards, and provided information to General Practitioners (GPs) and participants about typical tapering/discontinuation regimes and possible withdrawal effects. If participants experienced a relapse/recurrence during the course of the trial, researchers encouraged them to discuss the most appropriate treatment with their GP and made no further requests that they consider tapering/discontinuing their ADMs. Participants in the MBCT-TS arm who did not taper or discontinue their ADM remained in the trial.

Qualitative data collection

The present study used interviews and written feedback booklets to gather participants' experiences of MBCT-TS and ADM use during the trial. We combined each participant's interview and written feedback booklet data to form a single account of their experiences during the trial and used this as the basis of the analyses in the current report.

Interviews

Interviews were semi-structured and conducted face-to-face in participants' homes by trained researchers, approximately 24 months after MBCT-TS. They lasted between 45 minutes and one hour and explored experiences during the follow-up period, with questions addressing times of wellness, early signs of potential depressive relapse, and relapses. Questions explored the use and perceived value of mindfulness techniques, use of ADMs, and their combination. We tailored interviews to the specific profile of each participant using a 'timeline' prepared in advance and amended by the participant at the

For peer review only - http://bmjopen.bmj.com/site/about/guidelines.xhtml

interview, which summarised each participant's ADM use, relapses, and significant life events, as reported to the research team during the trial. The interviews were deliberately broad in focus to allow for the use of arising data to answer a number of distinct research questions, of which the role of MBCT-TS in ADM tapering was one. Interviews were recorded and transcribed for analysis. The interview schedule is provided in the online supplementary materials.

Written feedback booklets

One month after completing MBCT-TS all trial participants were invited to complete a feedback booklet addressing attitudes towards, and experiences of, taking and reducing antidepressant medication; experiences of taking part in MBCT-TS, and MBCT-TS practices; and the impact of MBCT. In addition to the above, participants received a further feedback booklet 24 months later, which asked the same questions as the first booklet but also included questions focussed on participants' experiences in the follow-up period and basic data on the amount and type of mindfulness practice. The booklets are provided in the online supplementary materials. Mixed methods analysis of feedback booklet data from the complete sample, including participants who did not complete a qualitative interview and those who were allocated to the non-MBCT-TS arm of the trial, will be reported elsewhere.

Public and patient involvement

The PREVENT trial benefited from the expertise of many people with lived experience of mental health difficulties including a number of members of a locally organised voluntary group called the Lived Experience Group (LEG). The LEG assisted the PREVENT trial at every stage of its development including both the interview schedule and written feedback booklets. There were reviewed and then trialled by several members of the LEG who suggested a number of fundamental changes. A member of the LEG provided specific training to the research staff that conducted interviews and also attended all Trial Management Group and Trial Steering Committee meetings.

Data Analyses

For peer review only - http://bmjopen.bmj.com/site/about/guidelines.xhtml

For peer review only

We used thematic analysis as our analytic approach.[18] First, we selected eight participants with a range of ADM discontinuation journeys during the trial period: two who had discontinued ADMs and remained ADM-free; two who had discontinued ADMs and subsequently resumed; two who had never tapered or discontinued ADMs; and two who had tapered but never discontinued ADMs. Four researchers (AT, CC, JR and WK) independently analysed the interview transcripts and accompanying one-month and 24-month feedback booklets for each participant. In this phase, we conducted inductive analysis, with each researcher developing a preliminary coding frame. These frames were then integrated through discussion to remove redundancies and ensure breadth. This collaboratively produced, inductive coding frame was then combined with deductive codes developed from key literature on participant experiences of MBCT,[21] and ADM use,[22,11] to establish a working coding frame.

The lead researcher (AT) then analysed the 42 interviews and accompanying booklets against this coding frame, using NVivo 11 software. AT, CC, and JR met at regular intervals to discuss additional emerging codes and arising themes and, if deemed appropriate, integrated these into the coding frame. Midway through coding, AT sought peer feedback through (1) presentation of emerging findings at an internal research meeting and (2) at a symposium focused on antidepressant tapering at an international conference.[23] Feedback from these presentations helped clarify which themes were particularly important, and in particular helped the researchers reflect on those that related specifically to the *interaction between* the experience of ADM tapering and discontinuation and learning from MBCT-TS. Once the data were fully coded, the researchers reviewed the themes in the light of the core research question. These were discussed with the wider authorship group, whose input was used to reduce redundancy across themes, and highlight their interactions. Finally, we identified indicative cases that illustrated the different ways in which the themes coalesced within individuals to influence their experiences of ADM use over the 24-month period.

For peer review only - http://bmjopen.bmj.com/site/about/guidelines.xhtml

RESULTS

The qualitative analysis of the written and interview data yielded seven over-arching themes, each with a number of constituent sub-themes (see Table 2). Below we provide a narrative account of each theme and its constituent sub-themes, illustrating these with extracts from participants' accounts. Emergent themes overlap at a conceptual level and relate in complex ways within individual cases. Each person's narrative, whilst sharing common themes, also described a unique journey over the follow-up period. Therefore, in addition to describing the distinct themes, we also present five case examples to illustrate some of these different journeys as people participate in an MBCT-TS programme and consider ADM tapering/discontinuation (Box 1). The cases were selected to represent the commonality of themes but diversity of experiences and individual stories of the people in our study.

Table 2: Summary of Themes

Theme	Sub-themes
**Beliefs about the causes of depression**	*Neurochemical disruption:* "serotonin deficiency" as a dominant belief about the cause of depression.<br>*Learning a new model*: the impact of learning psychological model and techniques on decisions about ADM tapering/discontinuation.<br>*Bridging models:* people's attempts at integrating their diverse perspectives on depression.
**Control**	*Managing Expectations:* feeling optimistic or sceptical about gaining control over ADM use/discontinuation.<br>*Agency and responsibility:* more agency and responsibility to manage depression symptoms by learning psychological skills that could take the place of ADMs.
**Acceptance**	*Resolving shame and denial*: psychological therapy supporting resolution of shame and denial surrounding depression and ADMs.<br>*Self-care*: more favourable attitudes towards self-care, using

For peer review only - http://bmjopen.bmj.com/site/about/guidelines.xhtml

	psychological techniques and/or ADMs.<br>*Perspectives on relapse*: diverse and evolving attitudes towards depressive relapse/recurrence.
**Quality of Life**	*Experiencing emotions more fully*: the impact of different treatments on the experience of emotions.<br>*From coping to enjoying life*: Psychological therapy helping people to move beyond coping towards enjoying life.
**Tapering process**	*Pace of reduction*: decisions about method of tapering ADMs and impact on subsequent outcomes.<br>*Managing withdrawal effects*: psychological therapy helping to cope with the period of withdrawal symptoms.
**Interactions with GP**	*Presence and support*: availability and responsiveness of GP and impact on ADM tapering/discontinuation.<br>*Following advice*: different attitudes towards seeking and following GP advice on tapering/discontinuation.
**Timing**	*Feeling vulnerable*: current depression symptoms and difficult life circumstances contributing to reservations about ADM tapering/discontinuation.<br>*Sequencing of treatments*: reflections on potential sequencing of different treatments to support recovery from recurrent depression.

For peer review only - http://bmjopen.bmj.com/site/about/guidelines.xhtml

Beliefs about the causes and treatment of depression

This over-arching theme describes participants' beliefs about the causes of depression and articulates how these beliefs influenced their treatment decisions. Prior to the programme, many participants described their understanding of depression as caused by a neurochemical disruption of neurotransmitters in the brain. During MBCT-TS they learned a psychological, cognitive-behavioural model of depression, and how this model could be used to develop strategies to prevent depressive relapse and recurrence. Whether people learned and used this model was variable, and seemed to be influenced by their initial experiences of treatment, which had implications for their subsequent treatment decisions. This theme comprises three sub-themes.

Neurochemical disruption

Prior to the programme, many participants thought they had experienced recurrent depression due to a neurochemical disruption in their brain, often citing specifically a deficiency or imbalance of the neurotransmitter serotonin. Participants viewed medication as a way to correct this issue and made parallels to biomedical disorders, viewing ADMs as a *"physiological need"* in the same way that *"diabetics require insulin"* because *"there is some chemical missing." (2102; Written feedback, Never tapered or discontinued)*. For instance, Annie explained that she went on ADMs because her doctor told her that she had lower levels of serotonin than other people (see Box 1). This belief appeared to influence expectations about psychological therapy, as some participants stated that they did not understand how "*mindfulness would be able to counteract depression […] if it's generated by a chemical imbalance." (1031, Interview, Never tapered or discontinued)*. Other people said that they had not given much thought to why they were depressed or how ADMs worked: *"Happy pills […] I've never really given it a great deal of thought exactly what they do to be honest. […] I just know I don't feel so bad with them. (2123, Interview, Tapered but never discontinued).*

Learning a new model

For peer review only - http://bmjopen.bmj.com/site/about/guidelines.xhtml

Participants described how their views on the causes of depression evolved during and following the MBCT-TS programme. Despite some of the initial reservations described above, participants described an open mind as key to engaging with the new psychological model, in which their thoughts, behaviours, and emotions played a role in depressive relapses and recurrences: *"in the first sessions […] I switched from being highly sceptical to very interested very quickly" (1203, Interview, Discontinued).* Some participants articulated a move away from *"treating depression as a disease, like if you had a toothache, so you took pills"*, and were surprised because they "*hadn't thought that there was an alternative" (1069, Interview, Discontinued)*. They began to feel confident to discontinue ADMs with the support of psychological therapy. In addition, people described how the programme gave them more awareness of how external factors, such as relationships or financial situations, could trigger or exacerbate depressive relapses and recurrences. On the other hand, some participants found it more difficult to engage in the programme and found themselves *"rebelling against it"* because they did not have *"intellectual confidence in the process." (3105; Written feedback, Never tapered or discontinued).* People described how their initial treatment experiences influenced their attitudes: those who felt that the techniques were helping them to manage depressive relapse/recurrence often endorsed the psychological model. On the other hand, others who experienced deterioration in mood or relapse sometimes reported that they had re-considered bio-medical explanations, and decided to resume or remain on ADMs: *"I really thought depression was a psychosomatic problem, but I am not so sure now. I did give it my best shot, using the mindful techniques, but I still fell into the pit of despair […] I feel that my depression is caused by a chemical imbalance in my body which, at present, is only helped by taking medication." (2200; Written feedback, Tapered but never discontinued).*

Bridging models

Although some participants took a dualistic stance of depression as either biomedical or psychological, favouring one model over the other, many did not see the two models as distinct and found ways to integrate these models. For instance, they conceptualised that *"antidepressants hold onto the chemical in your body 'cause you're not making enough of it yourself"*, while MBCT-TS allows you to *"focus your mind onto how to make your own." (1139, Interview, Never tapered or*

For peer review only - http://bmjopen.bmj.com/site/about/guidelines.xhtml

discontinued). It seemed that participants who viewed these models as compatible were more open to using ADMs and using psychological techniques as an additional way to support their recovery, rather than viewing them as competing treatments. Furthermore, when participants observed the diversity of other people's experiences on the programme, some formed the opinion that there are *"all sorts of depressions"* underpinned by different causes *"just as there are colds and flu's and viruses." (3105, Interview, Never tapered or discontinued).* As such, some reasoned that different people would require different treatment decisions to support recovery: *"My depression is not necessarily the same as other people's […] The right combination of changing lifestyle, specific therapies, medication whatever else it takes – that seems to be different for different people." (3109, Interview, Never tapered or discontinued).*

Control

This over-arching theme describes people's perceived sense of control over their ability to discontinue ADMs. As captured in the written data and when reflecting on experiences with ADMs and therapies prior to the programme, people expressed various fears about the process of ADM discontinuation, but many hoped that a psychological programme could support them and give them more control over their treatment choices. During MBCT-TS, people spoke about being better able to manage their vulnerability to depressive relapse, by using the skills and techniques they learned on the programme. While enhanced feelings of personal agency were largely viewed as a positive and increased many people's confidence to taper and discontinue ADMs, this was not always the case, and some felt the pressure of having more responsibility to manage their condition. This theme comprises two sub-themes.

Managing expectations

Before the trial, many people expressed a preference to come off their ADMs, but said they remained on ADMs because they felt out of control and afraid that depression would return if they discontinued ADMs: *"[I] definitely didn't want to regress as the last bout of depression was my worst. [I was] at a stage where I had almost accepted taking long-term medication although not entirely happy.*

For peer review only - http://bmjopen.bmj.com/site/about/guidelines.xhtml

I knew they helped and didn't have anything else to take the place of the tablets." (1087; Written feedback, Tapered but never discontinued). Many participants said they agreed to participate in the trial because they believed psychological therapy would teach them skills to manage discontinuation, and liked *"the idea of coming off the drugs in a controlled, relatively safe environment and of being offered the "tools" to cope without them." (4057; Written feedback, Discontinued)*. Others however had much lower expectations based on their notions of what the programme would entail and were *"very sceptical"*, and thought *"it's not going to work because I can't change my life drastically like that." (2016, Interview, Discontinued and resumed, then discontinued*).

Agency and responsibility

After taking part in the programme, many participants described a change in their sense of control about depression, describing a shift in personal agency from a *"helpless victim of circumstance"*, to having more *"control of my feelings and my life" (1123; Written feedback, Tapered but never discontinued)*. For instance, George said that before the course, he would fall into depressive episodes very suddenly and without warning, whereas the mindfulness skills gave him more awareness and control to act and prevent relapses before they occurred (see Box 1). In general, participants attributed to the skills and techniques they had learned on the programme, which helped them to manage their vulnerability to depressive relapse and recurrence. For instance, they reported an increased ability to recognise the early warning signs of depressive relapse and take steps to respond, by applying mindfulness or cognitive-behavioural techniques from a 'toolbox', including things like meditation, activity scheduling, or enlisting social support: *"Before the trial, I didn't have the tools to recognise what was happening. [...] I didn't even know I was getting depressed. [Now] if things are difficult I can do something about it." (1203, Interview, Discontinued).* This reduced many people's fears about coming off ADMs, because they felt they had the capacity to prevent or contain depressive relapses. Participants also articulated that learning how they could have more agency over their thoughts, feelings, and behaviours led to an increased sense of responsibility to manage their wellbeing. Most participants viewed this as positive, especially if they were able to use the techniques to manage relapse/recurrence. Some people said they preferred this approach to resuming ADMs, because it made

For peer review only - http://bmjopen.bmj.com/site/about/guidelines.xhtml

recovery feel more like a personal achievement: *"Once I've fallen and I realise that I am depressed, I take myself off and say do 3 or 4 meditations a day. […] Which to me is better than taking a pill, because I know I've worked to get myself well." (2016, Interview; Written feedback, Discontinued and resumed, then discontinued).*

However, not all participants viewed it positively to have more agency and responsibility over their wellbeing. In particular, some participants described how this made them feel like it was their fault if they relapsed or felt they had to resume ADMs: *"I feel sad and disappointed that stopping [ADMs] made me feel low again. […] It makes me feel I'm not right in the head compared to others. I also feel annoyed with myself for not utilising MBCT skills learnt better." (2123; Written feedback, Discontinued and resumed).* Furthermore, a substantial number of participants expressed the challenge of finding the time, motivation, or self-discipline to keep up a regular mindfulness practice outside of the group sessions. Therefore, the sense of control did not always feel stable, as it was contingent on finding time to practice and *"do it religiously, otherwise I would be fearful of it not being enough." (2102; Written feedback, Never tapered or discontinued).* Some were disappointed when they realised that psychological therapy was not an *"all-encompassing cure" (1222, Interview, Discontinued and resumed)* and would involve an active and ongoing process of engagement with the techniques learned.

Acceptance

This over-arching theme describes people's feelings of acceptance towards their history of depression and their ongoing need to manage their risk of relapse and recurrence. Before the trial, many people expressed a sense of shame around taking ADMs, feeling they labelled them as an ill person. After the trial, people described an increased sense of acceptance regarding depression, and more motivation to engage in self-care to support their ongoing recovery. This self-management included either ADMs and/or the psychological techniques for different people. This theme comprises three sub-themes.

Resolving shame and denial

For peer review only - http://bmjopen.bmj.com/site/about/guidelines.xhtml

Before the trial, many described feelings of low self-esteem, feeling *"inadequate"* or unable to cope with life compared to other people, and treating depression as a *"guilty secret" (1123; Interview, Tapered but never discontinued).* Taking ADMs had helped some to resolve this, by reducing the symptoms and allowing them to return to being a *"normal contributing person in society" (2200; Interview, Tapered but never discontinued).* However, others expressed that taking ADMs still labelled them with a disease, experiencing an underlying feeling that they were *"not a well person" (2102; Interviews, Never tapered or discontinued)* if they needed to take medication. For these reasons, some people described how before the trial, they were in denial about having depression, and *"couldn't even or wouldn't even admit to that." (1031, Interview, Never tapered or discontinued)*. After the programme, some participants described how for the first time, they felt able to name their condition as depression. Participants discussed non-specific effects around normalisation within a group setting, i.e. how meeting other people in the programme had made them realise that depression was not a negative aspect of their own self-identity, but an aspect of common human experience: *"You realise it is part of the human condition rather than you."(1128; Interview, Never tapered or discontinued)*, and it *"confirmed that I am a human, worthwhile person" (2176; Written feedback, Discontinued and resumed)*. This led to increased feelings of acceptance towards depression, because participants experienced a shift away from viewing themselves as abnormal, to seeing depression as a more acceptable response to life's difficulties: *"Giving yourself credit [...] 'cause at the end of the day [...] our human brain is quite a complex thing, isn't it? [...] There's nothing wrong in feeling like it." (2140; Interview, Discontinued and resumed).*

Self-care

Participants described how developing more acceptance towards their condition affected their attitudes towards self-care. They said that accepting their vulnerability to depression allowed them to *"look at solutions"* and that they finally had "*consent to actually do something about it" (1031, Interview, Never tapered or discontinued)*. People described how they increasingly accepted that they needed to take care of themselves, and explained how the programme had taught them legitimate ways to do this, such as using skills like meditation: *"Previously was a mindset [...] that I wasn't allowed to*

For peer review only - http://bmjopen.bmj.com/site/about/guidelines.xhtml

help myself feel better. […] Whereas this felt a way that I could do it without mollycoddling myself." (1031, Interview, Never tapered or discontinued). Participants also described how the programme had reframed self-care not as something *"fluffy"*, but as *"practical"* and a necessary part of their ongoing recovery: *"It doesn't make you any less male of course. [Chuckles] Or any less powerful." (1203, Interview, Discontinued).* In some cases, this new attitude towards self-care caused a shift such that people felt more acceptance towards taking ADMs: *"I don't feel any more when I take my pill every morning that there's something wrong with me"* as they recognised it was important to do *"everything in my power to help myself." (1177, Interview, Tapered but never discontinued).* Some participants also described how originally they had taken ADMs unwillingly, whereas now they took ADMs as an act of effective self-management: *"I used to hate taking them [ADMs] I accept [now] it's all about looking after yourself isn't it?" (3103; Interview, Discontinued and resumed).*

Perspectives on relapse

Participants also described a change in their feelings of acceptance towards depressive relapse and recurrence. Although many people did not experience a depressive episode during the trial period, those who did described varying perspectives on this. For instance, when Greta experienced a deterioration in mood, she interpreted this as a sign that the programme had been a *"failure"* and she resumed taking ADMs (see Box 1). However, this was not always the case, and many people described how participating in MBCT-TS changed their attitude towards relapse/recurrence. In particular, some people felt more able to accept periods of depression and approach them in a different way, "*thinking it was a phase that one was going through and sort of accepting, okay this is how you're feeling today" (1159; Interview, Discontinued and resumed).* Some people reported that they no longer wanted to *"blank out their negative emotions"*, and so did not resume ADMs, even if they relapsed: *"it's definitely helped me to realise that they [negative emotions] are a part of me as well." (4057, Interview, Discontinued).* Many people felt more resourced by the programme, and said that they could better manage the periods of low mood or depression. This corresponds with George's experience. George did not resume ADMs when he relapsed, and instead decided to practice MBCT-TS skills, which helped him to reach remission again (see Box 1).

For peer review only - http://bmjopen.bmj.com/site/about/guidelines.xhtml

Quality of Life

People reflected on the ways in which different treatments influenced their overall quality of life. They discussed how decision-making around ADM tapering and discontinuation reflected a weighing up of different priorities: on the one hand, to stave off depression, and on the other, to restore positive emotionality into their lives. People also discussed how the programme supported them to move from a place of coping, to a position where they could enjoy and appreciate their lives, regardless of whether they tapered and/or discontinued ADM or remained on them. This theme comprises two sub-themes.

Experiencing emotions more fully

Upon reflecting on their experiences with ADMs, some participants said that while ADMs lessened their low mood, at the same time they *"dampen all other emotions"*, for instance, they could not feel *"blissfully happy, couldn't get angry, and in hindsight feel I was sedated." (4057, Written feedback, Discontinued).* In the context of depression, some people viewed this numbing effect as helpful, and reflected that while ADMs "*take away the euphoria that you would get when you've done something really, really, really good"*, this was *"a small price to pay really for not having the really dark times." (2200; Interviews, Tapered but never discontinued).* However, many people thought that this had negatively affected their quality of life, especially in the cases where they found it hard to experience positive emotions. This appeared to influence people's decision to taper or discontinue ADMs, because they said that restoring their emotional range was an important part of their long-term vision of recovery: both George and Claire described this as a key motivator to discontinue their ADMs (see Box 1). Indeed, people described how their emotional capacity increased after coming off ADMs: *"I am more alive: my emotions aren't "levelled out" anymore. I can be happy, sad, angry or calm instead of just bland." (4057, Written feedback, Discontinued)*, albeit some people found it a bit of a *"shock"* at first, when faced with *"very extreme emotions and feelings"* again *(1212; Feedback booklets, Discontinued)*. Therefore, people found it helpful that the programme taught them techniques to help manage this transition: *"I definitely used mindfulness during coming off the tablets to [...] be*

For peer review only - http://bmjopen.bmj.com/site/about/guidelines.xhtml

aware what's going on inside and […] calm myself down, to have those little islands of tranquillity." (4057, Written feedback, Discontinued). On the other hand, some participants said that despite not tapering or discontinuing ADMs, the programme had helped them to cultivate more positive emotions, and appeared to increase their quality of life on ADMs: *"I suppose the mindfulness in that respect has helped because […] by slowing yourself down you can […] capture some of that […] joy of life that possibly I would have lost." (2200; Interviews, Tapered but never discontinued).*

From coping to enjoying life

Many people reflected that, given their history of depression, they had been grateful for the periods of time where they were simply coping or functioning. However, they said that the programme had helped them to move beyond that mind-set, and to develop more wellbeing and appreciate life: *"What has changed? I think my outlook on life, I love life, I really do […] People said to me […] before you used to skulk into the room, now you light up the room. […] I do enjoy life now, where I didn't before." (2016, Interview, Discontinued, resumed, then discontinued).* They valued that the programme had an active focus on positive functioning, and encouraged them to take part in activities that brought happiness and joy into their life. Participants described this as an active process, facilitated by a sense of having more control and autonomy over making positive decisions in their life: *"I rearranged my life so that the things I do now are things that I enjoy and want to do." (1203, Interviews, Discontinued).* These benefits were not limited to those people who discontinued ADMs, but were an additive benefit for many participants who remained on or resumed ADMs: *"I am now making bigger future plans to make my life better and introducing new ventures." (1031, Written feedback, Never tapered or discontinued).*

Tapering process

Many participants described the process of tapering and discontinuing ADMs, and attempted to justify the different outcomes that they experienced. This over-arching theme describes participants' descriptions of the tapering process, and articulates what they viewed as helpful or hindering to the process of discontinuation. This theme comprises two sub-themes.

For peer review only - http://bmjopen.bmj.com/site/about/guidelines.xhtml

Pace of reduction

Participants discussed the pace at which they tapered ADMs, and how they perceived this to have influenced their outcomes. Some people who were worried about coming off ADMs shared that they exercised caution, testing out the psychological techniques for a set period and tapering slowly. They said this was helpful as it gave them time to learn to use the psychological techniques before giving up the support of their ADMs, *"by doing it slowly, you are learning those skills and you are finding out how you can use it. [Then] you can start dropping it [ADMs] at your own pace." (1075; Interviews, Discontinued)*. In comparison, those who were keen to come off ADMs and were less fearful of the consequences described tapering more quickly. Although the programme had included explicit guidance to taper gradually, participants' reports suggested that many people had gone against this advice, and were looking for a *"quick fix"* to *"get off the pills as quick as possible" (2131, Interview, Tapered but never discontinued)*. However, upon reflection many people thought, *"perhaps that wasn't the answer perhaps the thing ought to be graded on over a longer period." (2131, Interviews, Tapered but never discontinued)*. Some of these participants reflected that in retrospect they should have been more cautious, and tapering too quickly had led to poorer outcomes: *"I reduced my tablets too quick and paid the price by having to get straight back to the full dose" (2016, Interview; Written feedback, Discontinued and resumed, then discontinued)*. However, some people, like Claire, who did not successfully discontinue on their first attempt reported how they had then tried again, tapering more gradually and with more success (see Box 1).

Managing withdrawal effects

People said that the programme had helped them to cope with withdrawal effects during and after tapering/discontinuing ADMs. They described how the group and the meditation techniques provided ongoing support to manage this period: *"I used meditation techniques [...] tried to treat myself with pleasurable experiences and told myself that this would pass over. [...] I had a network of fellow participants and a trustworthy instructor. All of this put me in a position of confidence that it would work this time." (4057, Written feedback, Discontinued)*. In addition, people said that they were better

For peer review only - http://bmjopen.bmj.com/site/about/guidelines.xhtml

able to differentiate the side effects of ADM withdrawal from a depressive relapse. For instance, Mandy said that in the past, withdrawal effects had been the biggest hindrance to tapering ADMs, because she had always mistaken them for a depressive relapse and resumed her medication. On the programme, she learned how to differentiate between these effects and *"real relapses"*, and said that tapering was relatively *"easy"* this time around (see Box 1). Indeed, some people who had attempted discontinuation before the trial reflected how their GPs may have *"misdiagnosed"* their withdrawal symptoms *"as recurring depression"*, whereas this time they *"knew what was coming" (4057, Written feedback, Discontinued).*

Interactions with GP

Participants' described their interactions with their GPs when contemplating and/or commencing the process of ADM tapering and discontinuation. This over-arching theme captures what aspects of this relationship participants viewed as helpful and the extent to which these interactions influenced ongoing treatment decisions. This theme comprises two sub-themes.

Presence and support

Participants discussed the relationship that they had with their GP and its influence on the tapering process. In general, participants found it supportive if their GPs were easy to access throughout the process of discontinuation: *"Knowing that I could ring the doctor and say, "I need to make an appointment, I need to come and see you." There was always that net underneath me to catch me if I was falling and I couldn't stop it." (2090, Interview, Discontinued)*, whereas some participants said they found it *"very difficult"* to access their GPs, and so felt *"unsupported" (1123, Written feedback, Tapered but never discontinued).* Participants reported a more positive attitude to the programme if their GP had endorsed it, and some said they had only been convinced to take part in the trial because their GPs said they had done a mindfulness course. When GPs encouraged their patients to use the mindfulness practices, this appeared to be associated with better engagement and subsequent success in ADM tapering and discontinuation: *"I did reach a stage where I went to see my G.P. as the depression*

For peer review only - http://bmjopen.bmj.com/site/about/guidelines.xhtml

was returning. […] We decided that I should try the exercises before trying pills. I did not need to go back on them yet […] My GP is a great help." (2090, Written feedback, Discontinued).

Following advice

Participants differed in the extent to which they sought and followed the advice of their GPs. For instance, some participants described that they remained on their ADMs at their GP's suggestion: *"My GP would not allow me to come off my antidepressant or reduce it because I had been on them so long term. [I am] relieved but also a bit disappointed." (1108; Written feedback, Never tapered or discontinued).* This adherence to medical advice seemed to be greater for participants who had more concerns about discontinuation. For instance, Claire, who relapsed the first time that she had attempted tapering and discontinuation, was much more receptive to her GP's advice the second time around, because she was afraid of relapsing again (see Box 1). On the other hand, where people were confident that they had learned the skills to self-manage their depression without ADMs, they more often reported that they could manage the process independently, and placed less value on their GP's advice: *"I went along to the doctors because I was polite to ask him if I could stop taking it. And he said, "Well yeah maybe in a few months time you can taper it- ease it off a bit." But really I had decided (laughs) I was going to stop. So I was just there out of politeness." (1203; Interview, Discontinued).*

Timing

This over-arching theme focuses on participants' reflections on their current sense of vulnerability to depressive relapse and recurrence, which appeared to relate to their external circumstances and current level of depression symptoms. It also describes how this sense of vulnerability influenced their subsequent decisions around ADM tapering and discontinuation. Participants' reflected on what they thought would be the most appropriate timing and sequencing of ADMs and psychological therapy in relation to each other, to support the long-term treatment of depression. This theme comprises two sub-themes.

Feeling vulnerable

For peer review only - http://bmjopen.bmj.com/site/about/guidelines.xhtml

Participants discussed the timing of ADM tapering, and thought it was important to wait for the right time to begin tapering. Those who were experiencing depression symptoms, or whose life circumstances were difficult, often made the choice to remain on antidepressants, so not to *"rock the boat" (1087, Feedback booklet, Tapered but never discontinued).* For instance, Annie began to taper off her ADMs, but then experienced some very upsetting life events, so decided to resume ADMs because she felt more vulnerable to depressive relapse (see Box 1). In these cases, many people viewed the mindfulness practices as a helpful addition to ADMs, and some tapered their dosage even if they did not fully discontinue. On the other hand, other participants who felt depressed said that this interfered with their ability to practice the techniques taught or taper ADMs at all: *"When I was feeling really, really down I couldn't even access those things [the MBCT-TS practices]. It either felt indulgent or I couldn't physically do it."(1031, Interview, Never tapered or discontinued).* Nonetheless, some people said they valued attending the programme, even though they did not continue to use the techniques: *"I think that course it's completely changed my life. [...] Even though I don't sort of practice mindfulness daily just remembering the course [...] it seems to have helped to keep me more level." (2123, Interview, Tapered but never discontinued),* and some expressed hope that they would discontinue ADMs in the future, as a result of the programme: *"One day I may stop my medication because it feels the right time to do so, having a more regular mindfulness routine will be in place by then for support. I'm really hopeful and positive about my future!" (1087, Feedback booklet, Tapered but never discontinued).*

Sequencing of treatments

Reflecting on the right time to engage with different treatments, many participants felt that ADMs were helpful when they first became depressed: *"they got me out of my initial depression so that I could cope more with just everyday life" (4007, Interview, Never tapered or discontinued)*. However, many did not envisage being on ADMs indefinitely, and they described an increasing need for insight and self-management of depression as time went on. They thought that the MBCT-TS techniques required more effort, but supported a longer-term vision of recovery, to *"recognise what makes you depressed and to give you a way to cope with your depression throughout your life for the long-term,*

For peer review only - http://bmjopen.bmj.com/site/about/guidelines.xhtml

and a way that you can come off [ADMs]." (4007, Interview, Never reduced or discontinued). As illustrated by this quotation, some participants viewed the MBCT-TS skills as part of a longer-term solution to ADM discontinuation, which extended beyond the two-year follow-up period. Some participants reflected on how they thought the two treatments could be used in combination to support people at different parts of their journey, from depression through to recovery: "*I think you need that initial boost of antidepressant to perhaps get you back into a more rational level, and then once you've reached that, then bring in the MBCT, until you get back then you know, be weaned off. I can see that working very well really." (1108, Interview, Never reduced or discontinued).*

DISCUSSION

Statement of principal findings

The current study explored the experiences of people with recurrent depression who followed a programme (MBCT-TS), designed to teach psychological skills to prevent depressive relapse whilst providing advice to encourage tapering and discontinuation of maintenance ADMs.[14] Qualitative interviews two years after completing the programme illustrated people's varying beliefs about depression and its treatment, and a diversity of concerns, expectations and hopes about ADM discontinuation. Many participants reported beneficial effects of learning MBCT-TS as an approach to support relapse prevention, which facilitated tapering or full discontinuation of ADMs. However, there were also aspects of people's experiences that contributed to their decisions about whether to remain on ADMs, taper, discontinue, or in some cases discontinue and resume at a later date. The qualitative analysis identified seven over-arching themes in participants' accounts of their decisions relating to ADM (dis)continuation: beliefs about the causes of depression, control, acceptance, quality of life, tapering process, interactions with GP, and timing (Table 2). These themes help to explain why some people discontinued, while other people remained on or resumed ADMs across the two-year trial period. We also identified indicative cases that illustrated the different ways in which these themes coalesced within individuals to influence their journeys of ADM use across the programme (Box 1). Together, these findings have the potential to facilitate discussions between clinicians and

For peer review only - http://bmjopen.bmj.com/site/about/guidelines.xhtml

patients about the journey of management and recovery from recurrent depression. The findings also provide an important starting point for more research into which treatments for recurrent depression, or combination of treatments, work best for whom and when.

Strengths and weaknesses of the study

This study had a number of methodological strengths including the relatively large sample for a study of this nature, and the sampling approach that captured a range of perspectives. In addition, because participants were followed over two years, the study enabled us to develop an understanding not just of experiences of MBCT-TS itself, but also its impact on participants' use of ADMs in the subsequent two years. To overcome difficulties with recollection we supported interviewees with prompts about the course of their depression and ADM use over the two-year period based on information they had provided to the study immediately after MBCT-TS and during the trial follow-ups. Finally, MBCT-TS is a programme based on a cognitive-behavioural model of depression. Therefore, it is likely that people's experiences of tapering and discontinuing ADMs in the context of MBCT-TS would have many similarities with people in CBT, another widely used approach to preventing depressive relapse and recurrence.[24] Nonetheless, the commonalities and particularities of different psychological therapies remain a topic for further research.

Alongside these strengths, it is important to consider the context within which the study took place and its implications for interpretation of the findings. First, the trial was pragmatic in that it recruited participants from primary care who had a history of recurrent depression, were on a maintenance dose of ADMs, and were interested in considering a psychological approach to ADMs for relapse/recurrence prevention.[17] However, it did not include people either unwilling to consider a psychological therapy or unwilling to consider tapering/discontinuing their medication. Second, the parent trial included monitoring participants' use of ADM, and if people following MBCT-TS were not tapering/discontinuing they were invited to discuss this with their GPs. Some participants reported feeling pressured to discontinue and it is reasonable to assume that some participants may have made different decisions in a more naturalistic setting. Third, we sought accounts only from people who

For peer review only - http://bmjopen.bmj.com/site/about/guidelines.xhtml

attended at least four sessions of MBCT-TS. Whilst this enabled us to capture the perspectives of people who had developed some understanding of what MBCT-TS could offer, it also means that the voices of those who did not engage with the treatment at all were not included.

Strengths and weaknesses in relation to other studies, discussing important differences in results

The majority of previous qualitative work has focussed broadly on people's experiences of ADM use and discontinuation.[6,11] The present study adds to the body of literature suggesting that people with a history of recurrent depression may wish to discontinue their maintenance ADMs but experience a number of practical and psychological barriers to achieving this, such as concerns about withdrawal effects, fear of relapse, perceived lack of alternative coping strategies, and inadequate information about discontinuation. Several randomised controlled trials have demonstrated that psychological therapies such as CBT and MBCT could support ADM discontinuation,[8-10] but to our knowledge, no qualitative studies have examined people's experiences of this process. Our findings support the idea that psychological therapy can help to address many of the barriers and provide facilitators to tapering/discontinuation of ADMs identified in the previous literature.[6] For instance, participants reported that MBCT-TS helped them by teaching new skills to manage depressive symptoms, gaining new perspectives drawn from both the psychological model and peer-to-peer learning, and developing an increased sense of agency concerning ADM discontinuation. We also found that engaging in psychological therapy supported learning attitudes towards self-care that were participatory and empowering, which facilitated ADM discontinuation through increasing people's motivation to engage in psychological tools as an alternative to taking ADMs. People also emphasised the importance of providing clear, simple information about the process of discontinuation and having a GP to provide support during discontinuation attempts that is collaborative, individualised and empowering, with careful monitoring over time.

Kendrick has argued that many people remain on ADMs without clinical need and could benefit from support and guidance on how to discontinue, especially regarding how to deal with initial

For peer review only - http://bmjopen.bmj.com/site/about/guidelines.xhtml

withdrawal symptoms.[25] Our findings support this, for example suggesting that psychological therapy can enable people to better differentiate physical and mental symptoms related to withdrawal from those related to depressive relapse. However, they also suggest that for people with a history of recurrent depression, simple tapering support alone may not be enough. Participants spoke of the importance of feeling that they had acquired alternative skills to manage their depressive symptoms that could take the place of ADMs, rather than simply skills to manage the tapering process itself. For these participants, there was an ongoing sense of clinical need. Further, where life circumstances were challenging some people felt that the time was not right for them to discontinue ADMs and they made an informed decision to continue with their medication. Even so, the majority of participants who remained on ADMs reported that the MBCT-TS treatment had increased their quality of life on ADMs, and improved their confidence in future discontinuation when circumstances were more favourable. Our analysis also outlines participants' views on the appropriate timing of different treatments, which could give clinicians a sense of when it might be an appropriate time to initiate conversations about ADM tapering and discontinuation, and when it might be helpful to refer a patient for psychological therapy.

People's expectations at the outset of the psychological treatment appeared to influence their engagement and subsequent outcomes. Although it is widely assumed that positive expectations predict greater benefit in psychological therapy, in our sample both unrealistically positive expectations (e.g. expecting MBCT-TS to be an *"all-encompassing cure-all"*) and very negative expectations (having *"no intellectual confidence in the process"*) appeared to act as a barrier to engagement. These findings are consistent with those of Malpass et al.[26] and suggest that openly discussing expectations as part of the psychological therapy referral or orientation process is likely to be key in preventing disappointment or disengagement from what is an effortful process of change. Likewise, in line with Maund et al.'s findings,[6] people's causal models of depression also appeared to influence their expectations and engagement with psychological therapy. Moreover, they were subject to change during and beyond the therapy process, as their experiences either confirmed or disconfirmed their expectations and working model of depression and its treatment. People who suffer from depression frequently endorse biomedical explanations,[27] and this was evident in our sample. A number of people reported

For peer review only - http://bmjopen.bmj.com/site/about/guidelines.xhtml

that they derived these models from discussions with their GPs as a rationale for taking ADMs. Previous research suggests that conceiving depression as a biomedical illness can absolve people of personal responsibility and thus challenge stereotypes of depression resulting from personal weakness.[11] However, our findings suggest that strongly held biomedical beliefs appeared to increase feelings of dependency on ADMs, and contribute to negative expectations and lack of engagement with psychological therapy. On the other hand, while learning a psychological model of depression empowered people towards more self-management of depression and feelings of mastery over their emotional wellbeing, in some cases, when people developed a psychological understanding and then went on to relapse, they blamed themselves. In some cases, practical life circumstances also made it very difficult for people to engage in an approach that required time and effort. Together, this suggests that polarised beliefs about the causes of depression can either compromise self-efficacy or promote self-blame. Many participants found it helpful to bridge biomedical and psychological theories, with parallels to a 'biopsychosocial' framework,[28] rather than viewing separate theories as competing, which seemed to foster more flexibility, self-compassion and open-mindedness towards trying different treatment options at different times in their journey of managing recurrent depression. This highlights the importance of recognising that a myriad of factors, including genetic vulnerability and challenging social circumstances can influence depression.

The present study also built on existing work highlighting the idiosyncratic ways that ADMs pharmacologically affect the mind and body through sedation, numbing, and activation,[29] and went further by highlighting how these effects influence people's decisions in the process of discontinuation. For example, in the instance of numbing, some people viewed this as helpful as it reduced their feelings of depression, whereas other people said that ADMs numbed all of their emotions, including positive feelings, and this contributed towards a desire to discontinue them. These findings add to ongoing discussion about the psychoactive effects of ADMs, including their potential benefits and costs, how these effects impact people's experience of recovery from depression, and how participating in psychological therapy can interact with these experiences.

For peer review only - http://bmjopen.bmj.com/site/about/guidelines.xhtml

Finally, descriptions of the role of the GP in supporting ADM discontinuation varied markedly, and this appeared to result both from differences between patients in their preferred level of guidance and support, and the availability of their GPs to provide this. For example, some people adhered to their GP's advice although this was in conflict with their own desired approach, some described informing their GP of their intentions as an act of courtesy, and some did not involve their GP at all. In some of these latter cases, participants felt that they would have benefited from more support, but their GP, for a range of reasons, was unable to provide this. People also described needing more understanding and support over time as they took more responsibility for managing their depression. This is in line with findings from Malpass et al.,[11] who suggested that people vary in the extent to which they want to be involved in treatment decision-making, and their preferences for involvement are dynamic, not static. Archer has described different 'modes of reflexivity' noting the varying degrees to which people act autonomously or rely on endorsement from others.[30] It is likely that when GPs are able to recognise their patients' preferred mode of engagement and adapt their approach accordingly, discontinuation attempts will be more successful.

Meaning of the study: possible explanations and implications for clinicians and policymakers

People with a history of recurrent depression may wish to taper and discontinue maintenance ADMs, but lack the knowledge and confidence to do so. Furthermore, people benefit from having social support during discontinuation attempts, but do not always get the level of support they require from their GPs or existing social networks. This highlights the potential clinical value in providing group-based psychological interventions that support ADM discontinuation. Importantly, both the results of the thematic analysis and the narratives of individual participants suggest that the benefits of psychological therapy to assist ADM tapering/discontinuation are not fully captured by rates of complete discontinuation or relapse rates of depression alone. Rather, recovery meant different things to different people, and overall, the outcome most important to patients appeared to be their day-to-day functioning and quality of life. Within the broader context of people's lives, psychological therapy may increase quality of life, *whether or not* the person successfully discontinues their ADMs or experiences further depressive symptoms. Therefore, many people on ADMs could benefit from concurrent

For peer review only - http://bmjopen.bmj.com/site/about/guidelines.xhtml

psychological therapy even if they do not wish to taper/discontinue, to promote positive emotional states, better management of residual symptoms, to resolve feelings of shame surrounding depression and ADM use, increase personal agency, and to provide people with a broader perspective on their depression.

Whilst some people described the substitution of the support of ADMs with psychological techniques as a relatively straightforward and positive process, for others there was an understanding of the ongoing benefits of ADMs, alone or in combination with psychological therapy. The study also highlighted the importance of optimal timing and sequencing of pharmacological and psychological treatments, and the relative benefits of each treatment at different periods. Therefore, it is crucial that GPs are receptive to their patients' fluctuating preferences and circumstances, support a broad model of the causes of depression, and encourage them to try different approaches that could facilitate a journey towards recovery. Individual narrative accounts can provide valuable insights into optimal ways to augment ADM treatment or support ADM discontinuation with psychological therapy, and how facilitators and barriers to tapering and discontinuing may interact dynamically within individuals' experiences (Box 1).

Unanswered questions and future research

This work adds to the emerging literature on people's experiences of ADM discontinuation. A next step is to consider whether the same themes emerge in the accounts of people undertaking therapy-supported ADM discontinuation in more naturalistic settings, rather than in the context of a clinical trial, and when using psychological therapies other than MBCT-TS. Additionally, Maund et al. point out that psychological programmes such as MBCT and CBT are relatively resource-intensive.[9] The current study enrolled participants with a history of highly recurrent depression (3 or more episodes). Therapy-supported ADM discontinuation may be most appropriate for those people who are vulnerable to depressive relapse and recurrence, and require the substitution of ADMs with alternative coping skills, whereas others could benefit from simple advice on how to taper ADMs. Likewise, the actual process of ADM tapering, and the typical withdrawal symptoms experienced, are

For peer review only - http://bmjopen.bmj.com/site/about/guidelines.xhtml

both likely to differ according to the type of medication a person is prescribed and the duration of their use. Future research should explore from whom simple tapering advice is enough, and who requires the more intensive support of a programme such as MBCT-TS. In addition, while MBCT-TS may have similarities to other cognitive-behavioural therapies, the commonalities and particularities of people's experiences of tapering and discontinuing ADMs in the context of different psychological therapies is an important focus for future research.

Our findings suggest that people's causal beliefs about depression had an important influence on their responses to therapy-supported discontinuation, but did not examine how people form these beliefs (e.g. the role of the GP, other health professionals, the media, internet). Most consultations about depression will occur in the time-pressured context of primary care. Future work considering how health professionals can most effectively communicate with their patients about the complex aetiology of depression, and understand the how this affects treatment decisions, compliance and outcomes would be valuable. Such communication needs to take into account both short-term goals, both for the clinician and patient (e.g., the clinician's desire to convince a patient in crisis to use ADM), and also the longer-term impact of particular causal models in supporting patients' self-acceptance, agency and recovery.

This work took a qualitative approach in order to understand the breadth of participants' perspectives on their use of MBCT-TS to support ADM tapering, and the range of factors that contributed to the different possible journeys to recovery in the years that followed. To explore whether these factors are significant causal determinants of discontinuation journeys requires further research. Such research could take the factors identified here and test whether they significantly predict ADM tapering/discontinuation in a prospective study. Finally, this research suggests that tapering/discontinuation is not a separate or isolated variable. Participants' accounts clearly demonstrate that the benefits of psychological approaches to support ADM discontinuation and the prevention of depressive relapse cannot be measured by rates of relapse or discontinuation alone. Such research should prioritise the outcome that is most meaningful to patients: their day-to-day functioning and quality of life.

For peer review only - http://bmjopen.bmj.com/site/about/guidelines.xhtml

REFERENCES

1. Kessler R C & Bromet E J. The Epidemiology of Depression Across Cultures. *The Annual Review of Public Health.* 2013;34:119–38. https://doi.org/10.1146/annurev-publhealth-031912-114409
2. World Health Organisation. *Depression and other common mental disorders: Global health estimates.* Geneva: WHO Document Production Services; 2015. https://apps.who.int/iris/bitstream/handle/10665/254610/WHO-MSD-MER-2017.2-eng.pdf (accessed 18 May 2019).
3. Kruijshaar M E, Barendregt J, Vos T, et al. Lifetime prevalence estimates of major depression: An indirect estimation method and a quantification of recall bias. *Eur J Epidemiol.* 2005;20(1):103–111. https://doi.org/10.1007/s10654-004-1009-0
4. National Institute of Health and Care Excellence (NICE). *Depression: The treatment and management of depression in adults (update).* London: Author; 2009. https://www.nice.org.uk/guidance/cg90 (accessed 18 May 2019).
5. Moore M, Yuen H, Dunn N, et al. Explaining the rise in antidepressant prescribing: a descriptive study using the general practice research database. *BMJ.* 2009;339(oct15 2):b3999-b3999. https://doi.org/10.1136/bmj.b3999
6. Maund E, Dewar-Haggarta R, Williams S, et al. Barriers and facilitators to discontinuing antidepressant use: A systematic review and thematic synthesis. *Journal of Affective Disorders*. 2019;245:38-62. https://doi.org/10.1016/j.jad.2018.10.107
7. Bowers H, Williams S, Geraghty A, et al. Helping people discontinue long-term antidepressants: views of health professionals in UK primary care. *BMJ Open*. 2019;9(7):e027837. http://dx.doi.org/10.1136/bmjopen-2018-027837
8. Kuyken W, Warren F C, Taylor R S, et al. Efficacy of mindfulness-based cognitive therapy in prevention of depressive relapse: An individual patient data meta-analysis from randomized trials. *JAMA Psychiatry*. 2016;73(6):565–574. https://doi.org/10.1001/jamapsychiatry.2016.0076
9. Maund E, Stuart B, Moore M, et al. Managing antidepressant discontinuation: a systematic review. *Ann Fam Med.* 2019;17:52-60. https://doi.org/10.1370/afm.2336
10. Bockting C, Klein N, Elgersma H, et al. Effectiveness of preventive cognitive therapy while tapering antidepressants versus maintenance antidepressant treatment versus their combination in prevention of depressive relapse or recurrence (DRD study): a three-group, multicentre, randomised controlled trial. *Lancet Psychiatry*. 2018;5(5):401-410. https://doi.org/10.1016/S2215-0366(18)30100-7
11. Malpass A, Shawa A, Sharp D, et al. ''Medication career'' or ''Moral career''? The two sides of managing antidepressants: A meta-ethnography of patients' experience of antidepressants. *Social Science and Medicine.* 2009;68:154-168. https://doi.org/10.1016/j.socscimed.2008.09.068
12. Bayliss P & Holttum S. Experiences of antidepressant medication and cognitive-behavioural therapy for depression: a grounded theory study. *Psychology and Psychotherapy: Theory, Research and Practice*. 2015;88:317–334. https://doi.org/10.1111/papt.12040
13. Weaver A. Journeys through depression': patients' experiences of transformational change through mindfulness based cognitive therapy (MBCT) and anti-depressant medication (ADM). University of Exeter; 2015. https://ore.exeter.ac.uk/repository/bitstream/handle/10871/18309/WeaverA.pdf?sequence=1&isAllowed=y (accessed 18 May 2019).
14. Kuyken W, Hayes R, Barrett B, et al. Effectiveness and cost-effectiveness of mindfulness-based cognitive therapy compared with maintenance antidepressant treatment in the prevention of depressive relapse or recurrence (PREVENT): A randomised controlled trial. *Lancet*. 2015;386(9988):63–73. https://doi.org/10.1016/S0140-6736(14)62222-4

For peer review only - http://bmjopen.bmj.com/site/about/guidelines.xhtml

15. Kuyken W, Byford S, Byng R, et al. Study protocol for a randomised controlled trial comparing mindfulness-based cognitive therapy with maintenance anti-depressant treatment in the prevention of depressive relapse/recurrence: the PREVENT trial. *Trials*. 2010;11(99):1-10. https://doi.org/10.1186/1745-6215-11-99
16. Kuyken W, Byford S, Byng R, et al. Update to the study protocol for a randomized controlled trial comparing mindfulness-based cognitive therapy with maintenance anti-depressant treatment depressive relapse/recurrence: the PREVENT trial. *Trials.* 2014;15(1). https://doi.org/10.1186/1745-6215-15-217
17. Kuyken W, Hayes R, Barrett B, et al. The effectiveness and cost-effectiveness of mindfulness-based cognitive therapy compared with maintenance antidepressant treatment in the prevention of depressive relapse/recurrence: results of a randomised controlled trial (the PREVENT study). *Health Technol Assess.* 2015;19(73):1-124. https://doi.org/10.3310/hta19730
18. Braun V & Clarke V. Using thematic analysis in psychology. *Qual Res Psychol*. 2006;3(2):77-101. http://dx.doi.org/10.1191/1478088706qp063oa
19. American Psychiatric Association. *Diagnostic and statistical manual of mental disorders* (4th ed., text rev.). Washington, DC: Author; 2000.
20. Segal Z V, Williams J M G, & Teasdale J D. *Mindfulness- based cognitive therapy for depression: A new approach to preventing relapse*. New York: Guilford Press; 2002.
21. Allen M, Bromley A, Kuyken W, et al. Participants' experiences of mindfulness-based cognitive therapy: "It changed me in just about every way possible". *Behav Cogn Psychother*. 2009;37:413-430. https://doi.org/10.1017/S135246580999004X
22. Ridge D, Kokanovic R, Broom A, et al. My dirty little secret: Patient constructions of anti-depressant use and the crisis of legitimacy. *Social Science and Medicine.* 2015;146:53-61. http://dx.doi.org/10.1016/j.socscimed.2015.10.012 0277-9536
23. Tickell A. Patient experiences of discontinuing antidepressant medication with the support of mindfulness-based cognitive therapy. Presented at the International Conference on Mindfulness; 2018; Amsterdam.
24. Farb N, Anderson A, Ravindran A, et al. Prevention of relapse/recurrence in major depressive disorder with either mindfulness-based cognitive therapy or cognitive therapy. *J Consul Clin Psychol*. 2018;86(2):200-204. http://dx.doi.org/10.1037/ccp0000266
25. Kendrick, A. Reviewing long-term anti-depressant use by careful monitoring in everyday practice (REDUCE) programme. 2016 https://europepmc.org/grantfinder/grantdetails?query=pi:%22Kendrick+A%22+gid:%22RP-PG-1214-20004%22+ga:%22DH/NIHR%22 (accessed 18 May 2019).
26. Malpass A, Carel H, Ridd M, et al. Transforming the perceptual situation: a meta-ethnography of qualitative work reporting patients' experiences of mindfulness-based approaches. *Mindfulness*. 2012;3:60-75. https://doi.org/10.1007/s12671-011-0081-2
27. Kuyken W, Brewin C R, Power M J, et al. Causal beliefs about depression in depressed patients, clinical psychologists and lay persons. *Br J Med Psychol*. 1992;65:257-268. https://doi.org/10.1111/j.2044-8341.1992.tb01706.x
28. Engel G. The need for a new medical model: A challenge for biomedical science. *Science*. 1977;196(4286):126-9. Retrieved from: https://www.jstor.org/stable/1743658
29. Moncrieff J, Cohen D, & Porter S. The Psychoactive Effects of Psychiatric Medication: The Elephant in the Room. *J Psychoactive Drugs.* 2013;45(5):409-415. http://doi.org/10.1080/02791072.2013.845328
30. Archer M. S. Routine, reflexivity, and realism. *Sociol Theory*. 2010;28(3):272-303. https://doi.org/10.1111/j.1467-9558.2010.01375.x

For peer review only - http://bmjopen.bmj.com/site/about/guidelines.xhtml

BOX 1: CASE EXAMPLES

Mandy. *Mandy, aged 57, had experienced nine episodes of depression, beginning when she was 32. Following the MBCT-TS course Mandy successfully discontinued her ADM treatment. She did not experience a relapse over the 24-month follow-up period.*

Before the trial, Mandy felt that ADMs helped her to function well. In the past, she had tried tapering, but had always relapsed, so assumed that ADMs would be a part of her life forever. At first, Mandy was nervous, but was willing to try tapering ADMs gradually and with the support of MBCT-TS **(Control: Managing Expectations)**. Mandy's GP was supportive, but reassured her that it was ultimately her decision **(Interactions with GP: Presence and Support)**. During the MBCT-TS course, Mandy said that she learned a different model of depression and developed a better understanding of *"how the mind works"* **(Beliefs about the Causes of Depression: Learning a New Model)**. She felt more confident about tapering, and said that this time it was *"so easy, knowing that I have been given tools to help me through it if needed"*, and found the course *"totally liberating"* as it gave her the chance to take control of her depression, rather than the other way round **(Control: Agency and Responsibility).** She also found it helpful to learn about the possible symptoms of withdrawal, which included mood swings. Mandy realised that the relapses she had experienced when she had tried to taper her ADMs in the past might have been withdrawal symptoms, as opposed to *"real relapses"* **(Tapering Process: Managing Withdrawal Effects)**. At the time of interview, having discontinued ADMs, Mandy still practised what she learned in MBCT-TS and made it part of her daily routine. She accepted that if she ever relapsed, she could use ADMs, but it would only ever be a short-term solution, because she has the MBCT-TS skills as a *"weapon"* to help her manage **(Acceptance: Self-Care)**.

Greta. *Greta, aged 72, had experienced three episodes of depression, beginning when she was 33. Following the MBCT-TS course she discontinued her ADMs but then resumed following a deterioration in mood.*

For peer review only - http://bmjopen.bmj.com/site/about/guidelines.xhtml

Greta was very optimistic about the course because she hated being on ADMs, which gave her unpleasant side effects that interfered with her quality of life **(Control: Managing Expectations)**. At first, Greta said the course made an *"immense difference"* to her, and she described learning how to combat the negative thoughts and feelings she was having **(Beliefs about the Causes of Depression: Learning a New Model)**. The programme left Greta feeling *"so well and positive"* that she decided to taper her ADMs very quickly **(Tapering Process: Pace of Reduction)**, but began to feel her mood dipping. Greta thought this must be a sign that the programme was not working, because she should not feel depressed **(Acceptance: Perspectives on Relapse)**. Greta went to her GP, who did not seem interested in the programme and told her to resume ADMs immediately **(Interactions with GP: Presence and Support).** She was disappointed and felt *"guilty"* that she was not able to use these new skills to keep herself well **(Control: Agency and Responsibility).** She stopped practising mindfulness, although the programme made her remember to appreciate the high points in her day and experience more joy **(Quality of Life: From Coping to Enjoying Life).**

Annie. *Annie, aged 48, had experienced five episodes of depression, beginning when she was 23. Following the MBCT-TS programme, she discontinued her ADMs but then resumed them later.*

Annie felt that ADMs had a positive impact on her life, allowing her to cope day-to-day as a full-time carer for her husband who had a disability. At first, she was very reluctant to try discontinuing ADMs because she believed she might have low levels of serotonin **(Beliefs about the Causes of Depression: Neurochemical Disruption)**. However, the programme taught her a new model of understanding depression **(Beliefs about the Causes of Depression: Learning a New Model)**, which made her feel empowered to practice the psychological techniques **(Control: Agency and Responsibility)**. She started to taper off ADMs, but then her mother died and her husband's health deteriorated, so it was difficult to find time to practice. Her GP advised her it was probably not a good time to discontinue **(Interactions with GP: Presence and Support)**, so she resumed ADMs **(Timing: Feeling Vulnerable).** However, Annie still incorporated the mindfulness exercises into her everyday life, which brought her more joy **(Quality of Life: Experiencing Emotions More Fully)**. She also recognised that it is not her fault when she felt depressed, given how challenging her life was **(Acceptance: Resolving**

For peer review only - http://bmjopen.bmj.com/site/about/guidelines.xhtml

Shame and Denial). Annie felt that the best way to manage her depression was to combine ADMs with mindfulness practices, which gave her more skills to look after herself during difficult times **(Acceptance: Self-Care).** She felt hopeful that one day she would discontinue ADMs, when her life circumstances were more stable **(Timing: Feeling Vulnerable).**

George. *George, aged 37, had experienced ten episodes of depression, beginning when he was 16. Following the MBCT-TS programme he discontinued his ADMs. He experienced a relapse to depression during the 24-month follow-up.*

George was very optimistic about trying an alternative to ADMs, because they made him feel like a *"zombie"*. Having experienced substance misuse issues in the past George had the goal of being totally *"chemical free"* **(Control: Managing Expectations)**. Before the course, George felt he had no control over his depression symptoms, and his mood would deteriorate suddenly without warning. Through practising the mindfulness skills, he described developing more awareness of his emotions and felt he would have the skills to manage them **(Control: Agency and Responsibility).** George said that the best part of taking part in MBCT-TS was meeting other people with depression, which made him feel more accepting of himself **(Acceptance: Resolving Shame and Denial)**. He felt that ADMs had masked his symptoms, whereas MBCT-TS allowed him to explore the problems in his life that were contributing to depression and work through them to make long-term changes **(Control: Agency and Responsibility).** When George relapsed shortly after discontinuing ADMs, he carried on practising MBCT-TS and said that the skills he learned were enough to pull him out of that period of low mood **(Acceptance: Perspectives on Relapse).**

Claire. *Claire, aged 49, had experienced four episodes of depression, beginning when she was 17. Following the MBCT-TS programme, Claire discontinued her ADMs. She relapsed and resumed medication, but subsequently tapered and discontinued again, and was not using ADMs at the time of her follow-up interview.*

At first, Claire was very sceptical about the MBCT-TS programme and thought it might all be *"mumbo jumbo"*. However, she was very keen to come off ADMs, so she approached the programme with an

For peer review only - http://bmjopen.bmj.com/site/about/guidelines.xhtml

open mind and wanted to give it her all **(Control: Managing Expectations)**. As the course progressed, Claire changed her mind and began to *"believe more and more that this might help me"*. MBCT-TS gave her new ways to cope with her feelings, which shocked her because she *"had never took control of my depression before"* **(Control: Agency and Responsibility).** She became very excited and tapered off her medication *"too quickly"* and *"hit a brick wall in a short amount of time. Went straight back in to a deep depression"* **(Tapering Process: Pace of Reduction**). Her doctor was very understanding, and did not push her to do anything, but advised her to go back on ADMs and try to taper off again when she was feeling better **(Interactions with GP: Presence and Support).** He said that she should try tapering them more slowly next time even though she *"wanted to get off them as soon as possible"*. This time, she did *"exactly as she was told"* and did not experience a relapse **(Interactions with GP: Following Advice)**. Claire was very pleased because she said they had always felt that ADMs had *"suppressed"* her and that the person she was when taking ADMs *"wasn't really me"* **(Quality of Life: Experiencing Emotions More Fully)**.

For peer review only - http://bmjopen.bmj.com/site/about/guidelines.xhtml

BOX 2

WHAT IS ALREADY KNOWN ON THIS TOPIC

Many people with a history of depression want to come off their maintenance ADMs but find this challenging, partly due to fears of depressive relapse/recurrence.

Evidence suggests that MBCT may support people to taper and discontinue maintenance ADMs without increasing risk of relapse or recurrence.

However, very little research has examined people's experiences of contemplating, commencing, and completing psychological therapy-supported ADM discontinuation.

WHAT THIS STUDY ADDS

This qualitative study explored how MBCT supported people with a history of recurrent depression to embark upon tapering and discontinuation of their ADMs.

Our findings indicate that people acquired skills to manage their depressive symptoms that could take the place of ADMs, as well as skills to manage the tapering process itself. Participants described this as an effortful process, requiring stable life circumstances, adequate support from medical professionals, and motivation to learn and apply psychological techniques.

Nonetheless, most participants described improvements in quality of life that they attributed to therapy, whether or not they discontinued ADMs, or experienced further relapses/recurrences.

For peer review only - http://bmjopen.bmj.com/site/about/guidelines.xhtml

Statement Concerning Reflexivity

Interviews were conducted by members of the PREVENT trial research team (see Kuyken et al., 2015), following training and using a standard protocol and semi-structured interview schedule. Both male and female interviewers gathered data. Interviewers had knowledge of the participants' treatment journeys prior to conducting their 24-month interviews (from reviewing their files and in some cases as a result of their involvement in earlier waves of data collection). The protocol included interviewers familiarizing themselves with any information about treatment experiences and trajectories of participants, which were held in study records, as part of the interview preparation process. Researchers did not know participants prior to their entry to the trial, and had no association with them outside the context of the trial and associated research assessments.

All/Some interviewers had undertaken mindfulness training, acting as participant observers in MBCT courses, or in other contexts. This personal knowledge enabled them to understand the nuances in participants' descriptions of their experiences, for example participants' references to particular mindfulness practices or exercises, and to respond with confidence. Some interviewers had positive personal experiences of mindfulness whereas others held more neutral attitudes. None were aware of the main trial outcomes at the time the interviews were conducted and all were encouraged to adopt an open minded and curious attitude, with no preconceptions about whether MBCT-TS had, or had not, supported participants in their treatment journeys. Despite this, it should be acknowledged that some interviewers may have held implicit biases or expectations regarding treatment effects. Likewise, participants understood the association of interviewers with the primary trial. Thus whilst participants were encouraged to speak freely and honestly about their experiences, it is possible that their responses were influenced by the perceived allegiance of the researchers to

For peer review only

the intervention being explored, and that they may have felt a sense of obligation to make positive comments about MBCT-TS.

The researchers conducting data analysis, AT (BA Hons, Postgraduate Research Assistant) and CC (DPhil, Senior Research Fellow), although not involved in the PREVENT trial, had a knowledge of the programme under investigation. AT has undertaken mindfulness training in other contexts, and is familiar with the MBCT curriculum. CC has significant prior experience as a participant-observer in MBCT classes for people with recurrent depression. JR (DPhil, Lecturer) is an experienced qualitative researcher who has theoretical knowledge of mindfulness-based approaches. AT, CC and JR have all worked previously on studies exploring mindfulness-based programmes in different contexts and AT and CC were employed on a research grant exploring the effectiveness of mindfulness-based programmes at the time this work was conducted. AT, CC, and JR were aware of the main outcomes of the PREVENT trial (no superiority of MBCT-TS over maintenance antidepressants) at the time the qualitative analysis commenced, and approached the data with an assumption of overall equipoise between the two approaches, that was nevertheless likely to concealed marked individual differences in response. WK (PhD, DClinPsy) was the Principal Investigator on the PREVENT trial and is a mindfulness trainer and practitioner. He was not involved in directly teaching mindfulness to any of the participants in the PREVENT trial, but did supervise the mindfulness teachers who taught the MBCT-TS trial classes. He had no personal knowledge of the individual participants and their treatment journeys.

For peer review only

PREVENT: End of Trial Interviews
Guidance for Interviewers

Aims

Researchers will conduct interviews with 42 purposively sampled participants in the MBCT arm of the trial at the end of the trial. The research topic we are targeting here is:
Participants' experiences during the follow-up period of the PREVENT trial of use of ADMs and MBCT-based techniques in relation to each other, during periods of wellness, depressive relapse, and transitions between these two.

Interview Preparation

Before conducting any of the interviews researchers read the key qualitative papers (Malpass et al., 2012; Allen et al., 2009). Before each interview researcher to read the participant's file to (re-) familiarise themselves with participant characteristics (e.g., relationship status) and obtain timeline populated with info on borderline or actual relapses and ADM usage. They should also review, and have to hand in the interview, the end of treatment and 24-month feedback booklets to get a sense of the profile of each participant during the follow-up period in terms of the following variables: full or partial attendance of MBCT course; use of mindfulness techniques; use of ADMs; and any significant life events. This will shape the interview in terms of: i) which sub-sections of the interview are relevant; ii) issues for particular participants that may need to be probed in more detail.
Make sure you are as clear as possible about which sections of the interview and questions are relevant to your respondent BEFORE YOU START. Where possible, **choose specific/representative/salient episodes or junctures** in timeline for targeted probing using the schedule (possibly match them onto wellness, wobbles, and depressive sections). **Print out two copies of timeline**, one for yourself and one for the interviewee to draw upon.

Conducting the Interview

The interview is semi-structured. In each section, questions and follow-up questions are suggested. However, researchers should use their judgment in drawing out participants in relation to the research topic. Interviewers ask open-ended questions and follow participants' "leads" while keeping in mind the research question. The aim is to enable participants to give their "story" around the main topics of investigation.
Researchers should be strategic in use of time in the interview to ensure the topic guide/respondent covers material that answers the research questions; especially in Section 1, it is important to keep the interview focused. If the interviewer feels respondents are being unclear or opaque help the respondent unpack what they mean or agree with the participant that the issue is unclear. Interviewers should ensure *that all the interview questions that are relevant to the participants' particular profile are covered.* Consider a temporal order to do the interview allocating/planning pre-set time windows for each consecutive episode/junction so as not to exceed a **maximum of 60 minutes** for the overall interview.

Participants may feel the need to please researchers or give the "right" answers. This is particularly likely around ADM tapering where the trial has consistently communicated that we want participants to taper and discontinue their ADM. It is important that researchers communicate explicitly and non-

For peer review only - http://bmjopen.bmj.com/site/about/guidelines.xhtml

verbally that there are no right and wrong answers to any of the questions and we want to know people's experiences in relation to MBCT and ADM.

Use of particular terms (e.g., "wobble," "relapse") can be adapted in the light of language used by participants. Follow-up questions should be asked for clarification and elaboration, *and this should be driven by the research aim for each section of the interview*. As far as possible interviewers should cover all the suggested topics and questions and behave with the minimum of variation between interviews. Instructions to interviewers and suggested wordings for introductions to each section are given in italics below. The key issues for each question are in bold.

Interview Opening / Introduction

Open the interview with something like:

"Thank you for agreeing to do this interview with me. The interview is about hearing from you how things have been in the trial and checking that we have your story right. It will be collaborative and semi-structured which means I have some topics I'd like to cover, but the questions will be quite open so as to give you, as an expert of your own lived experience, a chance to express your own views and tell me about your experiences and what seems important to you. Everyone will have had different experiences and we are interested in hearing about these from your point of view – both the good and the not-so-good experiences – so that we can learn about and develop our treatments further. Please tell us exactly how you feel!

Just to let you know that I am un-blinded and know that you have participated in the MBCT group, so it is ok to talk freely about this. I would like to record this interview, and the recording will be transcribed word for word and analysed as part of the research for the PREVENT trial. All identifying information will be removed at this point. Your name will never appear on the transcription or any other documents or files that result from this interview (such as the audio file). Do you have any questions before we begin?"

Section 1: Overview of the follow-up period using the timeline

The aim of this section is to obtain a brief overview of how the participant has experienced the follow-up period in terms of three areas: 1. Periods of wellness and depression; 2. Antidepressant medication; 3. Life events. Subsequent interview sections follow up on each of these in more detail and the profile of experiences here can be used to tailor subsequent interview questions. Information on the first two areas should be available in advance and summarised on the prepared timeline. Information on life events might be found in the research files and database but will need to be obtained here. Preparation by thoroughly reading the file, and possibly even some of the audio recordings, in advance will help a lot here.

Use the timeline in whatever way feels most comfortable and guided by the respondent's preference. The participant can add life events him/herself; or the researcher can do this; or it can simply be used as a guide. Show the timeline and say something like:

"I'd like to start with a brief overview of how life has been for you in the trial. In preparing for our meeting today, we have put together this timeline. It summarises the information you have given us as part of the research study about any periods of depression or wobbles, and your use of antidepressant medication during the trial. It also includes the date of your most recent episode of depression before taking part in the trial, based upon what you told us during your first assessment. I've also

For peer review only - http://bmjopen.bmj.com/site/about/guidelines.xhtml

looked through your comments in the Feedback Booklets you completed one month after the end of the mindfulness course and again recently. These were very helpful and I'll use some of what you wrote there to guide what we talk about today."

Interview Question	Probes/Examples/Directions
1.1. Do you think the information we have here on the timeline looks about right? Is there anything you would like to change?	If we know from previous assessments that the person has had a significant life event ensure this is acknowledged here. Mark – or get the respondent to mark – any life events on the time-line / graph.
1.2. Have there been any important events in your life during your time in the trial that have affected you either positively or negatively that we can add to the timeline?	Keep the discussion focused and brief to allow as much time for later sections as possible.
1.3. Can we check that I have your use of antidepressant medication right these last two years?	Work through timeline for any tapering/discontinuation, dosage, resumption etc.
1.4. How have things been between the last time we spoke and today?	Informally extend timeline with relevant depressive episodes/ADM use/life events up until current interview date; Where necessary, establish current symptomatic status (informally) and make sure they are ok to continue.

"The remaining questions in this interview will focus upon your experiences with the aid of this timeline."

Keep the time-line / graph in view to refer back to / use further in later parts of the interview. Use the timeline to keep the interview contained with regard to the different episodes/junctures identified prior to interview. Allow and encourage the participant to use a pen to put down details in their copy of the timeline.

Section 2: Questions on wellness (for all)

Comment on Section 2-4: Particularly in the wobbles section it is important to go for depth rather than breadth with regards to potential issues around these time points (e.g. what happened in days before, relationships, sleep etc.). As a general principle, interviewers should encourage interviewees to focus on prototypical or most memorable junctures in timeline. In order to keep interview contained, agree on a timeframe for each episode/juncture and keep questions focused on this.

"I'd like to ask about any times when you were well during the trial – when you weren't feeling low or experiencing an episode of depression"

For peer review only - http://bmjopen.bmj.com/site/about/guidelines.xhtml

Interview Question	Probes/Examples/Directions
2.1. Has anything from the mindfulness course played a part in staying well during the trial? If so, can you describe how?	Examples: any techniques, ideas; response plan<br>Prompts: How / why / why not?; Therapist/Group/Researcher Role
2.2 Did your use of antidepressants play a part in staying well during the trial? If so, can you describe how?	Prompts: How / why / why not? ; GP Role
2.3 What about the combination of xxx from the mindfulness course and use of antidepressants in periods of wellness?	[ask this additional question if respondent says that both MBCT and ADM have had some value in questions 2.2 and 2.3., otherwise skip]

Section 3: Question on wobbles / early signs of depression (for all)

"I'd like to ask about any times when you were well, but you felt yourself starting to 'wobble' or feel low. So any times when you might have felt that your mood was dipping or you were starting to have more of the negative thoughts that were around when you were depressed"

Interview Question	Probes/Examples/Directions
3.1. How did these experiences of 'wobbles' or starting to feel low compare to previous experiences of wobbles?	Use timeline to go right into the situation shortly before, during and directly after episode.<br>Probe: What happened, how was it different? What did you do? How did you get out of it?
3.2. Was anything from the mindfulness course useful at the time of wobbling or when starting to feel low during the trial? If so, can you describe how?	Probe: before/during/after wobble<br>Examples: any techniques, ideas; response plan, self-compassion etc.<br>Prompts: How / why / why not?
3.3 Did your use of antidepressants during the trial play a part in wobbling or starting to feel low during the trial? If so, can you describe how?	Prompts: How / why / why not?
3.4 What about the combination of xxx from the mindfulness course and use of antidepressants in periods of wobbling or when starting to feel low?	[ask this additional question if respondent says that both MBCT and ADM have had some value, otherwise skip as appropriate]

For peer review only - http://bmjopen.bmj.com/site/about/guidelines.xhtml

Section 4: Experiences of depressive relapse (for those who have relapsed)

Use the timeline to guide question choices in relation to experiences of relapse.

Say: "I'd like to ask you about your experiences this / these episode of depression..."

Interview Question	Probes/Examples/Directions
4.1. How did this episode of depression compare to previous episodes of depression?	Use timeline to take them right into the situation shortly before, during and directly after episode. Probe: What happened, how was it different? What did you do? How did you get out of it?
4.2. Was anything from the mindfulness course useful at the time of depression? If so, can you describe how?	Examples: any techniques, ideas; response plan<br>Prompts: How / why / why not?
4.3. Did your use of antidepressants during the trial play a part in this episode of depression? If so, can you describe how?	Prompts: How / why / why not?
4.4 What about the combination of xxx from the mindfulness course and use of antidepressants in periods of depression?	[ask this additional question if respondent says that both MBCT and ADM have had some value, otherwise skip as appropriate]

Section 5: MBCT and ADMs combined

"I'd like to end with some open questions about your experiences of taking part in this study."

For sakes of time keeping and for keeping this section contained it is critical how the questions are set up. Encourage people to step back from and reflect critically on their own experience rather than letting them share their raw experience. Use formulations like the following: *'I am sure that this was an intensive time for you. If you took yourself away from the experience today and were to reflect on this, what may I ask are the key elements/thoughts that you would have with regards to staying well etc.?'*

Interview Question	Probes/Examples/Directions
5.1. Has taking part in the trial changed the way you think about depression? If so, could you tell me how?	Probe: How/why /why not?<br>Probe: Has it changed the way you think about the causes/consequences of depression? Role of GP.
5.2. Has taking part in the trial changed how you think about yourself? If so, could you tell me how?	Probe: how identity was before the trial if person describes a sense of change.<br>Examples: Role of group, immediate and wider social

For peer review only - http://bmjopen.bmj.com/site/about/guidelines.xhtml

	environment
5.3. Now that you have had experiences of both mindfulness and antidepressants, what do you think of each of them as treatments for depression?	Probes: do you favour one over the other? In combination?<br>At different points e.g. in wellness, wobbles and depressive episodes?<br>For prevention? For recovery?
5.4. Apart from depression, has taking part in the trial had any impact on other psychological or physical health problems you may have?	Examples:<br>Anxieties/phobias, Chronic health conditions like pain, diabetes etc.

Section 6: Ending

"I've covered all the questions we have, but before we end, is there anything you would like to add?"

Interview Question	Probes/Examples/Directions
6.1. …about any of the topics we have discussed?	
6.2. …about any of your experiences of depression, mindfulness or antidepressant medication that we have not talked about?	
6.3. …about anything else you think is relevant to this project on MBCT, antidepressants, and depression?	

End by thanking the respondent for their time and for sharing their views and experiences. Reiterate how valuable this is for research trying to develop treatments for depression. Inform them about further dissemination of PREVENT findings.

For peer review only - http://bmjopen.bmj.com/site/about/guidelines.xhtml

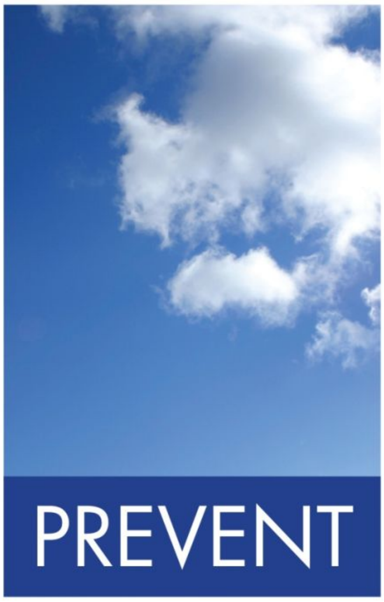

1-month Feedback Booklet

For peer review only

UNIVERSITY OF EXETER

telephone: 01392 726102 email: prevent@exeter.ac.uk web: www.prevent-southwest.org.uk
ISRCTN26666654 EudraCT number: 2009-012428-10
In collaboration with The University of Bristol, King's College London and the Medical Research Council Cognition and Brain Sciences Unit. Funded by National Institute for Health Research HTA

NHS

For peer review only - http://bmjopen.bmj.com/site/about/guidelines.xhtml

PREVENT Feedback Booklet

As researchers, we are very aware that we are working with people. In this case, a specific group of people who have all suffered from depression at times in their lives. We are careful to never lose sight of the fact that each person is also an individual with their own personality, feelings, and experiences.

One of our aims is to find out about the thoughts, feelings and experiences of the people taking part in this research project at two time points: 4-6 weeks after your final session of MBCT, and again at the end of your involvement in the project. We want to hear your individual views because we think that we can learn a lot from them.

Please take time to think about the questions in this booklet and answer them in your own words. It might be useful to read through the questions before writing your answers, in order to become familiar with them. Tell us exactly what you think! We won't be offended if you didn't like some parts, or even all, of the project. If you found some or all of it useful, helpful or enjoyable, then tell us that too, and please tell us why.

There are 11 questions in the booklet. Write as much, or as little as you like. If you feel that you have more to say than there is space for then please call us on 01392 726102 and we'll provide you with a second booklet.

As we have said in the past, we guarantee your privacy and confidentiality. Nothing that you say here will be published or printed in any way that could identify you, and no unauthorised person will ever read this booklet.

Once you have completed the booklet, please return it to us in the envelope provided. The envelope has a freepost sticker on it, so you do not need to pay for postage.

Finally, many thanks for the effort you have put in to this project so far. Hopefully our research will enable us to develop new and better ways to help people stay well after depression.

The PREVENT Team

PREVENT Trial ID: _________

PREVENT Feedback Booklet 1.1 v2. 02.10.10

For peer review only

Reducing your antidepressants

The first set of questions aims to explore your **thoughts** towards, and **experiences** of, taking and reducing your antidepressant medication.

1. Please describe your experiences of using antidepressants before taking part in the MBCT course. For example, did you feel that antidepressants were having a positive impact upon your day-to-day life and functioning? Did you experience any difficulties taking antidepressants in the past? Please explain why.

....

....

....

....

....

....

2. During the MBCT course you were asked to try to reduce your use of antidepressants. Please can you describe what your **thoughts** were towards reducing your antidepressants directly before the MBCT course started and again during the MBCT course when you were asked to try reducing them?

Thoughts about reducing antidepressants *before* the MBCT course:

....

....

....

....

....

....

Thoughts about reducing antidepressants *during* the MBCT course:

....

....

....

....

....

....

For peer review only

For peer review only - http://bmjopen.bmj.com/site/about/guidelines.xhtml

2. Can you please describe your **experiences** of reducing your use of antidepressants during the MBCT course up until this point now?
For example, have you started to reduce your antidepressants? (If YES, please continue below. If NO, please go to Question 3)

When did you start reducing your antidepressants? (Please be as specific as possible about the date)

....

What positive impact, if any, has reducing your antidepressants had on your day-to-day life and functioning?

....
....
....
....

What difficulties, if any, did you experience when reducing your antidepressants?

....
....
....
....
....
....

Did you do anything in particular to overcome these difficulties?

....
....
....
....
....
....

For peer review only - http://bmjopen.bmj.com/site/about/guidelines.xhtml

If you have tried reducing your antidepressants in the past, please describe if your experiences of reducing your antidepressants recently has been different, and if so, in what ways. If there has been no difference, please let us know.

....
....
....
....
....
....

3. It would be useful for us to know more about why people who take part in this research study do not reduce their antidepressants. Please could you describe why you have not reduced your antidepressant medication at this stage in the research study?

....
....
....
....
....
....
....
....

For peer review only

Taking part in MBCT

The questions in this section aim to find out more about your experiences of taking part in MBCT, and continuing the MBCT exercises after the course.

4. Out of the 8 week course, how many MBCT sessions did you attend?

 .... out of 8 sessions.

5. Did you attend the first MBCT follow-up session? YES / NO

6. What, if any, MBCT exercises are you still practicing? Please tick the ones you are still practicing and in the space provided write how often you practice (e.g. per week or per month).

☐ None

☐ Body scan

....

☐ Sitting meditation

....

☐ 10-min sit

....

☐ 20-min sit

....

☐ Silence with bells

....

☐ Breathing space (regular, 3 times a day)

....

☐ Breathing space (‘coping’ space)

....

☐ Mindful walking

....

☐ Stretch and breath

....

☐ Mindful activities (please specify what, and how often per week or month)

....

☐ Other (please specify)

....

....

For peer review only - http://bmjopen.bmj.com/site/about/guidelines.xhtml

7. Please describe anything that you may have found **helpful** about taking part in the MBCT course and practicing the mindfulness meditation exercises, **and why**.

Specific aspects of the MBCT course you found **helpful**, and why:

....
....
....
....
....
....
....
....
....

Specific aspects of the meditation practices and teachings that you found **helpful**, and why:

....
....
....
....
....
....
....
....
....

For peer review only

For peer review only - http://bmjopen.bmj.com/site/about/guidelines.xhtml

8. Please describe anything that you may have found **unhelpful** about taking part in the MBCT course and practicing the mindfulness meditation exercises, **and why**.

Specific aspects of the MBCT course that you found **unhelpful**, and why:

....
....
....
....
....
....
....
....
....

Specific aspects of the MBCT practices and teachings that you found **unhelpful**, and why:

....
....
....
....
....
....
....
....
....

For peer review only

The Impact of MBCT

The final two questions relate to the **impact**, if any, that MBCT may have had on you.

9. Have you benefitted from the MBCT course and practicing the MBCT exercises? If so, in what ways? (We are interested to hear about benefits not only in how you feel, but in any other areas of your life). If there have been no benefits or that some things are worse now, please tell us about these.

....
....
....
....
....
....
....
....
....
....
....
....
....

10. Do you feel that MBCT **works for you**? If so, why do you think it works? If it doesn't work for you, please tell us why you think it doesn't work.

....
....
....
....
....
....
....
....
....
....
....

For peer review only

Any other comments:

11. Do you have any other comments that you would like to share about your experiences of taking part in this project?

....
....
....
....
....
....
....
....
....
....
....
....
....
....

For peer review only

For peer review only - http://bmjopen.bmj.com/site/about/guidelines.xhtml

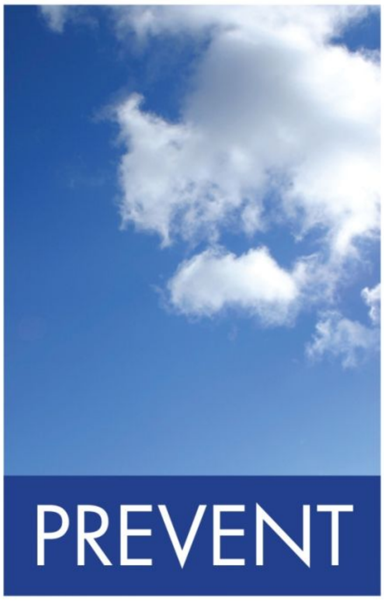

24-month Feedback Booklet

For peer review only

UNIVERSITY OF EXETER

telephone: 01392 726102 email: prevent@exeter.ac.uk web: www.prevent-southwest.org.uk
ISRCTN26666654 EudraCT number: 2009-012428-10
In collaboration with The University of Bristol, King's College London and the Medical Research Council Cognition and Brain Sciences Unit. Funded by National Institute for Health Research HTA

NHS

For peer review only - http://bmjopen.bmj.com/site/about/guidelines.xhtml

PREVENT 24-month Feedback Booklet

As researchers, we are very aware that we are working with people. In this case, a specific group of people who have all suffered from depression at times in their lives. We are careful to never lose sight of the fact that each person is also an individual with their own personality, feelings, and experiences.

One of our aims is to find out about the thoughts, feelings and experiences of the people taking part in this research project. We asked you to complete a feedback booklet in the weeks after attending MBCT, and we would be really grateful if you could now complete a second feedback booklet to explore your experiences of taking part in the project over the past two years. We want to hear your individual views because we think that we can learn a lot from them.

Please take time to think about the questions in this booklet and answer them in your own words. It might be useful to read through the questions before writing your answers, in order to become familiar with them. Tell us exactly what you think! We won't be offended if you didn't like some parts, or even all, of the project. If you found some or all of it useful, helpful or enjoyable, then tell us that too, and please tell us why.

There are 4 parts to this booklet and 27 questions in total, although not all of them will apply to you. Please read the instructions throughout the booklet to check which questions are relevant to you.

Please write as much, or as little, as you like. If you feel that you have more to say than there is space for then please use the back sheet to write any additional feedback.

As we have said in the past, we guarantee your privacy and confidentiality. Nothing that you say here will be published or printed in any way that could identify you, and no unauthorised person will ever read this booklet.

Once you have completed the booklet please give it to your PREVENT researcher at the 24-month assessment.

Finally, many thanks for the effort you have put in to this project. Hopefully our research will enable us to develop new and better ways to help people stay well after depression.

The PREVENT Team

PREVENT Trial ID: __________

For peer review only - http://bmjopen.bmj.com/site/about/guidelines.xhtml

Part I: Experiences of Reunions

Following your MBCT course you were invited to attend four MBCT reunion sessions.

1. How many did you attend? (Please circle)

None 1 2 3 4

2. Were there any specific reasons for attending this number of reunion sessions?

....

....

....

....

....

....

....

If you didn't attend any reunions, please go to Part II on page 4.

If you attended some or all reunion sessions, please continue below.

3. Please describe what you found helpful and / or unhelpful about the reunion sessions?

....

....

....

....

....

....

....

For peer review only

4. Have you used any other support to maintain your mindfulness practice (for example, keeping in contact with anyone from your MBCT course, or joining local meditation groups)? Please describe how this has been helpful and / or unhelpful and why.

....

....

....

....

....

....

....

For peer review only

Part II: Doing Mindfulness Practices

Which of the following statements best describes your use of mindfulness techniques (both formal practices and other mindful activities) over the past two years? Please tick one box and then follow the instructions for which question to complete next:

☐ I've used mindfulness techniques regularly over the last 2 years (please continue to Question 5)

☐ I used mindfulness techniques for a while after the MBCT course but my practice has tailed off and I no longer practice (please continue to Question 5)

☐ I've used mindfulness techniques off and on during the last 2 years (please continue to Question 5)

☐ I have not used mindfulness techniques at all since the MBCT course (please go to Question 7)

For peer review only

5. Please complete the two tables below to tell us which techniques you have practiced over the past two years and what you are currently practicing.

The following table provides a list of mindfulness practices from the MBCT course.

	Currently	Over the past two years
**Example: Body Scan**	*2* times per *week*	*Regularly for about 3 months after the course but then stopped. Restarted 2 months ago.*
**Body Scan**	times per	
**Sitting Meditation**	times per	
**10-minute sit**	times per	
**20-minute sit**	times per	
**Silence with Bells**	times per	
**Breathing Space (Regular, e.g. 3 times a day)**	times per	
**Breathing Space ('Coping' space)**	times per	
**Mindful Walking**	times per	
**Mindful Movement / Yoga**	times per	

For peer review only

The following table gives you space to record any mindful activities you've practiced.

Mindful Activities	Currently	Over the past two years
E.g., being mindful when showering	*Every morning*	*Every morning, although I stopped for roughly 3 months about a year ago.*

6. Have you adapted any of the mindfulness exercises you learned in the MBCT course to suit you? If so, how? Why did you make these changes?

....
....
....
....
....
....
....

7. If you do not currently practice any mindfulness techniques, please describe why.

....
....
....
....
....
....

For peer review only

Part III: The impact of MBCT and mindfulness practice

8. Do you feel that MBCT "works" for you? If so, how do you think it works? If you would like to, please use examples from your life.

....
....
....
....
....
....
....

9. What is the single most important thing that you've learned through attending the MBCT course or practicing mindfulness techniques? Please explain your answer.

....
....
....
....
....
....
....

10. MBCT may not be the right treatment for everyone. If you feel that MBCT was not right for you, please tell us why.

....
....
....
....
....
....
....

For peer review only

11. What changes, if any, would you make to the MBCT course and / or mindfulness exercises to make them more suitable for you?

For peer review only

Part IV: Taking antidepressants

We are interested in learning more about your experiences of taking and reducing antidepressants over the past two years.

12. Have your thoughts about reducing your antidepressants changed over the past two years? If so, in what ways?

....
....
....
....
....
....

13. Does taking antidepressant medication "work" for you? If so, please tell us why. If taking antidepressants does not "work" for you, please also tell us why. If you would like to, please use examples from your life.

....
....
....
....
....
....

14. What role has your GP had in your use of antidepressant medication over the past two years? How have you found this?

....
....
....
....
....
....

For peer review only

15. Has anyone else been involved in your use of antidepressant medication over the past two years? (E.g., psychiatrist, family member, another practitioner). If so, in what ways?

....
....
....
....
....
....

Which of the following best describes your use of antidepressants over the last two years? Please tick one box and follow the instructions for which questions to complete next.

☐ I have continued using antidepressants and have not reduced my use at any point over the past two years that I've been involved in this study. (Tick this box even if you have changed to a different antidepressant, or increased the dosage of your antidepressant.)

Please answer the questions in the ◆ box on the following page.

☐ I am in the process of reducing my antidepressants, or have reduced/stopped my use of antidepressants at some point over the past two years that I've been involved in this study. (Tick this box even if you restarted or increased your medication at a later date.)

Please answer the questions in the ● box on page 12

For peer review only

16. In your day-to-day life and functioning over the past two years, have you experienced any positive effects of continuing to use antidepressants? If so, please describe these.

....

....

....

....

....

17. In your day-to-day life and functioning over the past two years, have you experienced any negative effects or difficulties in continuing to use antidepressants? If so, please describe these.

....

....

....

....

....

18. Have you done anything specific to overcome these negative effects or difficulties? If so, what have you done?

....

....

....

....

....

19. Please describe why you haven't reduced your antidepressant medication over the past two years.

....

....

....

....

....

Please now go to Question 27 on page 11.

20. Has reducing your use of antidepressants had any positive effects on your day-to-day life and functioning over the past two years? If so, please describe how.

21. Have you experienced any negative effects or difficulties associated with reducing your antidepressants over the past two years? If so, please describe them.

22. Have you done anything specific to overcome these negative effects or difficulties? If so, what have you done?

23. Has practicing mindfulness techniques helped with these negative effects or difficulties? If so, in what ways?

Continued...

Continued...

24. Have you stopped using antidepressant medication completely, now or at any point in the last two years? Please describe your experiences of this.

....

....

....

....

....

25. Have you tried reducing your antidepressants at any point before this study? If so, please tell us of any ways that reducing your antidepressants over the past two years been different? If there has been no difference, why do you think this is the case?

....

....

....

....

....

26. Have you reduced or stopped your antidepressants over the past two years and then restarted or increased them again? If so, please tell us about this, and how you felt about this?

....

....

....

....

....

Please go to Question 27 on the following page.

Any other comments?

27. **Thank you for taking the time to complete this booklet - your participation is greatly appreciated!** If you have any other comments that you would like to share about your experiences of taking part in this project which did not fit into any of the answer spaces above, please use the space below.

For peer review only

BMJ Open

RECOVERY FROM RECURRENT DEPRESSION WITH MINDFULNESS-BASED COGNITIVE THERAPY AND ANTIDEPRESSANTS: A QUALITATIVE STUDY WITH ILLUSTRATIVE CASE STUDIES

Journal:	*BMJ Open*
Manuscript ID	bmjopen-2019-033892.R1
Article Type:	Research
Date Submitted by the Author:	08-Jan-2020

Complete List of Authors:	Tickell, Alice; University of Oxford, Psychiatry<br>Byng, Richard; University of Plymouth, Community and Primary Care Research Group<br>Crane, Catherine; University of Oxford, Psychiatry<br>Gradinger, Felix; Plymouth University, Community and Primary Care Research Group<br>Hayes, Rachel; University of Exeter, College of Medicine and Health<br>Robson, James; University of Oxford, Education<br>Cardy, Jessica; Oxford University, Oxford Institute of Clinical Psychology Training<br>Weaver, Alice; Devon Partnership NHS Trust<br>Morant, Nicola; University College London, Psychiatry<br>Kuyken, Willem; University of Oxford, Psychiatry

**Primary Subject Heading**:	Mental health
Secondary Subject Heading:	Health services research
Keywords:	Depression & mood disorders < PSYCHIATRY, psychological therapy, antidepressants

SCHOLARONE™ Manuscripts

I, the Submitting Author has the right to grant and does grant on behalf of all authors of the Work (as defined in the below author licence), an exclusive licence and/or a non-exclusive licence for contributions from authors who are: i) UK Crown employees; ii) where BMJ has agreed a CC-BY licence shall apply, and/or iii) in accordance with the terms applicable for US Federal Government officers or employees acting as part of their official duties; on a worldwide, perpetual, irrevocable, royalty-free basis to BMJ Publishing Group Ltd ("BMJ") its licensees and where the relevant Journal is co-owned by BMJ to the co-owners of the Journal, to publish the Work in this journal and any other BMJ products and to exploit all rights, as set out in our licence.

The Submitting Author accepts and understands that any supply made under these terms is made by BMJ to the Submitting Author unless you are acting as an employee on behalf of your employer or a postgraduate student of an affiliated institution which is paying any applicable article publishing charge ("APC") for Open Access articles. Where the Submitting Author wishes to make the Work available on an Open Access basis (and intends to pay the relevant APC), the terms of reuse of such Open Access shall be governed by a Creative Commons licence – details of these licences and which Creative Commons licence will apply to this Work are set out in our licence referred to above.

Other than as permitted in any relevant BMJ Author's Self Archiving Policies, I confirm this Work has not been accepted for publication elsewhere, is not being considered for publication elsewhere and does not duplicate material already published. I confirm all authors consent to publication of this Work and authorise the granting of this licence.

For peer review only

RECOVERY FROM RECURRENT DEPRESSION WITH MINDFULNESS-BASED COGNITIVE THERAPY AND ANTIDEPRESSANTS: A QUALITATIVE STUDY WITH ILLUSTRATIVE CASE STUDIES

Alice Tickell, Department of Psychiatry, University of Oxford, alice.tickell@psych.ox.ac.uk

Richard Byng, Community and Primary Care Research Group, University of Plymouth, richard.byng@plymouth.ac.uk

Catherine Crane, Department of Psychiatry, University of Oxford, catherine.crane@psych.ox.ac.uk

Felix Gradinger, Community and Primary Care Research Group, University of Plymouth, felix.gradinger@plymouth.ac.uk

Rachel Hayes, College of Medicine and Health, University of Exeter, r.a.hayes@exeter.ac.uk

James Robson, Department of Education, University of Oxford, james.robson@education.ox.ac.uk

Jessica Cardy, Oxford Institute of Clinical Psychology Training, University of Oxford, jessica.cardy@hmc.ox.ac.uk

Alice Weaver, Devon Partnership NHS Trust, a.weaver@exeter.ac.uk

Nicola Morant, Division of Psychiatry, University College London, n.morant@ucl.ac.uk

Willem Kuyken, Department of Psychiatry, University of Oxford, willem.kuyken@psych.ox.ac.uk

Correspondence concerning the article should be addressed to Professor Willem Kuyken, Department of Psychiatry, Warneford Hospital, Oxford, OX3 7JX.

Email: willem.kuyken@psych.ox.ac.uk

Word Count 7900 (excluding title page, abstract, references, figures, and tables)

For peer review only

Contributor Statement

WK, RB & NM were responsible for the PREVENT trial protocol and secured the study funding. NM designed the over-arching qualitative process study to elicit service users' experiences of treatment, with input from RB, FG, RH, JC, and WK. Interviews were conducted by FG and AW, supervised by NM. CC,WK, JR, and AT developed the analytical strategy and protocol for the study reported here, and AT conducted the bulk of the analysis, with input from other members of the analytical team. AT, CC, and WK drafted the manuscript. All other authors read the manuscript, revised it for significant intellectual content, and approved the final manuscript. As Chief Investigator, WK had overall responsibility for the parent trial within which this study was embedded. The University of Exeter held responsibility for the parent trial and this work. WK is guarantor and corresponding author for the study.

Competing Interest Statement

All authors have completed the ICMJE uniform disclosure form at www.icmje.org/coi_disclosure.pdf and declare the following: AT reports that she is employed as a researcher at the University of Oxford Mindfulness Centre, conducting research on mindfulness-based interventions. However, she receives no additional remuneration for teaching or publications related to mindfulness-based approaches. RB has written about his concerns about the increases in prescribing of antidepressants. CC reports that she is employed as a researcher at the University of Oxford Mindfulness Centre, conducting research on mindfulness-based interventions. However, she receives no additional remuneration for teaching or publications related to mindfulness-based approaches. WK is the Director of the Oxford Mindfulness Centre. He receives no payments for training workshops and presentations related to MBCT; such payments are made directly to the Oxford Mindfulness Foundation, a charitable trust that supports the work of the Oxford Mindfulness Centre. He receives royalties for his book Mindfulness: Ancient wisdom meets modern psychology published by Guilford Press. WK was until 2015 an unpaid Director of the Mindfulness Network Community Interest Company and gave evidence to the UK Mindfulness All Party Parliamentary Group. FG, RH, JR, JC, AW, and NM have nothing to disclose.

For peer review only

Transparency Statement

WK confirms that the manuscript is an honest, accurate, and transparent account of the study being reported; that no important aspects of the study have been omitted; and that any discrepancies from the study as originally planned have been explained. He attests that all listed authors meet authorship criteria and that no others meeting the criteria have been omitted.

Funding Statement

The PREVENT trial was funded by the National Institute for Health Research Health Technology Assessment (NIHR HTA) Programme [08/56/01] and was published in full in Health Technology Assessment. The views and opinions expressed therein are those of the authors and do not necessarily reflect those of the HTA programme, NIHR, NHS or the Department of Health and Social Care. A Trial Steering Committee (TSC), Data and Ethics Monitoring Committee (DMEC), the UK Mental Health Research Network (MHRN), the Primary Care Research Network (PCRN), and the Comprehensive Local Research Network (CLRN) all provided support to the project. WK, CC and AT are supported by the Wellcome Trust [107496/Z/15/Z]. RB received funding from NIHR Collaboration for Leadership in Applied Health Research and Care South West Peninsula.

The funders had no role in the design of the study, in the collection, analysis and interpretation of the data, in the writing of the report, or in the decision to submit the article for publication. All authors are independent of the funders, had full access to all of the data in the study, and can take responsibility for the integrity of the data and the accuracy of the data analysis.

License

The Corresponding Author has the right to grant on behalf of all authors and does grant on behalf of all authors, a worldwide licence to the Publishers and its licensees in perpetuity, in all forms, formats and media (whether known now or created in the future), to i) publish, reproduce, distribute, display and store the Contribution, ii) translate the Contribution into other languages, create adaptations, reprints, include within collections and create summaries, extracts and/or, abstracts of the Contribution and convert or allow conversion into any format including without limitation audio, iii)

For peer review only - http://bmjopen.bmj.com/site/about/guidelines.xhtml

create any other derivative work(s) based in whole or part on the on the Contribution, iv) to exploit all subsidiary rights to exploit all subsidiary rights that currently exist or as may exist in the future in the Contribution, v) the inclusion of electronic links from the Contribution to third party material where-ever it may be located; and, vi) licence any third party to do any or all of the above. All research articles will be made available on an open access basis. The terms of such open access shall be governed by a Creative Commons licence—details as to which Creative Commons licence will apply to the research article are set out in our worldwide licence referred to above.

Acknowledgements

We would like to thank Trish Bartley for her input to MBCT therapist training and MBCT fidelity checks. We are grateful to members of our Trial Steering Committee (Chris Leach, Richard Moore and Glenys Parry) and Data Monitoring Committee (Paul Ewings, Andy Field and Joanne MacKenzie) for their valuable advice and support during the project. We acknowledge the additional support provided by the Mental Health and Primary Care Research Networks. We also acknowledge the support provided by the Department of Health and local Primary Care Trusts, in meeting the excess treatment and service support costs associated with the trial. Thanks go to the PREVENT research team, who facilitated wider qualitative work in the trial, including Aaron Causley, Anna Hunt, Pooja Shah, Holly Sugg, Harry Sutton, and Matthew Williams. Above all, we are grateful to the participants for their time in taking part in this trial.

Data Sharing Statement

We will not be making the data publicly available due to its highly confidential and identifiable nature.

Dissemination Declaration

The trial results were disseminated in workshops and via a flyer to all participants who requested this feedback. The findings of this study will be disseminated to relevant audiences through University of Oxford communications.

For peer review only

ABSTRACT

Objectives: This study aimed to describe the recovery journeys of people with a history of recurrent depression who took part in a psychosocial programme designed to teach skills to prevent depressive relapse (mindfulness-based cognitive therapy; MBCT), alongside maintenance antidepressant medication (ADM).

Design: A qualitative study embedded within a multicentre, single blind, randomised controlled trial (the PREVENT trial).

Setting: Primary care urban and rural settings in the United Kingdom.

Participants: 42 people who participated in the MBCT arm of the parent trial were purposively sampled to represent a range of recovery journeys.

Interventions: MBCT involves eight weekly group sessions, with four refresher sessions offered in the year following the end of the programme. It was adapted to offer bespoke support around ADM tapering and discontinuation.

Methods: Written feedback and structured in-depth interviews were collected in the two years after participants undertook MBCT. Data was analysed using thematic analysis and case studies constructed to illustrate the findings.

Results: People with recurrent depression have unique recovery journeys, that shape and are shaped by their pharmacological and psychological treatment choices. Their journeys typically include several over-arching themes: (1) beliefs about the causes of depression, both biological and psychosocial; (2) personal agency, including expectations about their role in recovery and treatment; (3) acceptance, both of depression itself and the recovery journey; (4) quality of life; (5) experiences and perspectives on ADM and ADM tapering-discontinuation, and; (6) the role of General Practioners, both positive and negative.

Conclusions: People with recurrent depression describe unique, complex recovery journeys shaped by their experiences of depression, treatment and interactions with health professionals. Understanding how several themes coalesce for each individual can both support their recovery and treatment choices as well as health professionals in providing more accessible, collaborative, individualised and empowering care.

For peer review only - http://bmjopen.bmj.com/site/about/guidelines.xhtml

(292 words)

Strengths and limitations of the study

Recurrent depression is a leading cause of disability adjusted life years and ADM is the mainstay approach to treatment; this study is the first to describe people's experiences of recovery with an ADM alongside a psychosocial approach designed to support recovery (MBCT).

The sample was relatively large and purposively sampled to illustrate a range of recovery journeys and outcomes.

Participants experiences in the two years following MBCT were sampled, using an innovative approach to supporting participants' to describe the richness of their experiences of recovery and treatment.

The sampling necessarily meant that we did not include people with a history of recurrent depression who were had decided against ADM, tapering and discontinuing their ADM and/or a psychological approaches.

For peer review only

Depression is a major public health problem. Globally more than 264 million people suffer from depression, and lifetime prevalence rates are estimated to be between 6-20%.[1, 2] Furthermore, depression can be a relapsing and recurring condition, and on average people who experience one episode of depression have seven or eight episodes over their lifetime.[3] Clinical guidance typically recommends that people with recurrent depression should take maintenance antidepressants (ADMs) after remission or engage in preventive psychological interventions to maintain recovery.[4] For people with recurrent depression, their recovery journey is shaped by their experiences of depression, “illness model,” interactions with mental health professionals, treatments that have and have not worked, and expectations about what recovery will entail. [5] For many, ADMs are an important part of their recovery. Reasons for long-term ADM use include positive experiences of ADMs, fear of relapse, perceived lack of alternatives, and concerns about withdrawal effects.[6] On the other hand, people also describe a number of reasons for wanting to discontinue ADMs, including feeling better and wanting to test whether depression has gone away, ambivalence and uncertainty about the role of ADMs in recovery, side effects outweighing benefits, questioning whether the self on ADMs is the ‘real self’, and wanting to assert control over their wellbeing.[7]

Research and clinical guidelines suggests that psychological therapies, such as mindfulness-based cognitive therapy (MBCT) and cognitive-behavioural therapy (CBT), can support recovery from depression as well as support discontinuing ADMs.[4, 8-10] A significant proportion of people express a preference for psychological therapies, so they can learn strategies that support recovery without the need for long-term reliance on ADMs [11]. But psychological therapies can be difficult to access, involve significant investment of time and energy and are not effective for everyone [12].

While studies have examined people’s experiences of ADM and MBCT in recovery, none have focused primarily on how they operate alongside one another. This study explores how people with a history of recurrent depression describe their experience of using MBCT and ADM to support their recovery, drawing on both written feedback booklets and more in depth interviews. It was embedded within a randomised controlled trial comparing MBCT with support to taper and discontinue ADMs (henceforth MBCT-TS) and maintenance antidepressants over a two-year period. [13]. These findings

For peer review only - http://bmjopen.bmj.com/site/about/guidelines.xhtml

could inform decision-making between GPs and patients about the journey of management and recovery from recurrent depression.

For peer review only

For peer review only - http://bmjopen.bmj.com/site/about/guidelines.xhtml

METHODS

Study context

This qualitative process evaluation was embedded within the PREVENT trial, a multicentre, single blind, parallel randomised controlled trial, which investigated whether MBCT-TS (n = 212) was superior to maintenance ADMs (n = 212) for the prevention of depressive relapse or recurrence over 24 months (trial design is described in Kuyken et al.).[13-16] The trial found that MBCT-TS was not superior to maintenance ADM in preventing depressive relapse over two years; however, a subsequent individual patient data meta-analysis which included this data suggests MBCT as an alternative to maintenance ADMs.[8] The South West Research Ethics Committee approved the trial [09/H0206/43], which was registered with the International Standard Randomised Controlled Trial Register [ISRCTN26666654] and the Medicines and Healthcare products Regulatory Agency [2009-012428-10]. We present a Statement Concerning reflexivity in the online supplementary materials, which outlines the experience and background of the authors, to acknowledge our theoretical positions and values in relation to the present study (Supplement 1).[17] We also include the COREQ checklist to match our procedures against standard criteria for qualitative research (Supplement 2).

Participants

Participants in the PREVENT trial were recruited from 95 general practices in urban and rural settings in four UK centres, in addition to self-referral.[13] Inclusion criteria were a diagnosis of recurrent major depressive disorder in full or partial remission according to the Diagnostic and Statistical Manual of Mental Disorders-IV;[18] three or more previous major depressive episodes; age 18 years or older; and on a therapeutic dose of maintenance antidepressant drugs in line with the British National Formulary (BNF) and NICE guidance.[4] Exclusion criteria were a current major depressive episode, comorbid diagnoses of current substance misuse; organic brain damage; current or past psychosis, including bipolar disorder; persistent antisocial behaviour; persistent self-injury needing clinical management or therapy; and formal concurrent psychotherapy. All participants gave informed consent before participating in the trial. The full process of recruitment for the PREVENT trial is

For peer review only - http://bmjopen.bmj.com/site/about/guidelines.xhtml

described in Kuyken et al.[16]

This study examined a sub-group of participants from the PREVENT trial (*n* = 42) allocated to the MBCT-TS arm of the trial. Of the 212 participants allocated to receive MBCT-TS, 176 received an adequate dose of treatment (attended four or more group sessions of therapy).[13] The researchers purposively sampled a sub-group of these participants (*n* = 46) to represent a spread of characteristics and experiences with respect to: whether they reported their childhood as having higher or lower levels of abuse, treatment response (relapse/no relapse to a major depressive episode), and ADM discontinuation profile across the 24 month follow-up period (discontinued ADMs, discontinued ADMs but subsequently resumed them, tapered ADMs but never fully discontinued, never tapered or discontinued ADMs).[13] Of the 46 people invited to interview, 42 agreed, which comprised the final sample. Of the four who declined, two had moved away from the area, one was not interested in participating and one participant had changed their contact details and could not be reached. Interviewees did not differ in either baseline characteristics or trial outcomes from the broader study sample (see Table 1).

For peer review only

Table 1. Characteristics of the sample.

	Interviewed (*N* = 42)	All MBCT-TS Participants (N = 212)
Demographic Characteristics		
Female (%)	31 (74)	151 (71)
White (%)	42 (100)	210 (99)
Age (in years)		
M (*SD*)	51.88 (10.51)	50 (12)
Range	25 – 72	22 – 78
Psychiatric Characteristics		
Previous episodes		
< 6 episodes	26 (62)	120 (57)
≥ 6 episodes	16 (38)	92 (43)
Co-morbid mental health diagnoses		
1 or more (%)	15 (36)	75 (35)
Treatment preference at Baseline		
MBCT-TS preference (%)	34 (81)	150 (71)
ADM preference (%)	1 (2)	12 (6)
No preference (%)	7 (17)	50 (24)
Treatment outcome		
Relapse		
n (%) that relapsed during the follow-up phase	23 (55)	94 (44)
Antidepressant usage during the follow-up phase		
Stopped and stayed stopped (%)	13 (31)	67 (32)
Stopped and resumed (%)	9 (21)	57 (27)
Reduced but never stopped (%)	9 (21)	29 (14)
Never stopped or reduced (%)	11 (26)	23 (11)
Residual depression symptoms		
BDI score at baseline, *M* (*SD*)	15.90 (11.35)	13.8 (12.4)
BDI score at 24 month follow-up, *M* (*SD*)	12.39 (12.25)	11.6 (10.9)

Note. BDI = Beck Depression Inventory

For peer review only

MBCT-TS intervention

MBCT-TS comprised MBCT delivered in line with the published treatment manual,[19] but adapted to include a greater focus on developing a relapse/recurrence signature and response plan that explicitly included participants' reduction/discontinuation of ADM (see Kuyken et al.[16] for more detail). The programme involved eight 2¼-hour group sessions, normally over consecutive weeks, with up to four refresher sessions offered in the year following the end of the eight-week programme. Researchers encouraged participants in the MBCT-TS arm to taper and discontinue their maintenance ADMs at several points from the middle of the MBCT-TS course onwards, and provided information to General Practitioners (GPs) and participants about typical tapering/discontinuation regimes and possible withdrawal effects. If participants experienced a relapse/recurrence during the course of the trial, researchers encouraged them to discuss the most appropriate treatment with their GP and made no further requests that they consider tapering/discontinuing their ADMs.

Qualitative data collection

We used both written feedback booklets (collected at two time points, soon after MBCT and then at study end) and interviews (at study end) to gather participants' more in depth experiences of recovery. We combined each participant's interview and written feedback booklet data to form a single account of their experiences. This formed the study's data corpus.

Written feedback booklets

One month after completing MBCT-TS all trial participants were invited to complete a feedback booklet addressing attitudes towards, and experiences of, taking and reducing antidepressant medication; experiences of taking part in MBCT-TS, and MBCT-TS practices; and the impact of MBCT. In addition to the above, participants received a further feedback booklet 24 months later, which asked the same questions as the first booklet but also included questions focussed on participants' experiences in the follow-up period and basic data on the amount and type of mindfulness practice. The booklets are provided in the online supplementary materials (Supplements 3 & 4).

For peer review only - http://bmjopen.bmj.com/site/about/guidelines.xhtml

Interviews

Interviews were semi-structured and normally conducted face-to-face by trained researchers, approximately 24 months after MBCT-TS. They lasted between 45 minutes and one hour and explored experiences during the follow-up period, with questions addressing times of wellness, early signs of potential depressive relapse, and relapses. Questions explored the use and perceived value of mindfulness techniques, use of ADMs, and their combination. We tailored interviews to the specific profile of each participant using a 'timeline' prepared in advance and amended by the participant at the interview, which summarised each participant's ADM use, relapses, and significant life events, as reported to the research team during the trial. The interview schedule was deliberately broad in focus (Supplement 5). Interviews were recorded and transcribed for analysis.

Public and patient involvement

The PREVENT trial benefited from the expertise of many people with lived experience of mental health difficulties including a number of members of a locally organised voluntary group called the Lived Experience Group (LEG). The LEG assisted the PREVENT trial at every stage of its development including both the interview schedule and written feedback booklets. There were reviewed and then trialled by several members of the LEG who suggested a number of fundamental changes. A member of the LEG also provided specific training to the research staff on conducting interviews.

Data Analyses

We used thematic analysis as our analytic approach.[17] First, we selected eight participants with a range of ADM discontinuation journeys during the trial period: two who had discontinued ADMs and remained ADM-free; two who had discontinued ADMs and subsequently resumed; two who had never tapered or discontinued ADMs; and two who had tapered but never discontinued ADMs. Four researchers (AT, CC, JR and WK) independently analysed the interview transcripts and accompanying one-month and 24-month feedback booklets for each participant. In this phase, we conducted inductive analysis, with each researcher developing a preliminary coding frame. These frames were then

For peer review only - http://bmjopen.bmj.com/site/about/guidelines.xhtml

integrated through discussion to remove redundancies and ensure breadth. This collaboratively produced, inductive coding frame was then combined with deductive codes developed from key literature on participant experiences of MBCT,[20, 21] and ADM use,[22, 7] to establish a working coding frame.

The lead researcher (AT) then analysed the 42 interviews and accompanying booklets against this coding frame, using NVivo 11 software. AT, CC, and JR met at regular intervals to discuss additional emerging codes and arising themes and, if deemed appropriate, integrated these into the coding frame. Midway through coding, AT sought peer feedback on emerging themes from co-authors, at an internal research meeting and at a symposium focused on antidepressant tapering at an international conference (Tickell, 2018). Feedback from these presentations helped clarify which themes were particularly important, and in particular helped the researchers reflect on those that related specifically to participants' experiences of ADM alongside MBCT-TS. Once the data were fully coded, the researchers reviewed the themes in the light of the core research question. These were discussed with the wider authorship group, whose input was used to reduce redundancy across themes, and highlight their interactions. Peer review of an earlier version of this manuscript also led to further refining the questions and themes. Finally, we identified cases that illustrated the unique stories of recovery and the ways the common themes coalesced in different ways in illustrative case studies.

For peer review only - http://bmjopen.bmj.com/site/about/guidelines.xhtml

RESULTS

The thematic analysis yielded six over-arching themes, each with a number of constituent sub-themes. We provide a summary (Table 2) and narrative account of each theme and its constituent sub-themes, illustrating these with extracts from participants' accounts. While each person's experience of MBCT and ADMs was unique, these themes converged in complex ways within individual case. Five case examples illustrate these different recovery journeys (Box 1).

Table 2: Themes and Sub-themes

Theme	Sub-themes
**Beliefs about the causes of depression**	*Neurochemical disruption*<br>*Learning a psychological model*<br>*Integrating models*
**Personal agency**	*Control over depression*<br>*Responsibility*
**Acceptance**	*Resolving shame*<br>*Self-care*<br>*Perspectives on relapse*
**Quality of life**	*Experiencing emotions more fully*<br>*From coping to enjoying life*
**ADM tapering/discontinuation**	*Pace of reduction*<br>*Managing withdrawal effects*
**Interactions with GP**	*Presence and support*<br>*Following advice*

For peer review only

For peer review only - http://bmjopen.bmj.com/site/about/guidelines.xhtml

Beliefs about the causes and treatment of depression

This over-arching theme describes participants' beliefs about the causes of depression and how these beliefs influence their treatment decisions. This theme comprises three sub-themes.

Neurochemical disruption

Many participants described entering the study believing that their recurrent depression was due to a neurochemical disruption in their brain, often citing specifically a deficiency or imbalance of the neurotransmitter serotonin. Participants viewed medication as a way to correct this issue and made parallels to biomedical disorders, viewing ADMs as a *"physiological need"* in the same way that *"diabetics require insulin"* because *"there is some chemical missing." (2102; Written feedback, Never tapered or discontinued)*. For instance, Annie explained that she went on ADMs because her doctor told her that she had lower levels of serotonin than other people (see Box 1). This belief appeared to influence expectations about psychological therapy, as some participants stated that they did not understand how "*mindfulness would be able to counteract depression […] if it's generated by a chemical imbalance." (1031, Interview, Never tapered or discontinued)*. Other people said that they had not given much thought to why they were depressed or how ADMs worked: *"Happy pills […] I've never really given it a great deal of thought exactly what they do to be honest. […] I just know I don't feel so bad with them. (2123, Interview, Tapered but never discontinued).*

Learning a psychological model

Participants described how their views on the causes of depression evolved during and following the MBCT-TS programme. Despite some of the initial reservations described above, participants described an open mind as key to engaging with the new psychological model, in which their thoughts, behaviours, and emotions played a role in depressive relapses and recurrences: *"in the first sessions […] I switched from being highly sceptical to very interested very quickly" (1203, Interview, Discontinued).* Some participants articulated a move away from *"treating depression as a disease, like if you had a toothache, so you took pills"*, and were surprised because they "*hadn't thought that there was an alternative" (1069, Interview, Discontinued)*. They began to feel confident to

For peer review only - http://bmjopen.bmj.com/site/about/guidelines.xhtml

discontinue ADMs with the support of psychological therapy. In addition, people described how the programme gave them more awareness of how external factors, such as relationships or financial situations, could trigger or exacerbate depressive relapses and recurrences. On the other hand, some participants found it more difficult to engage in the programme and found themselves *"rebelling against it"* because they did not have *"intellectual confidence in the process." (3105; Written feedback, Never tapered or discontinued).* People described how their initial treatment experiences influenced their attitudes: those who felt that the techniques were helping them to manage depressive relapse/recurrence often endorsed the psychological model. On the other hand, others who experienced deterioration in mood or relapse sometimes reported that they had re-considered bio-medical explanations, and decided to resume or remain on ADMs: *"I really thought depression was a psychosomatic problem, but I am not so sure now. I did give it my best shot, using the mindful techniques, but I still fell into the pit of despair […] I feel that my depression is caused by a chemical imbalance in my body which, at present, is only helped by taking medication." (2200; Written feedback, Tapered but never discontinued).*

Integrating models

Although some participants viewed depression as either biomedical or psychological, many did not see the two models as distinct and found ways to integrate them. For instance, they conceptualised that *"antidepressants hold onto the chemical in your body 'cause you're not making enough of it yourself"*, while MBCT-TS allows you to *"focus your mind onto how to make your own." (1139, Interview, Never tapered or discontinued).* It seemed that participants who viewed these models as compatible were more open to using ADMs and using psychological techniques as an additional way to support their recovery, rather than viewing them as competing treatments. Furthermore, when participants observed the diversity of other people's experiences on the programme, some formed the opinion that there are *"all sorts of depressions"* underpinned by different causes *"just as there are colds and flu's and viruses." (3105, Interview, Never tapered or discontinued).* As such, some reasoned that different people would require different treatment decisions to support recovery: *"My depression is not necessarily the same as other people's […] The*

For peer review only - http://bmjopen.bmj.com/site/about/guidelines.xhtml

right combination of changing lifestyle, specific therapies, medication whatever else it takes – that seems to be different for different people." (3109, Interview, Never tapered or discontinued).

Personal Agency

This over-arching theme describes people's personal agency in their recovery and consequently their treatment choices. People described entering the study fearful about ADM discontinuation, but hopeful that a psychological programme could support them. During MBCT-TS, people spoke about feeling better able to manage their vulnerability to depressive relapse, by using the skills and techniques they learned on the programme. While these enhanced feelings of personal agency were largely viewed as a positive and increased many people's confidence to taper and discontinue ADMs, this was not always the case. For some having more responsibility to manage their condition created a sense of unhelpful pressure. This theme comprises two sub-themes.

Control over depression

People described how their treatment choices affected their sense of control over depression. Some felt that taking ADMs provided a sense of control, as it kept their mood on an even keel. However, this sense of control was contingent on taking ADMs, so many participants recalled how before the trial they would not consider discontinuing because they were afraid that depression would return. Through MBCT, some participants described a change in their sense of personal agency in their recovery, describing a shift from being a *"helpless victim of circumstance,"*, to having more *"control of my feelings and my life" (1123; Written feedback, Tapered but never discontinued)*. They reported increased awareness to recognise the early warning signs of depressive relapse and take steps to respond by applying mindfulness or cognitive-behavioural techniques from a 'toolbox', including things like meditation, activity scheduling, or enlisting social support: *"Before the trial, I didn't have the tools to recognise what was happening. […] I didn't even know I was getting depressed. [Now] if things are difficult I can do something about it." (1203, Interview, Discontinued).* Learning these new skills reduced many people's fears about coming off ADMs, because they felt they had the capacity to prevent or contain depressive relapses. For instance, George said that before the course, he would fall into

For peer review only - http://bmjopen.bmj.com/site/about/guidelines.xhtml

depressive episodes very suddenly and without warning, whereas the skills learned in MBCT-TS gave him more awareness and control to act and prevent relapses before they occurred (see Box 1).

Responsibility

Participants also articulated that learning how they could have more agency over their thoughts, feelings, and behaviours led to an increased sense of responsibility to manage their wellbeing. Most participants viewed this as positive, especially if they were able to use the techniques to manage relapse/recurrence. Some people said they preferred MBCT-TS to taking ADMs because it made recovery feel more like a personal achievement: *"Once I've fallen and I realise that I am depressed, I take myself off and say, do 3 or 4 meditations a day. […] Which to me is better than taking a pill, because I know I've worked to get myself well." (2016, Interview; Written feedback, Discontinued)*.

However, not all participants viewed having more agency and responsibility over their wellbeing positively. In particular, some participants described how this made them feel like it was their fault if they relapsed or felt they had to resume ADMs: *"I feel sad and disappointed that stopping [ADMs] made me feel low again. […] It makes me feel I'm not right in the head compared to others. I also feel annoyed with myself for not utilising MBCT skills learnt better." (2123; Written feedback, Discontinued and resumed).* Furthermore, a substantial number of participants expressed the challenge of finding the time, motivation, or self-discipline to keep up a regular mindfulness practice outside of the group sessions. Therefore, the sense of control did not always feel stable, as it was contingent on finding time to practice and *"do it religiously, otherwise I would be fearful of it not being enough." (2102; Written feedback, Never tapered or discontinued)*. Some were disappointed when they realised that psychological therapy was not an *"all-encompassing cure" (1222, Interview, Discontinued and resumed)* and would involve an active and ongoing process of engagement with the techniques learned.

Acceptance

This over-arching theme describes people's feelings of acceptance towards their history of recurrent depression and ongoing need to manage risk of relapse and recurrence. People reported feeling

For peer review only - http://bmjopen.bmj.com/site/about/guidelines.xhtml

a sense of shame around long-term reliance on ADMs before the trial, feeling it labelled them as an ill person even if feeling well. After the trial, people described an increased sense of acceptance regarding their vulnerability to depression and an increased motivation to engage in self-care to support their recovery. This self-management included either ADMs and/or the psychological techniques for different people. This theme comprises three sub-themes.

Resolving shame

Many participants recalled that before the trial they had felt *"inadequate"* or unable to cope with life compared to other people because of their recurrent depression treating it as a *"guilty secret" (1123; Interview, Tapered but never discontinued).* Taking ADMs had helped some people by reducing the symptoms and allowing them to return to feeling like a *"normal contributing person in society" (2200; Interview, Tapered but never discontinued).* However, others said that having to take ADMs on an ongoing basis gave them an underlying feeling that they were still *"not a well person" (2102; Interviews, Never tapered or discontinued)*, even when the symptoms of depression were absent. For these reasons, some people recalled how before the trial, they found it difficult to name their depression, and *"couldn't even or wouldn't even admit to that"(1031, Interview, Never tapered or discontinued).* Through MBCT-TS, some participants described how they felt able to name their condition as depression for the first time. They discussed how meeting other people in the programme had made them realise that depression was not a negative aspect of their own self-identity, but an aspect of human experience: *"You realise it is part of the human condition rather than you."(1128; Interview, Never tapered or discontinued)*, and it *"confirmed that I am a human, worthwhile person" (2176; Written feedback, Discontinued and resumed)*. This led to increased feelings of acceptance towards depression, because participants experienced a shift away from viewing themselves as abnormal, to seeing depression as a more acceptable response to life's difficulties: *"Giving yourself credit […] 'cause at the end of the day […] our human brain is quite a complex thing, isn't it? […] There's nothing wrong in feeling like it." (2140; Interview, Discontinued and resumed).*

Self-care

For peer review only - http://bmjopen.bmj.com/site/about/guidelines.xhtml

Participants described how developing more acceptance towards their condition improved their attitudes towards self-care. They said that accepting their vulnerability to depression allowed them to *"look at solutions"* and that they finally had "*consent to actually do something about it" (1031, Interview, Never tapered or discontinued)*. People described how they increasingly accepted that they needed to take care of themselves, and explained how the programme had taught them legitimate ways to do this, such as using mindfulness practices: *"Previously was a mindset […] that I wasn't allowed to help myself feel better. […] Whereas this felt a way that I could do it without mollycoddling myself." (1031, Interview, Never tapered or discontinued).* Participants also described how the programme had reframed self-care not as something *"fluffy",* but as *"practical"* and a necessary part of their ongoing recovery: *"It doesn't make you any less male of course. [Chuckles] Or any less powerful." (1203, Interview, Discontinued).* In some cases, this new attitude towards self-care caused a shift such that people felt more acceptance towards taking ADMs: *"I don't feel any more when I take my pill every morning that there's something wrong with me"* as they recognised it was important to do *"everything in my power to help myself." (1177, Interview, Tapered but never discontinued).* Some participants also described how originally, they had taken ADMs unwillingly, whereas after MBCT-TS they decided to take ADMs as an act of effective self-management: *"I used to hate taking them [ADMs] I accept [now] it's all about looking after yourself isn't it?" (3103; Interview, Discontinued and resumed).*

Perspectives on relapse

One dimension of acceptance was people's perspectives on mood fluctuations and relapse itself. Some people favoured ADMs as an approach to relapse prevention, because it guaranteed them stability in their mood. For instance, when Greta experienced a deterioration in mood, she interpreted this as a sign that the MBCT-TS programme had been a *"failure"* and she resumed taking ADMs (see Box 1). However, this was not always the case, and many people described how participating in MBCT-TS changed their attitude towards relapse/recurrence. In particular, some people felt more able to accept mood fluctuations and even periods of depression. They described approaching them in a different way, "*thinking it was a phase that one was going through and sort of accepting, okay this is how you're feeling today" (1159; Interview, Discontinued and resumed).* Some people reported that they no longer

For peer review only - http://bmjopen.bmj.com/site/about/guidelines.xhtml

wanted to *"blank out their negative emotions"*, and so did not resume ADMs, even if they relapsed: *"it's definitely helped me to realise that they [negative emotions] are a part of me as well." (4057, Interview, Discontinued).*

Quality of Life

People reflected on the ways in which treatment choices influenced their quality of life; specifically, moving them from a place of coping, to a position where they could enjoy and appreciate their lives. This theme comprises two sub-themes.

Experiencing emotions more fully

Upon reflecting on their experiences with ADMs, some participants said that while ADMs lessened their low mood, at the same time they *"dampen all other emotions"*, for instance, they could not feel *"blissfully happy, couldn't get angry, and in hindsight feel I was sedated." (4057, Written feedback, Discontinued).* In the context of depression, some people viewed this numbing effect as helpful, and reflected that while ADMs "*take away the euphoria that you would get when you've done something really, really, really good"*, this was *"a small price to pay really for not having the really dark times." (2200; Interviews, Tapered but never discontinued).* However, many people thought that this had negatively affected their quality of life, especially in cases where they found it hard to experience positive emotions. This appeared to influence people's decision to taper or discontinue ADMs, because they said that restoring their emotional range was an important part of their long-term vision of recovery: both George and Claire described this as a key motivator to discontinue their ADMs (see Box 1). Indeed, people described how their emotional capacity increased after coming off ADMs: *"I am more alive: my emotions aren't "levelled out" anymore. I can be happy, sad, angry or calm instead of just bland." (4057, Written feedback, Discontinued).* Despite this, some people found it a bit of a *"shock"* at first, when faced with *"very extreme emotions and feelings"* again *(1212; Feedback booklets, Discontinued).* Therefore, people found it helpful that the programme taught them techniques to help manage this transition: *"I definitely used mindfulness during coming off the tablets to […] be aware what's going on inside and […] calm myself down, to have those little islands of tranquillity."*

For peer review only - http://bmjopen.bmj.com/site/about/guidelines.xhtml

(4057, Written feedback, Discontinued). On the other hand, some participants said that despite not tapering or discontinuing ADMs, the programme had helped them to cultivate more positive emotions, and appeared to increase their quality of life on ADMs: *"I suppose the mindfulness in that respect has helped because […] by slowing yourself down you can […] capture some of that […] joy of life that possibly I would have lost." (2200; Interviews, Tapered but never discontinued).*

From coping to enjoying life

Many people reflected that in their recovery journey they had been grateful for the periods of time where they were simply able to function. However, some participants said that the programme had helped them to move beyond that mind-set, and to develop more wellbeing and appreciate life: *"What has changed? I think my outlook on life, I love life, I really do […] People said to me […] before you used to skulk into the room, now you light up the room. […] I do enjoy life now, where I didn't before." (2016, Interview, Discontinued).* They valued the fact that the programme had an active focus on positive functioning, and encouraged them to take part in activities that brought happiness and joy into their life. Participants described this as an active process, facilitated by a sense of having more control and autonomy over making positive decisions in their life: *"I rearranged my life so that the things I do now are things that I enjoy and want to do." (1203, Interviews, Discontinued); "I am now making bigger future plans to make my life better and introducing new ventures." (1031, Written feedback, Never tapered or discontinued).*

ADM tapering/discontinuation

The study's focus included people's experiences of tapering and discontinuing ADMs in the context of the MBCT-TS programme. This theme describes participants' views of what helped or hindered the process of discontinuation. It comprises two sub-themes.

Timing

Reflecting on the right time to engage with different treatments, many participants felt that ADMs were helpful when they first became depressed: *"they got me out of my initial depression so that*

For peer review only - http://bmjopen.bmj.com/site/about/guidelines.xhtml

I could cope more with just everyday life" (4007, Interview, Never tapered or discontinued). However, many did not envisage being on ADMs indefinitely, and they described an increasing need for insight and self-management of depression as time went on. They thought that the MBCT-TS techniques required more effort, but supported a longer-term vision of recovery, to *"recognise what makes you depressed and to give you a way to cope with your depression throughout your life for the long-term, and a way that you can come off [ADMs]." (4007, Interview, Never reduced or discontinued).* As illustrated by this quotation, some participants viewed the MBCT-TS skills as part of a longer-term solution to ADM discontinuation, which extended beyond the two-year follow-up period. Some participants reflected on how they thought the two treatments could be used in combination to support people at different parts of their journey, from depression through to recovery: "*I think you need that initial boost of antidepressant to perhaps get you back into a more rational level, and then once you've reached that, then bring in the MBCT, until you get back then you know, be weaned off. I can see that working very well really." (1108, Interview, Never reduced or discontinued).*

Participants discussed the pace at which they tapered ADMs, and how they perceived this to have influenced their outcomes. Some people who were worried about coming off ADMs shared that they exercised caution, testing out the psychological techniques for a set period and tapering slowly. They said this was helpful as it gave them time to learn to use the psychological techniques before giving up the support of their ADMs, *"by doing it slowly, you are learning those skills and you are finding out how you can use it. [Then] you can start dropping it [ADMs] at your own pace." (1075; Interviews, Discontinued).* In comparison, those who were keen to come off ADMs and were less fearful of the consequences described tapering more quickly. Although the programme had included explicit guidance to taper gradually, participants' reports suggested that many people had gone against this advice, and were looking for a *"quick fix"* to *"get off the pills as quick as possible" (2131, Interview, Tapered but never discontinued).* However, upon reflection many people thought, *"perhaps that wasn't the answer perhaps the thing ought to be graded on over a longer period." (2131, Interviews, Tapered but never discontinued)*. Some of these participants reflected that in retrospect they should have been more cautious, and tapering too quickly had led to poorer outcomes: *"I reduced my tablets too quick*

For peer review only - http://bmjopen.bmj.com/site/about/guidelines.xhtml

and paid the price by having to get straight back to the full dose" (2016, Interview; Written feedback, Discontinued). However, some people, like Claire, who did not successfully discontinue on their first attempt reported how they had then tried again, tapering more gradually and with more success (see Box 1).

Managing withdrawal effects

People said that the programme had helped them to cope with withdrawal effects during and after tapering/discontinuing ADMs. They described how the group and the meditation techniques provided ongoing support to manage this period: *"I used meditation techniques […] tried to treat myself with pleasurable experiences and told myself that this would pass over. […] I had a network of fellow participants and a trustworthy instructor. All of this put me in a position of confidence that it would work this time." (4057, Written feedback, Discontinued)*. In addition, people said that they were better able to differentiate the side effects of ADM withdrawal from a depressive relapse. For instance, Mandy said that in the past, withdrawal effects had been the biggest hindrance to tapering ADMs, because she had always mistaken them for a depressive relapse and resumed her medication. On the programme, she learned how to differentiate between these effects and *"real relapses"*, and said that tapering was relatively *"easy"* this time around (see Box 1). Indeed, some people recalled attempting to discontinue ADMs before the trial and their withdrawal symptoms being "*misdiagnosed" "as recurring depression,"* whereas this time they *"knew what was coming" (4057, Written feedback, Discontinued).*

Interactions with GP

Participants' described their interactions with their GPs as being important in their recovery, in both positive and negative ways. This theme comprises two sub-themes.

Presence and support

Participants described having a GP who was easy to access throughout the process of discontinuation as supportive: *"Knowing that I could ring the doctor and say, "I need to make an appointment, I need to come and see you." There was always that net underneath me to catch me if I*

For peer review only - http://bmjopen.bmj.com/site/about/guidelines.xhtml

For peer review only

was falling and I couldn't stop it." (2090, Interview, Discontinued), whereas some participants said they found it *"very difficult"* to access their GPs, and so felt *"unsupported" (1123, Written feedback, Tapered but never discontinued).* Participants reported a more positive attitude to the programme if their GP had endorsed it, and some said they had only been convinced to take part in the trial because their GPs said they had themselves done a mindfulness course. When GPs encouraged their patients to use the mindfulness practices, this appeared to be associated with better engagement and subsequent success in ADM tapering and discontinuation: *"I did reach a stage where I went to see my G.P. as the depression was returning. […] We decided that I should try the exercises before trying pills. I did not need to go back on them yet […] My GP is a great help." (2090, Written feedback, Discontinued).*

Following advice

Participants differed in the extent to which they sought and followed the advice of their GPs. For instance, some participants described that they remained on their ADMs at their GP's suggestion: *"My GP would not allow me to come off my antidepressant or reduce it because I had been on them so long term. [I am] relieved but also a bit disappointed." (1108; Written feedback, Never tapered or discontinued).* This adherence to medical advice seemed to be greater for participants who had more concerns about discontinuation. For instance, Claire, who relapsed the first time that she had attempted tapering and discontinuation, was much more receptive to her GP's advice the second time around, because she was afraid of relapsing again (see Box 1). On the other hand, where people were confident that they had learned the skills to self-manage their depression without ADMs, they more often reported that they could manage the process independently, and placed less value on their GP's advice: *"I went along to the doctors because I was polite to ask him if I could stop taking it. And he said, "Well yeah maybe in a few months time you can taper it- ease it off a bit." But really I had decided (laughs) I was going to stop. So I was just there out of politeness." (1203; Interview, Discontinued).*

For peer review only - http://bmjopen.bmj.com/site/about/guidelines.xhtml

DISCUSSION

Statement of principal findings

The current study explored the recovery journeys of people with recurrent depression who followed a programme (MBCT-TS), designed to teach psychological skills to prevent depressive relapse whilst providing advice to encourage tapering and discontinuation of maintenance ADMs.[13] Thematic analysis suggested people have unique recovery journeys, but tend to be characterized by six common themes. Five illustrative stories are represented in case studies (Box 1). The over-arching themes in participants' accounts were: beliefs about the causes of depression, personal agency, acceptance, quality of life, ADM tapering/discontinuation and, interactions with GP (Table 2). Together, these findings have the potential to facilitate discussions between clinicians and patients about the depression recovery journey. The findings also provide a starting point for more research into which treatments for recurrent depression, or combination of treatments, work best for whom and when.

Strengths and weaknesses of the study

This study had a number of methodological strengths. We had a relatively large sample and purposively sampled the population for whom this research is relevant – people with a history of recurrent depression, stable on maintenance ADMs who were open to both a psychological and pharmacological approach to recovery. The study's time frame enabled participants to reflect on their journey with MBCT-TS and ADMs over two years. To support participants recollection we developed prompts about the course of their depression and ADM use over the two-year period based on information we had collected as part of the parent RCT.

Alongside these strengths, it is important to consider the context within which the study took place and its implications for interpretation of the findings. First, the trial was pragmatic in that it recruited participants from a particular population.[16] However, it did not include people either unwilling to consider a psychological therapy or unwilling to consider tapering/discontinuing their

For peer review only - http://bmjopen.bmj.com/site/about/guidelines.xhtml

medication. Second, the parent trial included monitoring participants' use of ADM, and if people following MBCT-TS were not tapering/discontinuing they were invited to discuss this with their GPs. Some participants reported feeling pressured to discontinue ADMs and it is reasonable to assume that some participants may have made different decisions in a more naturalistic setting. Third, our purposive sampling means this study does not speak to a larger population of people with a history of depression who are not interested in a psychological approach and tapering/discontinuing their ADMs, or indeed prefer not to use ADM. Fourth, the questions in our feedback booklets and interviews had a particular framing, and it is possible that if the questions were framed differently the answers too may have been different. Finally, for pragmatic reasons we did not ask participants' feedback on the themes as is sometimes done in qualitative research.

Implications of our findings

Several randomised controlled trials have demonstrated that psychological therapies such as CBT and MBCT can support ADM discontinuation,[8-10] but to our knowledge, no qualitative studies have examined people's experiences of this process. This study adds to the body of literature suggesting that people's journey involves choices among different treatments, shaped by their prior beliefs, expectations, experiences and interactions with their GPs. In both MBCT and CBT people learn new skills to manage depressive symptoms, gaining new perspectives drawn from both the psychological model and peer-to-peer learning, and develop an increased sense of agency concerning ADM discontinuation. [11, 20, 21] In this study participants described learning attitudes towards self-care that were participatory and empowering, which facilitated a sense of agency around ADM use, tapering and discontinuation. On the other hand, some people's biological beliefs about depression, positive experiences of ADM, and/or negative experiences of psychological therapies meant they were happy to use ADM as their primary approach to recovery.

People also emphasised the importance of a GP who is accessible and able to provide support that is collaborative, individualised and empowering, with careful monitoring over time. Moreover, they described how GPs had powerfully shared their models of depression, expectations of treatment

For peer review only - http://bmjopen.bmj.com/site/about/guidelines.xhtml

and treatment choices. The implication for GPs is to provide accessible, collaborative, individualised and empowering care. Moreover GPs should provide people with explanatory models of depression that are bio-psycho-social alongside appropriate pharmacological and psychological treatment choices. Our findings alongside others [21-23] also suggest GPs should not offer an overly simplistic biological model, e.g., "your serotonin levels are low," followed by a (repeat) prescription of ADM.

ADMs are currently the mainstay treatment approach to recurrent depression. Kendrick has argued that many people remain on ADMs without clinical need and could benefit from support and guidance on how to discontinue, especially regarding how to deal with initial withdrawal symptoms.[23] Our findings underscore this. Moreover, participants spoke of the importance of feeling that they had acquired alternative skills in MBCT-TS to support their recovery generally by being able to manage their depression, but also ADM tapering and discontinuation specifically. For example, where life circumstances were challenging some people felt that the time was not right for them to discontinue ADMs; they made an informed decision to continue with their medication. Even so, the majority of participants who remained on ADMs reported that the MBCT-TS treatment had increased their quality of life on ADMs, and improved their confidence in future discontinuation when circumstances were more favourable. Our analysis also outlines participants' views on the appropriate timing of different treatments, which provide ideas for when it might be an appropriate time to initiate conversations about ADM and MBCT treatment choices.

People described how their expectations of both MBCT and ADM influenced their treatment choices. Although it is widely assumed that positive expectations predict greater benefit in psychological therapy, in our sample both unrealistically positive expectations (e.g. expecting MBCT-TS to be an *"all-encompassing cure-all"*) and very negative expectations (having *"no intellectual confidence in the process"*) appeared to act as a barrier to engagement. These findings are consistent with those of Malpass et al.[7] and suggest that openly discussing expectations at key junctures is likely to be key in preventing disappointment or disengagement from what is an effortful process of change. Likewise, in line with Maund et al.'s findings,[6] people's causal models of depression also appeared to influence their expectations and engagement with psychological therapy. Moreover, they were

For peer review only - http://bmjopen.bmj.com/site/about/guidelines.xhtml

subject to change during and beyond the therapy process, as their experiences either confirmed or disconfirmed their expectations and working model of depression and its treatment. People who suffer from depression frequently endorse biomedical explanations,[24] and this was evident in our sample. A number of people reported that they derived these models from discussions with their GPs as a rationale for taking ADMs. Previous research suggests that conceiving depression as a biomedical illness can absolve people of personal responsibility and thus challenge stereotypes of depression resulting from personal weakness.[7] However, our findings suggest that strongly held biomedical beliefs appeared to increase feelings of dependency on ADMs, and contribute to negative expectations and lack of engagement with psychological therapy. On the other hand, while learning a psychological model of depression empowered people towards more self-management of depression and feelings of mastery over their emotional wellbeing, in some cases, when people developed a psychological understanding and then went on to relapse, they blamed themselves. In some cases, practical life circumstances also made it very difficult for people to engage in an approach that required time and effort. Together, this suggests that polarised beliefs about the causes of depression can either compromise self-efficacy or promote self-blame. Many participants found it helpful to bridge biomedical and psychological theories, with parallels to a 'biopsychosocial' framework,[25] rather than viewing separate theories as competing, which seemed to foster more flexibility, self-compassion and open-mindedness towards trying different treatment options at different times in their journey of managing recurrent depression. This highlights the importance of recognising that a myriad of factors, including genetic vulnerability and challenging social circumstances can influence depression.

People sometimes describe ADMs as sedating or numbing [26]. In these interviews participants said this could influence their decision making about ADM use. For example, in the instance of numbing, some people viewed this as helpful as it reduced their feelings of depression, whereas other people said that ADMs numbed all of their emotions, including positive feelings, and this contributed towards a desire to discontinue them. These findings add to ongoing discussion about the psychoactive effects of ADMs, including their potential benefits and costs, how these effects impact people's

For peer review only - http://bmjopen.bmj.com/site/about/guidelines.xhtml

experience of recovery from depression, and how participating in psychological therapy can interact with these experiences.

Finally, descriptions of the role of the GP in supporting ADM discontinuation varied markedly, and this appeared to result both from differences between patients in their preferred level of guidance and support, and the availability of their GPs to provide this. For example, some people adhered to their GP's advice although this was in conflict with their own desired approach, some described informing their GP of their intentions as an act of courtesy, and some did not involve their GP at all. In some of these latter cases, participants felt that they would have benefited from more support, but their GP, for a range of reasons, was unable to provide this. People also described needing more understanding and support over time as they took more responsibility for managing their depression. This is in line with findings from Malpass et al.,[7] who suggested that people vary in the extent to which they want to be involved in treatment decision-making, and their preferences for involvement are dynamic, not static. Archer has described different 'modes of reflexivity' noting the varying degrees to which people act autonomously or rely on endorsement from others.[27] Moreover, recovery meant different things to different people, and overall, the outcome most important to patients appeared to be their day-to-day functioning and quality of life. It is likely that when GPs able to recognise their patients' preferred mode of engagement, and complex, dynamic views of recovery and adapt their approach accordingly, this will facilitate patient-GP consultations about ADM and psychological therapies treatment choices.

Unanswered questions and future research

This work adds to the emerging literature on people's experiences of recovery from depression with ADM and psychological therapies. Applied research asking how patients and health professionals communicate about their respective models of depression, and understand the how this affects treatment decisions, compliance, outcomes and a broader conceptualization of recovery would be valuable. Extending this to the broader population of people who suffer depression would not only provide an interesting and important alternate perspective, buy also be important to consider with respect to recovery journeys and treatment choices.

For peer review only - http://bmjopen.bmj.com/site/about/guidelines.xhtml

Our work took a qualitative approach. An obvious next question asks how these process variables affect outcomes. That is to say, what works for who, how, when, to affect treatment outcomes? Finally, such research should prioritise the outcome that is most meaningful to patients: their day-to-day functioning and quality of life.

For peer review only

For peer review only - http://bmjopen.bmj.com/site/about/guidelines.xhtml

REFERENCES

1. Kessler R C & Bromet E J. The Epidemiology of Depression Across Cultures. *Annu Rev Public Health* 2013;34:119–38. https://doi.org/10.1146/annurev-publhealth-031912-114409
2. GBD 2017 Disease and Injury Incidence and Prevalence Collaborators. Global, regional, and national incidence, prevalence, and years lived with disability for 354 diseases and injuries for 195 countries and territories, 1990–2017: a systematic analysis for the Global Burden of Disease Study 2017. *Lancet* 2018;392(10159):1789-1858. https://doi.org/10.1016/S0140-6736(18)32279-7
3. Kruijshaar M E, Barendregt J, Vos T, et al. Lifetime prevalence estimates of major depression: An indirect estimation method and a quantification of recall bias. *Eur J Epidemiol* 2005;20(1):103–111. https://doi.org/10.1007/s10654-004-1009-0
4. National Institute of Health and Care Excellence (NICE). *Depression: The treatment and management of depression in adults (update)*. London: Author 2009. https://www.nice.org.uk/guidance/cg90 (accessed 18 May 2019).
5. Bosman R, Huijbregts K, Verhaak P, et al. Long-term antidepressant use: a qualitative study on perspectives of patients and GPs in primary care. *Br J Gen Pract* 2016;66(651):e708-e719. https://doi.org/10.3399/bjgp16X686641
6. Maund E, Dewar-Haggarta R, Williams S, et al. Barriers and facilitators to discontinuing antidepressant use: A systematic review and thematic synthesis. *J Affect Disord* 2019;245:38-62. https://doi.org/10.1016/j.jad.2018.10.107
7. Malpass A, Shawa A, Sharp D, et al. ''Medication career'' or ''Moral career''? The two sides of managing antidepressants: A meta-ethnography of patients' experience of antidepressants. *Soc Sci Med* 2009;68:154-168. https://doi.org/10.1016/j.socscimed.2008.09.068
8. Kuyken W, Warren F C, Taylor R S, et al. Efficacy of mindfulness-based cognitive therapy in prevention of depressive relapse: An individual patient data meta-analysis from randomized trials. *JAMA Psychiatry* 2016;73(6):565–574. https://doi.org/10.1001/jamapsychiatry.2016.0076
9. Maund E, Stuart B, Moore M, et al. Managing antidepressant discontinuation: a systematic review. *Ann Fam Med* 2019;17:52-60. https://doi.org/10.1370/afm.2336
10. Bockting C, Klein N, Elgersma H, et al. Effectiveness of preventive cognitive therapy while tapering antidepressants versus maintenance antidepressant treatment versus their combination in prevention of depressive relapse or recurrence (DRD study): a three-group, multicentre, randomised controlled trial. *Lancet Psychiatry* 2018;5(5):401-410. https://doi.org/10.1016/S2215-0366(18)30100-7
11. Cuijpers P, Quero S, Dowrick C, et al. Psychological Treatment of Depression in Primary Care: Recent Developments. *Cur Psychiatry Rep* 2019; 21:129. https://doi.org/10.1007/s11920-019-1117-x
12. Bockting C, Hollon S, Jarrett R, et al. A lifetime approach to major depressive disorder: The contributions of psychological interventions in preventing relapse and recurrence. *Clin Psychol Rev* 2015;41:16-26. https://doi.org/10.1016/j.cpr.2015.02.003
13. Kuyken W, Hayes R, Barrett B, et al. Effectiveness and cost-effectiveness of mindfulness-based cognitive therapy compared with maintenance antidepressant treatment in the prevention of depressive relapse or recurrence (PREVENT): A randomised controlled trial. *Lancet* 2015;386(9988):63–73. https://doi.org/10.1016/S0140-6736(14)62222-4
14. Kuyken W, Byford S, Byng R, et al. Study protocol for a randomised controlled trial comparing mindfulness-based cognitive therapy with maintenance anti-depressant treatment in the prevention of depressive relapse/recurrence: the PREVENT trial. *Trials* 2010;11(99):1-10. https://doi.org/10.1186/1745-6215-11-99
15. Kuyken W, Byford S, Byng R, et al. Update to the study protocol for a randomized controlled trial comparing mindfulness-based cognitive therapy with maintenance anti-depressant

For peer review only - http://bmjopen.bmj.com/site/about/guidelines.xhtml

treatment depressive relapse/recurrence: the PREVENT trial. *Trials* 2014;15(1). https://doi.org/10.1186/1745-6215-15-217

16. Kuyken W, Hayes R, Barrett B, et al. The effectiveness and cost-effectiveness of mindfulness-based cognitive therapy compared with maintenance antidepressant treatment in the prevention of depressive relapse/recurrence: results of a randomised controlled trial (the PREVENT study). *Health Technol Assess* 2015;19(73):1-124. https://doi.org/10.3310/hta19730
17. Braun V & Clarke V. Using thematic analysis in psychology. *Qual Res Psychol* 2006;3(2):77-101. http://dx.doi.org/10.1191/1478088706qp063oa
18. American Psychiatric Association. *Diagnostic and statistical manual of mental disorders* (4th ed., text rev.). Washington, DC: Author 2000.
19. Segal Z V, Williams J M G, & Teasdale J D. *Mindfulness- based cognitive therapy for depression: A new approach to preventing relapse*. New York: Guilford Press 2002.
20. Allen M, Bromley A, Kuyken W, et al. Participants' experiences of mindfulness-based cognitive therapy: "It changed me in just about every way possible". *Behav Cogn Psychother* 2009;37:413-430. https://doi.org/10.1017/S135246580999004X
21. Malpass A, Carel H, Ridd M, et al. Transforming the perceptual situation: a meta-ethnography of qualitative work reporting patients' experiences of mindfulness-based approaches. *Mindfulness* 2012;3:60-75. https://doi.org/10.1007/s12671-011-0081-2
22. Ridge D, Kokanovic R, Broom A, et al. My dirty little secret: Patient constructions of anti-depressant use and the crisis of legitimacy. *Soc Sci Med* 2015;146:53-61. http://dx.doi.org/10.1016/j.socscimed.2015.10.012 0277-9536
23. Kendrick, A. Reviewing long-term anti-depressant use by careful monitoring in everyday practice (REDUCE) programme. 2016 https://europepmc.org/grantfinder/grantdetails?query=pi:%22Kendrick+A%22+gid:%22RP-PG-1214-20004%22+ga:%22DH/NIHR%22 (accessed 18 May 2019).
24. Kuyken W, Brewin C R, Power M J, et al. Causal beliefs about depression in depressed patients, clinical psychologists and lay persons. *Br J Med Psychol* 1992;65:257-268. https://doi.org/10.1111/j.2044-8341.1992.tb01706.x
25. Engel G. The need for a new medical model: A challenge for biomedical science. *Science* 1977;196(4286):126-9. Retrieved from: https://www.jstor.org/stable/1743658
26. Moncrieff J, Cohen D, & Porter S. The Psychoactive Effects of Psychiatric Medication: The Elephant in the Room. *J Psychoactive Drugs* 2013;45(5):409-415. http://doi.org/10.1080/02791072.2013.845328
27. Archer M. S. Routine, reflexivity, and realism. *Sociol Theory* 2010;28(3):272-303. https://doi.org/10.1111/j.1467-9558.2010.01375.x

For peer review only

BOX 1: CASE EXAMPLES

Mandy. *Mandy, aged 57, experienced nine episodes of depression, beginning when she was 32. Following the MBCT-TS course Mandy successfully discontinued her ADM treatment. She did not experience a relapse over the 24-month follow-up period.*

Mandy recalled how ADMs had helped her to function well. In the past, she had tried tapering, but had always relapsed, so assumed that ADMs would be a part of her life forever. At first, Mandy was nervous, but was willing to try tapering ADMs gradually and with the support of MBCT-TS **(Personal Agency: Control Over Depression)**. Mandy's GP was supportive, but reassured her that it was ultimately her decision **(Interactions with GP: Presence and Support)**. During the MBCT-TS course, Mandy said that she learned a different model of depression and developed a better understanding of *"how the mind works"* **(Beliefs about the Causes of Depression: Learning a Psychological Model)**. She felt more confident about tapering, and said that this time it was *"so easy, knowing that I have been given tools to help me through it if needed"*, and found the course *"totally liberating"* as it gave her the chance to take control of her depression, rather than the other way round **(Personal Agency: Responsibility).** She also found it helpful to learn about the possible symptoms of withdrawal, which included mood swings. Mandy realised that the relapses she had experienced when she had tried to taper her ADMs in the past might have been withdrawal symptoms, as opposed to *"real relapses"* **(ADM Tapering / Discontinuation: Managing Withdrawal Effects)**. At the time of interview, having discontinued ADMs, Mandy still practised what she learned in MBCT-TS and made it part of her daily routine. She accepted that if she ever relapsed, she could use ADMs, but it would only ever be a short-term solution, because she has the MBCT-TS skills as a *"weapon"* to help her manage **(Acceptance: Self-Care)**.

Greta. *Greta, aged 72, had experienced three episodes of depression, beginning when she was 33. Following the MBCT-TS course she discontinued her ADMs but then resumed following a deterioration in mood.*

For peer review only - http://bmjopen.bmj.com/site/about/guidelines.xhtml

Greta was very optimistic about the course because she hated being on ADMs, which gave her unpleasant side effects that interfered with her quality of life **(Personal Agency: Control over Depression)**. At first, Greta said the course made an *"immense difference"* to her, and she described learning how to combat the negative thoughts and feelings she was having **(Beliefs about the Causes of Depression: Learning a Psychological Model)**. The programme left Greta feeling *"so well and positive"* that she decided to taper her ADMs very quickly **(ADM Tapering/Discontinuation: Pace of Reduction)**, but began to feel her mood dipping. Greta thought this must be a sign that the programme was not working, because she should not feel depressed **(Acceptance: Perspectives on Relapse)**. Greta went to her GP, who did not seem interested in the programme and told her to resume ADMs immediately **(Interactions with GP: Presence and Support).** She was disappointed and felt *"guilty"* that she was not able to use these new skills to keep herself well **(Personal Agency: Responsibility).** She stopped practising mindfulness, although the programme made her remember to appreciate the high points in her day and experience more joy **(Quality of Life: From Coping to Enjoying Life).**

Annie. *Annie, aged 48, had experienced five episodes of depression, beginning when she was 23. Following the MBCT-TS programme, she discontinued her ADMs but then resumed them later.*

Annie felt that ADMs had a positive impact on her life, allowing her to cope day-to-day as a full-time carer for her husband who had a disability. At first, she was very reluctant to try discontinuing ADMs because she believed she might have low levels of serotonin **(Beliefs about the Causes of Depression: Neurochemical Disruption)**. However, the programme taught her a new model of understanding depression **(Beliefs about the Causes of Depression: Learning a Psychological Model)**, which made her feel empowered to practice the psychological techniques **(Personal Agency: Responsibility)**. She started to taper off ADMs, but then her mother died and her husband's health deteriorated, so it was difficult to find time to practice mindfulness. Her GP advised her it was probably not a good time to discontinue **(Interactions with GP: Presence and Support)**, so she resumed ADMs**.** However, Annie still incorporated the mindfulness exercises into her everyday life, which brought her more joy **(Quality of Life: Experiencing Emotions More Fully)**. She also recognised that it is not her fault when she felt depressed, given how challenging her life was **(Acceptance: Resolving Shame).** Annie felt that the

For peer review only - http://bmjopen.bmj.com/site/about/guidelines.xhtml

best way to manage her depression was to combine ADMs with mindfulness practices, which gave her more skills to look after herself during difficult times **(Acceptance: Self-Care).** She felt hopeful that one day she would discontinue ADMs, when her life circumstances were more stable.

George. *George, aged 37, had experienced ten episodes of depression, beginning when he was 16. Following the MBCT-TS programme he discontinued his ADMs. He experienced a relapse to depression during the 24-month follow-up.*

George was very optimistic about trying an alternative to ADMs, because they made him feel like a *"zombie"*. Having experienced substance misuse issues in the past George had the goal of being totally *"chemical free"*. Before the course, George felt he had no control over his depression symptoms, and his mood would deteriorate suddenly without warning **(Personal Agency: Control)**. Through practising the mindfulness skills, he described developing more awareness of his emotions and felt he would have the skills to manage them **(Personal Agency: Responsibility).** George said that the best part of taking part in MBCT-TS was meeting other people with depression, which made him feel more accepting of himself **(Acceptance: Resolving Shame)**. He felt that ADMs had masked his symptoms, whereas MBCT-TS allowed him to explore the problems in his life that were contributing to depression and work through them to make long-term changes **(Personal Agency: Responsibility).** When George relapsed shortly after discontinuing ADMs, he carried on practising MBCT-TS and said that the skills he learned were enough to pull him out of that period of low mood **(Acceptance: Perspectives on Relapse).**

Claire. *Claire, aged 49, had experienced four episodes of depression, beginning when she was 17. Following the MBCT-TS programme, Claire discontinued her ADMs. She relapsed and resumed medication, but subsequently tapered and discontinued again, and was not using ADMs at the time of her follow-up interview.*

At first, Claire was very sceptical about the MBCT-TS programme and thought it might all be *"mumbo jumbo"*. However, she was very keen to come off ADMs, so she approached the programme with an open mind and wanted to give it her all **(Personal Agency: Control)**. As the course progressed, Claire

For peer review only - http://bmjopen.bmj.com/site/about/guidelines.xhtml

changed her mind and began to *"believe more and more that this might help me"*. MBCT-TS gave her new ways to cope with her feelings, which shocked her because she *"had never took control of my depression before"* **(Personal Agency: Responsibility).** She became very excited and tapered off her medication *"too quickly"* and *"hit a brick wall in a short amount of time. Went straight back in to a deep depression"* (**ADM Tapering/Discontinuation: Pace of Reduction**). Her doctor was very understanding, and did not push her to do anything, but advised her to go back on ADMs and try to taper off again when she was feeling better **(Interactions with GP: Presence and Support).** He said that she should try tapering them more slowly next time even though she *"wanted to get off them as soon as possible"*. This time, she did *"exactly as she was told"* and did not experience a relapse **(Interactions with GP: Following Advice)**. Claire was very pleased because she said they had always felt that ADMs had *"suppressed"* her and that the person she was when taking ADMs *"wasn't really me"* **(Quality of Life: Experiencing Emotions More Fully)**.

For peer review only

Statement Concerning Reflexivity

Interviews were conducted by members of the PREVENT trial research team (see Kuyken et al., 2015), following training and using a standard protocol and semi-structured interview schedule. Both male and female interviewers gathered data. Interviewers had knowledge of the participants' treatment journeys prior to conducting their 24-month interviews (from reviewing their files and in some cases as a result of their involvement in earlier waves of data collection). The protocol included interviewers familiarizing themselves with any information about treatment experiences and trajectories of participants, which were held in study records, as part of the interview preparation process. Researchers did not know participants prior to their entry to the trial, and had no association with them outside the context of the trial and associated research assessments.

All/Some interviewers had undertaken mindfulness training, acting as participant observers in MBCT courses, or in other contexts. This personal knowledge enabled them to understand the nuances in participants' descriptions of their experiences, for example participants' references to particular mindfulness practices or exercises, and to respond with confidence. Some interviewers had positive personal experiences of mindfulness whereas others held more neutral attitudes. None were aware of the main trial outcomes at the time the interviews were conducted and all were encouraged to adopt an open minded and curious attitude, with no preconceptions about whether MBCT-TS had, or had not, supported participants in their treatment journeys. Despite this, it should be acknowledged that some interviewers may have held implicit biases or expectations regarding treatment effects. Likewise, participants understood the association of interviewers with the primary trial. Thus whilst participants were encouraged to speak freely and honestly about their experiences, it is possible that their responses were influenced by the perceived allegiance of the researchers to

For peer review only

the intervention being explored, and that they may have felt a sense of obligation to make positive comments about MBCT-TS.

The researchers conducting data analysis, AT (BA Hons, Postgraduate Research Assistant) and CC (DPhil, Senior Research Fellow), although not involved in the PREVENT trial, had a knowledge of the programme under investigation. AT has undertaken mindfulness training in other contexts, and is familiar with the MBCT curriculum. CC has significant prior experience as a participant-observer in MBCT classes for people with recurrent depression. JR (DPhil, Lecturer) is an experienced qualitative researcher who has theoretical knowledge of mindfulness-based approaches. AT, CC and JR have all worked previously on studies exploring mindfulness-based programmes in different contexts and AT and CC were employed on a research grant exploring the effectiveness of mindfulness-based programmes at the time this work was conducted. AT, CC, and JR were aware of the main outcomes of the PREVENT trial (no superiority of MBCT-TS over maintenance antidepressants) at the time the qualitative analysis commenced, and approached the data with an assumption of overall equipoise between the two approaches, that was nevertheless likely to concealed marked individual differences in response. WK (PhD, DClinPsy) was the Principal Investigator on the PREVENT trial and is a mindfulness trainer and practitioner. He was not involved in directly teaching mindfulness to any of the participants in the PREVENT trial, but did supervise the mindfulness teachers who taught the MBCT-TS trial classes. He had no personal knowledge of the individual participants and their treatment journeys.

For peer review only

Consolidated criteria for reporting qualitative studies (COREQ): 32-item checklist

**No**	**Item**	**Guide questions/description**	**Responses and reference in manuscript if appropriate**
**Domain 1: Research team and reflexivity**			
Personal Characteristics			
1.	Interviewer/facilitator	Which author/s conducted the interview or focus group?	Contributor statement
2.	Credentials	What were the researcher's credentials? *E.g. PhD, MD*	AT, BSc; RB, MD; CC, PhD; FG, PhD; RH PhD; JC DClinPsy; AW DClinPsy; NM, PhD; WK, PhD.
3.	Occupation	What was their occupation at the time of the study?	All researchers. Title page, affiliation.

For peer review only

No	Item	Guide questions/description	Responses and reference in manuscript if appropriate
4.	Gender	Was the researcher male or female?	Female, AT, CC, RH, JC, AW, NM; Male RB, FG, WK.
5.	Experience and training	What experience or training did the researcher have?	Lead authors all had extensive research training, interviewers had interview training and RB, FG, NM and WK all had qualitative methods experience and training. See Methods and Statement Concerning Reflexivity

For peer review only

No	Item	Guide questions/description	Responses and reference in manuscript if appropriate
Relationship with participants			
6.	Relationship established	Was a relationship established prior to study commencement?	Yes. Manuscript Pages 12-13 and Statement Concerning Reflexivity
7.	Participant knowledge of the interviewer	What did the participants know about the researcher? e.g. *personal goals, reasons for doing the research*	Their role in the larger parent trial. See Interview Schedule and Statement Concerning Reflexivity.
8.	Interviewer characteristics	What characteristics were reported about the interviewer/facilitator? e.g. *Bias, assumptions, reasons and interests in the research topic*	See Interview Schedule and Statement

For peer review only

No	Item	Guide questions/description	Responses and reference in manuscript if appropriate
			Concerning Reflexivity.
**Domain 2: study design**			
Theoretical framework			
9.	Methodological orientation and Theory	What methodological orientation was stated to underpin the study? *e.g. grounded theory, discourse analysis, ethnography, phenomenology, content analysis*	Thematic analysis and case studies. Manuscript, Data Analysis) Pages 13-14)
Participant selection			
10.	Sampling	How were participants selected? *e.g. purposive, convenience, consecutive, snowball*	Purposive, see Manuscript, Methods, Participants, Pages 9-11.

For peer review only

No	Item	Guide questions/description	Responses and reference in manuscript if appropriate
11.	Method of approach	How were participants approached? e.*g. face-to-face, telephone, mail, email*	See Manuscript, Study Context and Participants, Pages 9-11.
12.	Sample size	How many participants were in the study?	See Manuscript, Study Context and Participants, Pages 9-11.
13.	Non-participation	How many people refused to participate or dropped out? Reasons?	See Manuscript, Study Context and Participants, Pages 9-11.
Setting			
14.	Setting of data collection	Where was the data collected? e.*g. home, clinic, workplace*	See Manuscript, Study Methods, Pages 9-13 as well as published Study

No	Item	Guide questions/description	Responses and reference in manuscript if appropriate
			Protocol and parent Trial.
15.	Presence of non-participants	Was anyone else present besides the participants and researchers?	No
16.	Description of sample	What are the important characteristics of the sample? *e.g. demographic data, date*	See Manuscript, Study Context and Participants, Pages 9-11.
Data collection			
17.	Interview guide	Were questions, prompts, guides provided by the authors? Was it pilot tested?	See Manuscript, Study Methods, Qualitative Data Collection pages 12-13, as well as the Supplementary Materials which includes both the

For peer review only

No	Item	Guide questions/description	Responses and reference in manuscript if appropriate
			Feedback Booklets and interview Schedules.
18.	Repeat interviews	Were repeat interviews carried out? If yes, how many?	No
19.	Audio/visual recording	Did the research use audio or visual recording to collect the data?	See Manuscript, Study Methods page 13
20.	Field notes	Were field notes made during and/or after the interview or focus group?	No
21.	Duration	What was the duration of the interviews or focus group?	See Manuscript, Study Methods, Interviews, page 13
22.	Data saturation	Was data saturation discussed?	Yes, see Study Protocol

For peer review only

No	Item	Guide questions/description	Responses and reference in manuscript if appropriate
23.	Transcripts returned	Were transcripts returned to participants for comment and/or correction?	No
**Domain 3: analysis and findings**			
Data analysis			
24.	Number of data coders	How many data coders coded the data?	See Manuscript, Data Analysis, pages 13-14
25.	Description of the coding tree	Did authors provide a description of the coding tree?	See Manuscript, Data Analysis, pages 13-14
26.	Derivation of themes	Were themes identified in advance or derived from the data?	See Manuscript, Data Analysis, pages 13-14

For peer review only

No	Item	Guide questions/description	Responses and reference in manuscript if appropriate
27.	Software	What software, if applicable, was used to manage the data?	See Manuscript, Data Analysis, pages 13-14
28.	Participant checking	Did participants provide feedback on the findings?	No
Reporting			
29.	Quotations presented	Were participant quotations presented to illustrate the themes / findings? Was each quotation identified? e.*g. participant number*	See Manuscript, Results Pages 15-26 and Case Studies, Box 1.
30.	Data and findings consistent	Was there consistency between the data presented and the findings?	See Manuscript, Results Pages 15-26 .
31.	Clarity of major themes	Were major themes clearly presented in the findings?	See Manuscript, Results Pages 15-26.

For peer review only

No	Item	Guide questions/description	Responses and reference in manuscript if appropriate
32.	Clarity of minor themes	Is there a description of diverse cases or discussion of minor themes?	See Manuscript, Results Pages 15-26.

For peer review only

PREVENT

1-month Feedback Booklet

For peer review only

telephone: 01392 726102 email: prevent@exeter.ac.uk web: www.prevent-southwest.org.uk
ISRCTN26666654 EudraCT number: 2009-012428-10
In collaboration with The University of Bristol, King's College London and the Medical Research Council Cognition and Brain Sciences Unit. Funded by National Institute for Health Research HTA

For peer review only - http://bmjopen.bmj.com/site/about/guidelines.xhtml

PREVENT Feedback Booklet

As researchers, we are very aware that we are working with people. In this case, a specific group of people who have all suffered from depression at times in their lives. We are careful to never lose sight of the fact that each person is also an individual with their own personality, feelings, and experiences.

One of our aims is to find out about the thoughts, feelings and experiences of the people taking part in this research project at two time points: 4-6 weeks after your final session of MBCT, and again at the end of your involvement in the project. We want to hear your individual views because we think that we can learn a lot from them.

Please take time to think about the questions in this booklet and answer them in your own words. It might be useful to read through the questions before writing your answers, in order to become familiar with them. Tell us exactly what you think! We won't be offended if you didn't like some parts, or even all, of the project. If you found some or all of it useful, helpful or enjoyable, then tell us that too, and please tell us why.

There are 11 questions in the booklet. Write as much, or as little as you like. If you feel that you have more to say than there is space for then please call us on 01392 726102 and we'll provide you with a second booklet.

As we have said in the past, we guarantee your privacy and confidentiality. Nothing that you say here will be published or printed in any way that could identify you, and no unauthorised person will ever read this booklet.

Once you have completed the booklet, please return it to us in the envelope provided. The envelope has a freepost sticker on it, so you do not need to pay for postage.

Finally, many thanks for the effort you have put in to this project so far. Hopefully our research will enable us to develop new and better ways to help people stay well after depression.

The PREVENT Team

For peer review only

PREVENT Trial ID: __________

Reducing your antidepressants

The first set of questions aims to explore your **thoughts** towards, and **experiences** of, taking and reducing your antidepressant medication.

1. Please describe your experiences of using antidepressants before taking part in the MBCT course. For example, did you feel that antidepressants were having a positive impact upon your day-to-day life and functioning? Did you experience any difficulties taking antidepressants in the past? Please explain why.

....
....
....
....
....
....

2. During the MBCT course you were asked to try to reduce your use of antidepressants. Please can you describe what your **thoughts** were towards reducing your antidepressants directly before the MBCT course started and again during the MBCT course when you were asked to try reducing them?

Thoughts about reducing antidepressants *before* the MBCT course:

....
....
....
....
....
....

Thoughts about reducing antidepressants *during* the MBCT course:

....
....
....
....
....
....

For peer review only - http://bmjopen.bmj.com/site/about/guidelines.xhtml

2. Can you please describe your **experiences** of reducing your use of antidepressants during the MBCT course up until this point now?
For example, have you started to reduce your antidepressants? (If YES, please continue below. If NO, please go to Question 3)

When did you start reducing your antidepressants? (Please be as specific as possible about the date)

....

What positive impact, if any, has reducing your antidepressants had on your day-to-day life and functioning?

....
....
....
....

What difficulties, if any, did you experience when reducing your antidepressants?

....
....
....
....
....
....

Did you do anything in particular to overcome these difficulties?

....
....
....
....
....
....

For peer review only

If you have tried reducing your antidepressants in the past, please describe if your experiences of reducing your antidepressants recently has been different, and if so, in what ways. If there has been no difference, please let us know.

………

………

………

………

………

………

3. It would be useful for us to know more about why people who take part in this research study do not reduce their antidepressants. Please could you describe why you have not reduced your antidepressant medication at this stage in the research study?

………

………

………

………

………

………

………

………

For peer review only

Taking part in MBCT

The questions in this section aim to find out more about your experiences of taking part in MBCT, and continuing the MBCT exercises after the course.

4. Out of the 8 week course, how many MBCT sessions did you attend?

.... out of 8 sessions.

5. Did you attend the first MBCT follow-up session? YES / NO

6. What, if any, MBCT exercises are you still practicing? Please tick the ones you are still practicing and in the space provided write how often you practice (e.g. per week or per month).

- None
- Body scan
  ....
- Sitting meditation
  ....
- 10-min sit
  ....
- 20-min sit
  ....
- Silence with bells
  ....
- Breathing space (regular, 3 times a day)
  ....
- Breathing space ('coping' space)
  ....
- Mindful walking
  ....
- Stretch and breath
  ....
- Mindful activities (please specify what, and how often per week or month)
  ....
- Other (please specify)
  ....

....

For peer review only

For peer review only - http://bmjopen.bmj.com/site/about/guidelines.xhtml

7. Please describe anything that you may have found **helpful** about taking part in the MBCT course and practicing the mindfulness meditation exercises, **and why**.

Specific aspects of the MBCT course you found **helpful**, and why:

....

....

....

....

....

....

....

....

....

Specific aspects of the meditation practices and teachings that you found **helpful**, and why:

....

....

....

....

....

....

....

....

....

For peer review only

8. Please describe anything that you may have found **unhelpful** about taking part in the MBCT course and practicing the mindfulness meditation exercises, **and why**.

Specific aspects of the MBCT course that you found **unhelpful**, and why:

....

....

....

....

....

....

....

....

....

Specific aspects of the MBCT practices and teachings that you found **unhelpful**, and why:

....

....

....

....

....

....

....

....

....

For peer review only

The Impact of MBCT

The final two questions relate to the **impact**, if any, that MBCT may have had on you.

9. Have you benefitted from the MBCT course and practicing the MBCT exercises? If so, in what ways? (We are interested to hear about benefits not only in how you feel, but in any other areas of your life). If there have been no benefits or that some things are worse now, please tell us about these.

....

....

....

....

....

....

....

....

....

....

....

....

....

10. Do you feel that MBCT **works for you**? If so, why do you think it works? If it doesn't work for you, please tell us why you think it doesn't work.

....

....

....

....

....

....

....

....

....

....

....

For peer review only

Any other comments:

11. Do you have any other comments that you would like to share about your experiences of taking part in this project?

....

....

....

....

....

....

....

....

....

....

....

....

....

....

For peer review only

For peer review only - http://bmjopen.bmj.com/site/about/guidelines.xhtml

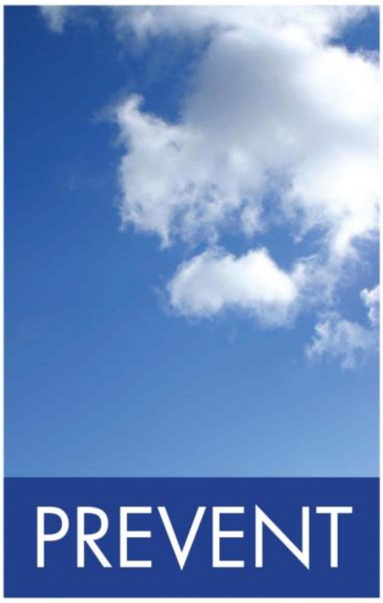

24-month Feedback Booklet

For peer review only

UNIVERSITY OF EXETER
telephone: 01392 726102 email: prevent@exeter.ac.uk web: www.prevent-southwest.org.uk
ISRCTN26666654 EudraCT number: 2009-012428-10
In collaboration with The University of Bristol, King's College London and the Medical Research Council Cognition and Brain Sciences Unit. Funded by National Institute for Health Research HTA
For peer review only - http://bmjopen.bmj.com/site/about/guidelines.xhtml
NHS

PREVENT 24-month Feedback Booklet

As researchers, we are very aware that we are working with people. In this case, a specific group of people who have all suffered from depression at times in their lives. We are careful to never lose sight of the fact that each person is also an individual with their own personality, feelings, and experiences.

One of our aims is to find out about the thoughts, feelings and experiences of the people taking part in this research project. We asked you to complete a feedback booklet in the weeks after attending MBCT, and we would be really grateful if you could now complete a second feedback booklet to explore your experiences of taking part in the project over the past two years. We want to hear your individual views because we think that we can learn a lot from them.

Please take time to think about the questions in this booklet and answer them in your own words. It might be useful to read through the questions before writing your answers, in order to become familiar with them. Tell us exactly what you think! We won't be offended if you didn't like some parts, or even all, of the project. If you found some or all of it useful, helpful or enjoyable, then tell us that too, and please tell us why.

There are 4 parts to this booklet and 27 questions in total, although not all of them will apply to you. Please read the instructions throughout the booklet to check which questions are relevant to you.

Please write as much, or as little, as you like. If you feel that you have more to say than there is space for then please use the back sheet to write any additional feedback.

As we have said in the past, we guarantee your privacy and confidentiality. Nothing that you say here will be published or printed in any way that could identify you, and no unauthorised person will ever read this booklet.

Once you have completed the booklet please give it to your PREVENT researcher at the 24-month assessment.

Finally, many thanks for the effort you have put in to this project. Hopefully our research will enable us to develop new and better ways to help people stay well after depression.

The PREVENT Team

PREVENT Trial ID: _________

For peer review only - http://bmjopen.bmj.com/site/about/guidelines.xhtml

Part I: Experiences of Reunions

Following your MBCT course you were invited to attend four MBCT reunion sessions.

1. How many did you attend? (Please circle)

None 1 2 3 4

2. Were there any specific reasons for attending this number of reunion sessions?

....
....
....
....
....
....
....

If you didn't attend any reunions, please go to Part II on page 4.

If you attended some or all reunion sessions, please continue below.

3. Please describe what you found helpful and / or unhelpful about the reunion sessions?

....
....
....
....
....
....
....

For peer review only

4. Have you used any other support to maintain your mindfulness practice (for example, keeping in contact with anyone from your MBCT course, or joining local meditation groups)? Please describe how this has been helpful and / or unhelpful and why.

....

....

....

....

....

....

....

For peer review only

Part II: Doing Mindfulness Practices

Which of the following statements best describes your use of mindfulness techniques (both formal practices and other mindful activities) over the past two years? Please tick one box and then follow the instructions for which question to complete next:

☐ I've used mindfulness techniques regularly over the last 2 years (please continue to Question 5)

☐ I used mindfulness techniques for a while after the MBCT course but my practice has tailed off and I no longer practice (please continue to Question 5)

☐ I've used mindfulness techniques off and on during the last 2 years (please continue to Question 5)

☐ I have not used mindfulness techniques at all since the MBCT course (please go to Question 7)

For peer review only

5. Please complete the two tables below to tell us which techniques you have practiced over the past two years and what you are currently practicing.

The following table provides a list of mindfulness practices from the MBCT course.

	Currently	Over the past two years
**Example: Body Scan**	*2* times per *week*	*Regularly for about 3 months after the course but then stopped. Restarted 2 months ago.*
**Body Scan**	times per	
**Sitting Meditation**	times per	
**10-minute sit**	times per	
**20-minute sit**	times per	
**Silence with Bells**	times per	
**Breathing Space (Regular, e.g. 3 times a day)**	times per	
**Breathing Space ('Coping' space)**	times per	
**Mindful Walking**	times per	
**Mindful Movement / Yoga**	times per	

For peer review only

The following table gives you space to record any mindful activities you've practiced.

Mindful Activities	Currently	Over the past two years
E.g., being mindful when showering	*Every morning*	*Every morning, although I stopped for roughly 3 months about a year ago.*

6. Have you adapted any of the mindfulness exercises you learned in the MBCT course to suit you? If so, how? Why did you make these changes?

....
....
....
....
....
....
....

7. If you do not currently practice any mindfulness techniques, please describe why.

....
....
....
....
....
....

For peer review only

Part III: The impact of MBCT and mindfulness practice

8. Do you feel that MBCT "works" for you? If so, how do you think it works? If you would like to, please use examples from your life.

....
....
....
....
....
....
....

9. What is the single most important thing that you've learned through attending the MBCT course or practicing mindfulness techniques? Please explain your answer.

....
....
....
....
....
....
....

10. MBCT may not be the right treatment for everyone. If you feel that MBCT was not right for you, please tell us why.

....
....
....
....
....
....
....

For peer review only

11. What changes, if any, would you make to the MBCT course and / or mindfulness exercises to make them more suitable for you?

....

....

....

....

....

....

....

....

....

For peer review only

Part IV: Taking antidepressants

We are interested in learning more about your experiences of taking and reducing antidepressants over the past two years.

12. Have your thoughts about reducing your antidepressants changed over the past two years? If so, in what ways?

....

....

....

....

....

....

13. Does taking antidepressant medication "work" for you? If so, please tell us why. If taking antidepressants does not "work" for you, please also tell us why. If you would like to, please use examples from your life.

....

....

....

....

....

....

14. What role has your GP had in your use of antidepressant medication over the past two years? How have you found this?

....

....

....

....

....

....

For peer review only

15. Has anyone else been involved in your use of antidepressant medication over the past two years? (E.g., psychiatrist, family member, another practitioner). If so, in what ways?

....
....
....
....
....
....

Which of the following best describes your use of antidepressants over the last two years? Please tick one box and follow the instructions for which questions to complete next.

☐ I have continued using antidepressants and have not reduced my use at any point over the past two years that I've been involved in this study. (Tick this box even if you have changed to a different antidepressant, or increased the dosage of your antidepressant.)

Please answer the questions in the ◆ box on the following page.

☐ I am in the process of reducing my antidepressants, or have reduced/stopped my use of antidepressants at some point over the past two years that I've been involved in this study. (Tick this box even if you restarted or increased your medication at a later date.)

Please answer the questions in the ● box on page 12

For peer review only

16. In your day-to-day life and functioning over the past two years, have you experienced any positive effects of continuing to use antidepressants? If so, please describe these.

17. In your day-to-day life and functioning over the past two years, have you experienced any negative effects or difficulties in continuing to use antidepressants? If so, please describe these.

18. Have you done anything specific to overcome these negative effects or difficulties? If so, what have you done?

19. Please describe why you haven't reduced your antidepressant medication over the past two years.

Please now go to Question 27 on page 11.

20. Has reducing your use of antidepressants had any positive effects on your day-to-day life and functioning over the past two years? If so, please describe how.

....
....
....
....
....

21. Have you experienced any negative effects or difficulties associated with reducing your antidepressants over the past two years? If so, please describe them.

....
....
....
....
....

22. Have you done anything specific to overcome these negative effects or difficulties? If so, what have you done?

....
....
....
....
....

23. Has practicing mindfulness techniques helped with these negative effects or difficulties? If so, in what ways?

....
....
....
....
....

Continued...

Continued...

24. Have you stopped using antidepressant medication completely, now or at any point in the last two years? Please describe your experiences of this.

....

....

....

....

....

25. Have you tried reducing your antidepressants at any point before this study? If so, please tell us of any ways that reducing your antidepressants over the past two years been different? If there has been no difference, why do you think this is the case?

....

....

....

....

....

26. Have you reduced or stopped your antidepressants over the past two years and then restarted or increased them again? If so, please tell us about this, and how you felt about this?

....

....

....

....

....

Please go to Question 27 on the following page.

Any other comments?

27. **Thank you for taking the time to complete this booklet - your participation is greatly appreciated!** If you have any other comments that you would like to share about your experiences of taking part in this project which did not fit into any of the answer spaces above, please use the space below.

For peer review only

PREVENT: End of Trial Interviews
Guidance for Interviewers

Aims

Researchers will conduct interviews with 42 purposively sampled participants in the MBCT arm of the trial at the end of the trial. The research topic we are targeting here is:
Participants' experiences during the follow-up period of the PREVENT trial of use of ADMs and MBCT-based techniques in relation to each other, during periods of wellness, depressive relapse, and transitions between these two.

Interview Preparation

Before conducting any of the interviews researchers read the key qualitative papers (Malpass et al., 2012; Allen et al., 2009). Before each interview researcher to read the participant's file to (re-) familiarise themselves with participant characteristics (e.g., relationship status) and obtain timeline populated with info on borderline or actual relapses and ADM usage. They should also review, and have to hand in the interview, the end of treatment and 24-month feedback booklets to get a sense of the profile of each participant during the follow-up period in terms of the following variables: full or partial attendance of MBCT course; use of mindfulness techniques; use of ADMs; and any significant life events. This will shape the interview in terms of: i) which sub-sections of the interview are relevant; ii) issues for particular participants that may need to be probed in more detail.
Make sure you are as clear as possible about which sections of the interview and questions are relevant to your respondent BEFORE YOU START. Where possible, **choose specific/representative/salient episodes or junctures** in timeline for targeted probing using the schedule (possibly match them onto wellness, wobbles, and depressive sections). **Print out two copies of timeline**, one for yourself and one for the interviewee to draw upon.

Conducting the Interview

The interview is semi-structured. In each section, questions and follow-up questions are suggested. However, researchers should use their judgment in drawing out participants in relation to the research topic. Interviewers ask open-ended questions and follow participants' "leads" while keeping in mind the research question. The aim is to enable participants to give their "story" around the main topics of investigation.
Researchers should be strategic in use of time in the interview to ensure the topic guide/respondent covers material that answers the research questions; especially in Section 1, it is important to keep the interview focused. If the interviewer feels respondents are being unclear or opaque help the respondent unpack what they mean or agree with the participant that the issue is unclear. Interviewers should ensure *that all the interview questions that are relevant to the participants' particular profile are covered.* Consider a temporal order to do the interview allocating/planning pre-set time windows for each consecutive episode/junction so as not to exceed a **maximum of 60 minutes** for the overall interview.

Participants may feel the need to please researchers or give the "right" answers. This is particularly likely around ADM tapering where the trial has consistently communicated that we want participants to taper and discontinue their ADM. It is important that researchers communicate explicitly and non-

For peer review only - http://bmjopen.bmj.com/site/about/guidelines.xhtml

verbally that there are no right and wrong answers to any of the questions and we want to know people's experiences in relation to MBCT and ADM.

Use of particular terms (e.g., "wobble," "relapse") can be adapted in the light of language used by participants. Follow-up questions should be asked for clarification and elaboration, *and this should be driven by the research aim for each section of the interview*. As far as possible interviewers should cover all the suggested topics and questions and behave with the minimum of variation between interviews. Instructions to interviewers and suggested wordings for introductions to each section are given in italics below. The key issues for each question are in bold.

Interview Opening / Introduction

Open the interview with something like:

"Thank you for agreeing to do this interview with me. The interview is about hearing from you how things have been in the trial and checking that we have your story right. It will be collaborative and semi-structured which means I have some topics I'd like to cover, but the questions will be quite open so as to give you, as an expert of your own lived experience, a chance to express your own views and tell me about your experiences and what seems important to you. Everyone will have had different experiences and we are interested in hearing about these from your point of view – both the good and the not-so-good experiences – so that we can learn about and develop our treatments further. Please tell us exactly how you feel!

Just to let you know that I am un-blinded and know that you have participated in the MBCT group, so it is ok to talk freely about this. I would like to record this interview, and the recording will be transcribed word for word and analysed as part of the research for the PREVENT trial. All identifying information will be removed at this point. Your name will never appear on the transcription or any other documents or files that result from this interview (such as the audio file). Do you have any questions before we begin?"

Section 1: Overview of the follow-up period using the timeline

The aim of this section is to obtain a brief overview of how the participant has experienced the follow-up period in terms of three areas: 1. Periods of wellness and depression; 2. Antidepressant medication; 3. Life events. Subsequent interview sections follow up on each of these in more detail and the profile of experiences here can be used to tailor subsequent interview questions. Information on the first two areas should be available in advance and summarised on the prepared timeline. Information on life events might be found in the research files and database but will need to be obtained here. Preparation by thoroughly reading the file, and possibly even some of the audio recordings, in advance will help a lot here.

Use the timeline in whatever way feels most comfortable and guided by the respondent's preference. The participant can add life events him/herself; or the researcher can do this; or it can simply be used as a guide. Show the timeline and say something like:

"I'd like to start with a brief overview of how life has been for you in the trial. In preparing for our meeting today, we have put together this timeline. It summarises the information you have given us as part of the research study about any periods of depression or wobbles, and your use of antidepressant medication during the trial. It also includes the date of your most recent episode of depression before taking part in the trial, based upon what you told us during your first assessment. I've also

For peer review only - http://bmjopen.bmj.com/site/about/guidelines.xhtml

looked through your comments in the Feedback Booklets you completed one month after the end of the mindfulness course and again recently. These were very helpful and I'll use some of what you wrote there to guide what we talk about today."

Interview Question	Probes/Examples/Directions
1.1. Do you think the information we have here on the timeline looks about right? Is there anything you would like to change?	If we know from previous assessments that the person has had a significant life event ensure this is acknowledged here. Mark – or get the respondent to mark – any life events on the time-line / graph.
1.2. Have there been any important events in your life during your time in the trial that have affected you either positively or negatively that we can add to the timeline?	Keep the discussion focused and brief to allow as much time for later sections as possible.
1.3. Can we check that I have your use of antidepressant medication right these last two years?	Work through timeline for any tapering/discontinuation, dosage, resumption etc.
1.4. How have things been between the last time we spoke and today?	Informally extend timeline with relevant depressive episodes/ADM use/life events up until current interview date; Where necessary, establish current symptomatic status (informally) and make sure they are ok to continue.

"The remaining questions in this interview will focus upon your experiences with the aid of this timeline."

Keep the time-line / graph in view to refer back to / use further in later parts of the interview. Use the timeline to keep the interview contained with regard to the different episodes/junctures identified prior to interview. Allow and encourage the participant to use a pen to put down details in their copy of the timeline.

Section 2: Questions on wellness (for all)

Comment on Section 2-4: Particularly in the wobbles section it is important to go for depth rather than breadth with regards to potential issues around these time points (e.g. what happened in days before, relationships, sleep etc.). As a general principle, interviewers should encourage interviewees to focus on prototypical or most memorable junctures in timeline. In order to keep interview contained, agree on a timeframe for each episode/juncture and keep questions focused on this.

"I'd like to ask about any times when you were well during the trial – when you weren't feeling low or experiencing an episode of depression"

For peer review only

Interview Question	Probes/Examples/Directions
2.1. Has anything from the mindfulness course played a part in staying well during the trial? If so, can you describe how?	Examples: any techniques, ideas; response plan<br>Prompts: How / why / why not?; Therapist/Group/Researcher Role
2.2 Did your use of antidepressants play a part in staying well during the trial? If so, can you describe how?	Prompts: How / why / why not? ; GP Role
2.3 What about the combination of xxx from the mindfulness course and use of antidepressants in periods of wellness?	[ask this additional question if respondent says that both MBCT and ADM have had some value in questions 2.2 and 2.3., otherwise skip]

Section 3: Question on wobbles / early signs of depression (for all)

"I'd like to ask about any times when you were well, but you felt yourself starting to 'wobble' or feel low. So any times when you might have felt that your mood was dipping or you were starting to have more of the negative thoughts that were around when you were depressed"

Interview Question	Probes/Examples/Directions
3.1. How did these experiences of 'wobbles' or starting to feel low compare to previous experiences of wobbles?	Use timeline to go right into the situation shortly before, during and directly after episode.<br>Probe: What happened, how was it different? What did you do? How did you get out of it?
3.2. Was anything from the mindfulness course useful at the time of wobbling or when starting to feel low during the trial? If so, can you describe how?	Probe: before/during/after wobble<br>Examples: any techniques, ideas; response plan, self-compassion etc.<br>Prompts: How / why / why not?
3.3 Did your use of antidepressants during the trial play a part in wobbling or starting to feel low during the trial? If so, can you describe how?	Prompts: How / why / why not?
3.4 What about the combination of xxx from the mindfulness course and use of antidepressants in periods of wobbling or when starting to feel low?	[ask this additional question if respondent says that both MBCT and ADM have had some value, otherwise skip as appropriate]

For peer review only - http://bmjopen.bmj.com/site/about/guidelines.xhtml

Section 4: Experiences of depressive relapse (for those who have relapsed)

Use the timeline to guide question choices in relation to experiences of relapse.

Say: "I'd like to ask you about your experiences this / these episode of depression..."

Interview Question	Probes/Examples/Directions
4.1. How did this episode of depression compare to previous episodes of depression?	Use timeline to take them right into the situation shortly before, during and directly after episode. Probe: What happened, how was it different? What did you do? How did you get out of it?
4.2. Was anything from the mindfulness course useful at the time of depression? If so, can you describe how?	Examples: any techniques, ideas; response plan<br>Prompts: How / why / why not?
4.3. Did your use of antidepressants during the trial play a part in this episode of depression? If so, can you describe how?	Prompts: How / why / why not?
4.4 What about the combination of xxx from the mindfulness course and use of antidepressants in periods of depression?	[ask this additional question if respondent says that both MBCT and ADM have had some value, otherwise skip as appropriate]

Section 5: MBCT and ADMs combined

"I'd like to end with some open questions about your experiences of taking part in this study."

For sakes of time keeping and for keeping this section contained it is critical how the questions are set up. Encourage people to step back from and reflect critically on their own experience rather than letting them share their raw experience. Use formulations like the following: *'I am sure that this was an intensive time for you. If you took yourself away from the experience today and were to reflect on this, what may I ask are the key elements/thoughts that you would have with regards to staying well etc.?'*

Interview Question	Probes/Examples/Directions
5.1. Has taking part in the trial changed the way you think about depression? If so, could you tell me how?	Probe: How/why /why not?<br>Probe: Has it changed the way you think about the causes/consequences of depression? Role of GP.
5.2. Has taking part in the trial changed how you think about yourself? If so, could you tell me how?	Probe: how identity was before the trial if person describes a sense of change.<br>Examples: Role of group, immediate and wider social

For peer review only - http://bmjopen.bmj.com/site/about/guidelines.xhtml

Interview Question	Probes/Examples/Directions
	environment
5.3. Now that you have had experiences of both mindfulness and antidepressants, what do you think of each of them as treatments for depression?	Probes: do you favour one over the other? In combination?<br>At different points e.g. in wellness, wobbles and depressive episodes?<br>For prevention? For recovery?
5.4. Apart from depression, has taking part in the trial had any impact on other psychological or physical health problems you may have?	Examples:<br>Anxieties/phobias, Chronic health conditions like pain, diabetes etc.

Section 6: Ending

"I've covered all the questions we have, but before we end, is there anything you would like to add?"

Interview Question	Probes/Examples/Directions
6.1. …about any of the topics we have discussed?	
6.2. …about any of your experiences of depression, mindfulness or antidepressant medication that we have not talked about?	
6.3. …about anything else you think is relevant to this project on MBCT, antidepressants, and depression?	

End by thanking the respondent for their time and for sharing their views and experiences. Reiterate how valuable this is for research trying to develop treatments for depression. Inform them about further dissemination of PREVENT findings.

For peer review only - http://bmjopen.bmj.com/site/about/guidelines.xhtml

COREQ (COnsolidated criteria for REporting Qualitative research) Checklist

A checklist of items that should be included in reports of qualitative research. You must report the page number in your manuscript where you consider each of the items listed in this checklist. If you have not included this information, either revise your manuscript accordingly before submitting or note N/A.

Topic	Item No.	Guide Questions/Description	Reported on Page No.
**Domain 1: Research team and reflexivity**			
Personal characteristics			
Interviewer/facilitator	1	Which author/s conducted the interview or focus group?	2, 8
Credentials	2	What were the researcher's credentials? E.g. PhD, MD	8
Occupation	3	What was their occupation at the time of the study?	8
Gender	4	Was the researcher male or female?	8
Experience and training	5	What experience or training did the researcher have?	8
Relationship with participants			
Relationship established	6	Was a relationship established prior to study commencement?	8
Participant knowledge of the interviewer	7	What did the participants know about the researcher? e.g. personal goals, reasons for doing the research	8
Interviewer characteristics	8	What characteristics were reported about the inter viewer/facilitator? e.g. Bias, assumptions, reasons and interests in the research topic	8
**Domain 2: Study design**			
Theoretical framework			
Methodological orientation and Theory	9	What methodological orientation was stated to underpin the study? e.g. grounded theory, discourse analysis, ethnography, phenomenology, content analysis	13
Participant selection			
Sampling	10	How were participants selected? e.g. purposive, convenience, consecutive, snowball	8,9
Method of approach	11	How were participants approached? e.g. face-to-face, telephone, mail, email	8
Sample size	12	How many participants were in the study?	8,9
Non-participation	13	How many people refused to participate or dropped out? Reasons?	9
Setting			
Setting of data collection	14	Where was the data collected? e.g. home, clinic, workplace	11,12
Presence of non-participants	15	Was anyone else present besides the participants and researchers?	No
Description of sample	16	What are the important characteristics of the sample? e.g. demographic data, date	10
Data collection			
Interview guide	17	Were questions, prompts, guides provided by the authors? Was it pilot tested?	11,12
Repeat interviews	18	Were repeat inter views carried out? If yes, how many?	N/A
Audio/visual recording	19	Did the research use audio or visual recording to collect the data?	12
Field notes	20	Were field notes made during and/or after the inter view or focus group?	N/A
Duration	21	What was the duration of the inter views or focus group?	11
Data saturation	22	Was data saturation discussed?	N/A
Transcripts returned	23	Were transcripts returned to participants for comment and/or	N/A

For peer review only

Topic	Item No.	Guide Questions/Description	Reported on Page No.
		correction?	
**Domain 3: analysis and findings**			
Data analysis			
Number of data coders	24	How many data coders coded the data?	13
Description of the coding tree	25	Did authors provide a description of the coding tree?	14,15
Derivation of themes	26	Were themes identified in advance or derived from the data?	13
Software	27	What software, if applicable, was used to manage the data?	13
Participant checking	28	Did participants provide feedback on the findings?	N/A
Reporting			
Quotations presented	29	Were participant quotations presented to illustrate the themes/findings? Was each quotation identified? e.g. participant number	14-29
Data and findings consistent	30	Was there consistency between the data presented and the findings?	29-36
Clarity of major themes	31	Were major themes clearly presented in the findings?	29
Clarity of minor themes	32	Is there a description of diverse cases or discussion of minor themes?	39-42

Developed from: Tong A, Sainsbury P, Craig J. Consolidated criteria for reporting qualitative research (COREQ): a 32-item checklist for interviews and focus groups. *International Journal for Quality in Health Care*. 2007. Volume 19, Number 6: pp. 349 – 357

Once you have completed this checklist, please save a copy and upload it as part of your submission. DO NOT include this checklist as part of the main manuscript document. It must be uploaded as a separate file.

For peer review only
